# Development of a multilocus-based approach for sponge (phylum Porifera) identification: refinement and limitations

**DOI:** 10.1038/srep41422

**Published:** 2017-02-02

**Authors:** Qi Yang, Christopher M. M. Franco, Shirley J. Sorokin, Wei Zhang

**Affiliations:** 1 Centre for Marine Bioproducts Development, Flinders University, Adelaide, South Australia, SA 5042, Australia; 2 Department of Medical Biotechnology, School of Medicine, Faculty of Medicine, Nursing and Health Sciences, Flinders University, Adelaide, South Australia, SA 5042, Australia; 3 SARDI Aquatic Sciences, 2 Hamra Ave, West Beach, SA 5024, Australia; 4 Centre for Marine Drugs, Renji Hospital, Shanghai Jiaotong University, Shanghai, 200240, China

## Abstract

For sponges (phylum Porifera), there is no reliable molecular protocol available for species identification. To address this gap, we developed a multilocus-based Sponge Identification Protocol (SIP) validated by a sample of 37 sponge species belonging to 10 orders from South Australia. The universal barcode COI mtDNA, 28S rRNA gene (D3–D5), and the nuclear ITS1-5.8S-ITS2 region were evaluated for their suitability and capacity for sponge identification. The highest Bit Score was applied to infer the identity. The reliability of SIP was validated by phylogenetic analysis. The 28S rRNA gene and COI mtDNA performed better than the ITS region in classifying sponges at various taxonomic levels. A major limitation is that the databases are not well populated and possess low diversity, making it difficult to conduct the molecular identification protocol. The identification is also impacted by the accuracy of the morphological classification of the sponges whose sequences have been submitted to the database. Re-examination of the morphological identification further demonstrated and improved the reliability of sponge identification by SIP. Integrated with morphological identification, the multilocus-based SIP offers an improved protocol for more reliable and effective sponge identification, by coupling the accuracy of different DNA markers.

Sponges (phylum Porifera), the evolutionary oldest multicellular animals, are sessile, benthic filter-feeders^1^. In marine habitats, they are highly diverse and play important roles in biogeochemical cycling^1^, in the spatial structuring of the seafloor, and in the benthic-pelagic coupling of nutrient transfer within ocean ecosystems^2^. Sponges are also commercially important to the pharmaceutical and biomaterials industries as they participate in complex biotic interactions with diverse macrobiotic taxa^2^ and microbiological communities^3^ to produce up to 30% of all active marine metabolites found^4^.

According to the World Porifera Database^5^, there are more than 8,700 valid species, 7,300 of which belong to the class Demospongiae. Porifera are an important group of Metazoa in which species identification is particularly difficult because the available characters used for classification are limited^6^. For example, the sponge family Polymastiidae possesses a relatively simple spicule assortment providing a rather scant set of taxonomic characters^7^. Some features are in fact also displayed by some taxa from other families^7^. Generally, these characters are their organic and inorganic skeletons, including skeletal size, shape, structure and composition^6^. However, the arrangement of these skeletal elements can be inconsistent, and our understanding of the evolution of skeletal traits is incomplete^8^,^9^,^10^,^11^,^12^,^13^. Indeed, traditional morphological identification methods often lead to erroneous classification^14^,^15^, and the actual species diversity and distribution may be underestimated^16^.

Molecular approaches, such as DNA barcoding, provide a potential solution for sponge classification^17^,^18^,^19^. In our study, we applied DNA markers to assist in the identification of individuals against already known species, which consists of comparing standardised stretches of DNA (barcodes) to reference databases to identify sponges. The mitochondrial genome (mtDNA) exists in all eukaryotic cells and is a good marker for species identification because of its clonal (maternal) mode of inheritance and clock-like evolutionary rate^20^,^21^. It has been used to study species identification, sponge diversification patterns^8^ as well as phylogenetic relationships^22^ with varying degrees of success^23^,^24^. The COI mtDNA locus is a conservative region but with highly variable sequences. It is the most commonly used mitochondrial marker of approximately 700 bp at the 5′ end of the cytochrome *c* oxidase subunit I gene (COI locus). This gene is relatively easy to amplify as it is conserved across multicellular animals^25^ and abundant in eukaryotic DNA^26^. Many studies reported that COI mtDNA successfully discriminated sponges at different taxonomic levels^11^,^12^,^13^,^14^. However, some species that can be clearly distinguished on the basis of morphology show similar COI sequences^8^,^27^,^28^. Studies on the COI intraspecific variation has been used more regularly to classify other metazoans at the species level, but less so for sponges^29^,^30^.

Slow mitochondrial evolution is a problem for the resolution of phylogenies at the species and genus levels using standard mitochondrial markers. However, the question remains as to whether faster evolving gene regions can be identified for use in conjunction with the standard COI mtDNA barcode^8^. The nuclear ribosomal genes of eukaryotes, such as the 28S (large subunit, LSU) rRNA genes^31^, are arranged in tandemly repeated clusters, where transcribed units alternate with non-transcribed units called spacers, such as the internal transcribed spacer 1 (ITS1) and 2 (ITS2)^32^. The 28S rRNA gene (28S locus) has regions that are sufficiently heterogeneous to address phylogeny at different levels^10^,^22^. For example, the regions D3-D5 have been used as the DNA markers for sponge taxonomy^12^,^33^. The more rapidly evolving ITS regions are commonly used as “high resolution” markers. In general, ITS regions are used to reconstruct relationships ranging from those between populations to those between the taxonomic “families”. They have been used for phylogenetic and phylogeographic analyses of non-bilaterian metazoans such as corals^34^ and sponges^35^.

Based on current knowledge about the resolution of different DNA markers, no single ideal marker for all sponge species exists, as each marker has its own strengths and limitations^20^,^29^,^30^,^36^. The incomplete sequence entries in the gene database limit the application of the phylogeny-based molecular taxonomic approach for species identification. In the NCBI database, the sponge derived gene submissions only cover a few hundred^37^ out of the known 8,700 sponge species. Therefore, we aim to establish an effective and practical multilocus-based molecular approach for sponge identification to respond to these issues. Our study, using South Australian sponges, was set up to (1) develop a Sponge Identification Protocol (SIP) using three DNA markers (the mitochondrial COI gene, the nuclear 28S rRNA gene and the nuclear ITS region); (2) validate the reliability of the SIP by phylogenetic analysis; (3) evaluate the efficiency of the proposed SIP in identifying 37 sponge species (three individuals for each); (4) approach a final identity by re-examining the morphological characters and mutual validation with SIP identification to discriminate the sponges whose identities are ambiguous; and (5) demonstrate the resolution and the suitability of different DNA markers in conjunction with morphological characters for sponge classification.

## Results

### Initial morphological classification

The 111 sponge individuals belonging to 37 potential species (three individuals for each species) were classified based on their morphological features. Supplementary Table S1 shows the classification, sampling dates, and their locations. All the 37 sponges could be identified at the order/family level. Thirty-one sponges were identified to genus and among of them nine were identified to species.

### Valid sequences

DNA from the 111 specimens belonging to 37 species was extracted successfully. For PCR amplification, the three duplicates of each species showed a consistent performance. Ninety-three COI mtDNA amplicons from 31 sponge species, 75 amplicons of 28S rRNA gene from 25 species, and 96 amplicons of ITS region from 32 species were successfully obtained. All the amplicons were sequenced to give 93 COI mtDNA sequences (31 species), 66 sequences of the 28S rRNA gene (22 species), and 81 ITS sequences (27 species) with a success rate of sequencing at 84%, 59%, and 73%, respectively. The alignment between the three duplicates of each species matched (>99% similarity) so that only one of the three was used for the following analysis.

**Figure 1:**
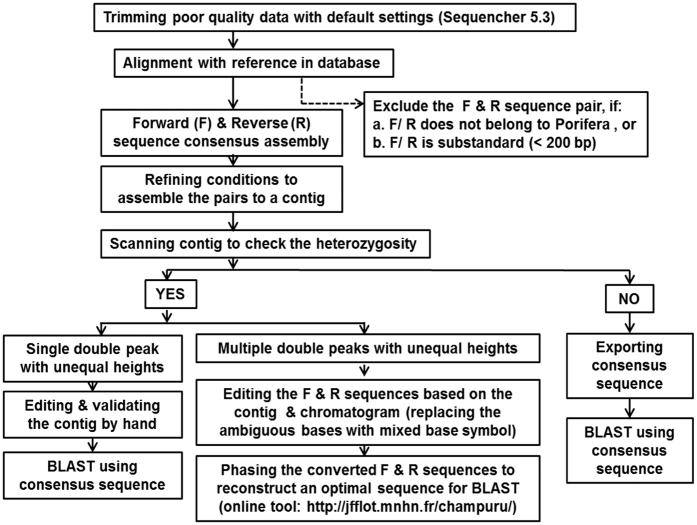
Sequence data processing flowchart.

The COI primers LCO1490 and HCO2198 yielded PCR products from 31 of the 37 potential sponge species (Supplementary Table S2). All the PCR products were sequenced with sizes ranging from 672–699 bp. Two of the six sponges with no PCR products for the COI locus had available sequence data from both 28S and ITS loci. The other four with sequence data only from the ITS locus were subjected to the alternative COI primer sets C1-J2165/C-Npor2760 and CO1porF1/CO1porR1 in a nested-PCR. However, no products were obtained. Implementing the data processing protocol in Fig. 1, 29 valid sequence pairs (forward and reverse) were obtained from the total of 31 successfully sequenced COI mtDNA amplicons (Supplementary Table S2). Two were excluded as the forward sequence of one did not belong to Porifera, and the other had only 170 bp of the forward sequence (Supplementary Table S3).

For the 28S rRNA gene, 25 PCR products were amplified from the 37 sponge species using primers NL4F/NL4R with sizes of 845–1227 bp, of which 22 were sequenced successfully (Supplementary Table S2). Of the 15 unsuccessfully sequenced sponges, the identities of three had the data from both COI and ITS loci. The rest (12 sponges) were subjected to the second pair of 28S primers, RD3A/RD5B2, which yielded PCR products from four of the 12 sponges (Supplementary Table S4). Of the 22 successfully sequenced 28S amplicons, two were excluded as their reverse readings showed identities not belonging to Porifera (Supplementary Table S3).

The nuclear ITS amplicons of 32 sponge species were successfully amplified by the primer set, ITSRA2/ITS2.2, giving PCR products ranging from 334–1142 bp (Supplementary Table S2). Of the 32 amplicons, 27 were sequenced successfully. Fifteen were excluded because the coverage percentage (the size of the sequence participating in the cluster analysis/the size of the whole query sequence) was less than 50%. The other 12 sequence pairs included 11 valid ones and one non-Porifera sequence.

### Identification using the proposed Sponge Identification Protocol (SIP)

After the data processing, 29, 20 and 11 valid sequence pairs, for which there was consensus between the sequence of their forward and reverse strands, were obtained, respectively, from the three DNA markers of the mitochondrial COI, the nuclear 28S rRNA gene and the nuclear ITS region (Supplementary Table S2). The three loci derived from the same sponge were given equal weightage.

**Figure 2:**
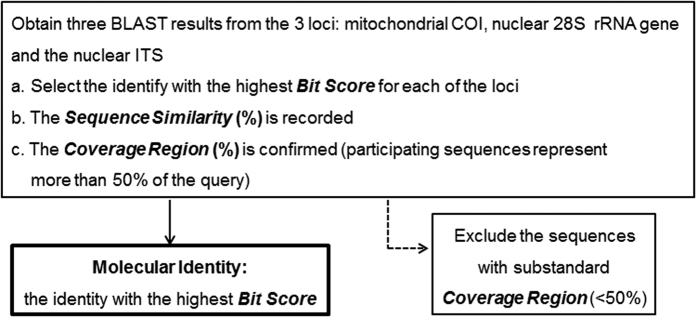
The Sponge Identification Protocol (SIP) flow diagram used in this study.

With the application of the proposed SIP in this study (Fig. 2), the closest matches against the entries in the Genbank database were obtained, and assigned to 34 out of the 37 sponges as the initial SIP identities (Table 1). The sponges with no sequencing results for the COI locus were checked to see whether they had a matching identity from both 28S and ITS nuclear loci (Supplementary Table S3). From the 29 valid COI sequences, 11 with the highest Bit Score among the three loci were used to infer the identities (Supplementary Table S2). Eight of the 11 sponges belong to eight distinct species in eight families in six orders, and the other three were identified as duplicate species (Table 1). For the 28S rRNA gene, of the 15 unsuccessfully sequenced sponges, the identities of three were inferred from their COI and ITS loci. Based on the 20 valid 28S rRNA gene sequences, 19 with the highest Bit Score were inferred as 15 different species in 15 families in 10 orders (Supplementary Table S2). Six of the 10 orders were already identified in the results from the COI locus analysis whereas four more were identified (Clionaida, Haplosclerida, Dictyoceratida and Tethyida) (Table 1). In regard to the ITS locus, 11 valid sequences were obtained (Supplementary Table S2), from which another four sponges were inferred as two different species. The family Halichondriidae in the order Suberitida, however, was only identified by it ITS locus (Table 1).

Overall, eighteen sponge species had valid sequencing data for both the COI mtDNA and 28S rRNA gene. Similarly, the COI and ITS loci were available for eight sponge species, and the 28S and ITS loci were available for six sponge species. Only five sponge species had sequencing results from all the three DNA loci.

### SIP reliability validated by phylogenetic analysis

In order to validate the reliability of the proposed SIP for sponge identification, the phylogenetic analysis was compared with the BLAST and the SIP identities (Table 2). The sponges have a cut-off sequence similarity of 96% as the sequences with lower similarity could not be aligned with other reference sequences to compute the valid phylogenetic relationship for identification. Furthermore, considering the minimum coverage percentage to have a valid sequence alignment, all of the ITS loci were excluded from the phylogenetic analysis due to their lower coverages.

**Figure 3:**
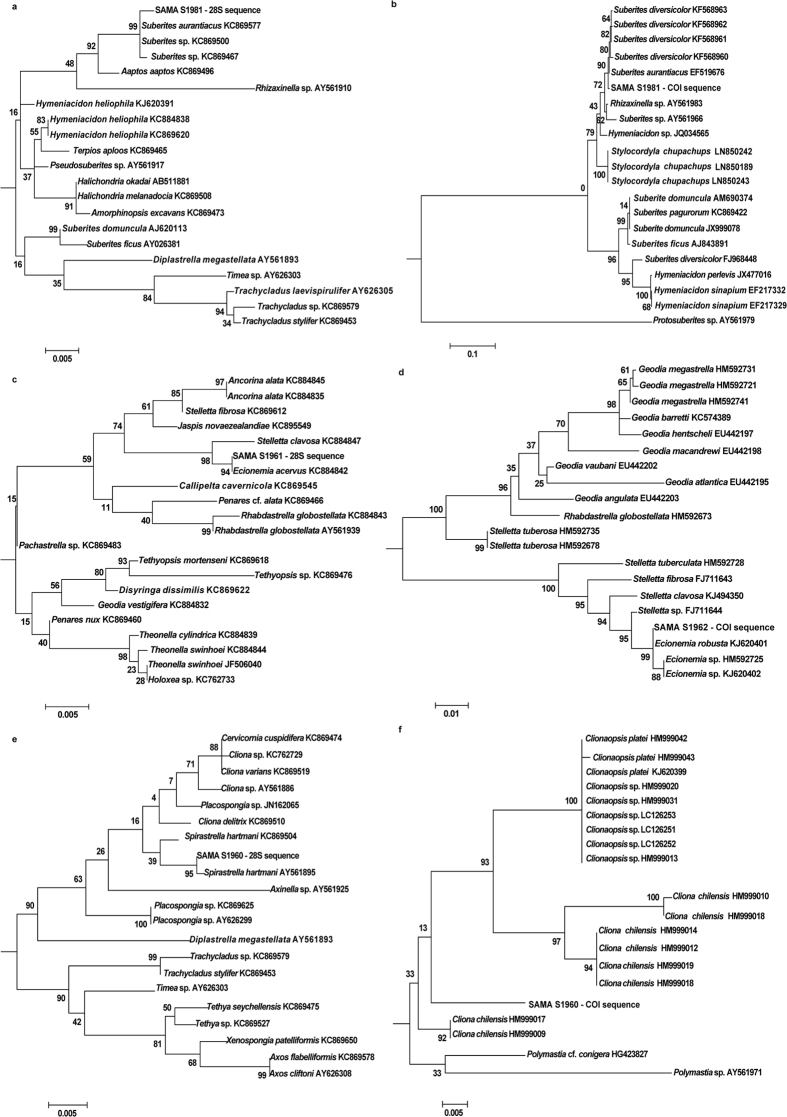
Phylogenetic relationship of three representative sponge species using the Maximum Likelihood method based on 28S rRNA gene and COI mtDNA to validate the sponge identification by SIP. (**a**) Phylogenetic relationship of sponge SAMA S1981 based on 28S rRNA gene. (**b**) Phylogenetic relationship of sponge SAMA S1981 based on COI mtDNA. (**c**) Phylogenetic relationship of sponge SAMA S1962 based on 28S rRNA gene. (**d**) Phylogenetic relationship of sponge SAMA S1962 based on COI mtDNA. (**e**) Phylogenetic relationship of sponge SAMA S1960 based on 28S rRNA gene. (**f**) Phylogenetic relationship of sponge SAMA S1960 based on COI mtDNA.

The analysis illustrated that the identities of all sponges concluded from SIP matched perfectly well with the phylogenetic analysis for both DNA markers, even though the BLAST results of the two loci were different (Supplementary Figs S1 and S2). For example, sponge SAMA S1981 was inferred to have the closest match with *Suberites aurantiacus* by SIP with the highest Bit Score among the three loci. The identity inferred from the BLAST result of 28S locus was different from the BLAST of COI locus, but they shared the same sequence similarity (98%). Using the phylogenetic analysis, it was demonstrated that the query sequence showed a closer relationship with the species *Suberites aurantiacus* in both of the trees for COI and 28S loci based on the Maximum Likelihood (Fig. 3a,b) and Neighbour Joining methods (Supplementary Figs S1 and S2), which validated the identity inferred by SIP. Similarly, sponge SAMA S1962 was inferred to have the closest match with *Ecionemia robusta* from the COI locus by SIP and confirmed by the phylogenetic analysis of COI (Fig. 3d) and 28S loci (Fig. 3c) at the genus level. The phylogenetic tree for the 28S locus showed that the query was closer to *Ecionemia acervus*, in the same genus as the SIP identity, instead of the BLAST result *Stelletta clavosa*. Checking the database, there was no 28S rRNA gene entry for the species *Ecionemia robusta*. For sponges (e.g. sponge SAMA S1960) where the same genus does not exist in the both COI and 28S databases, the trees showed the same phylogenetic status of the query sequence (Fig. 3e,f).

### Re-examination of morphological identifications

There were only nine sponge species identified to the same genus by both the SIP and morphological analysis. To resolve the discrepancy, the 34 sponge species inferred by SIP (Table 1) were re-examined based on their morphological features (Table 3). Morphological descriptions of the 34 sponge species are documented in Supplementary Table S5. Twenty-seven sponge species were assigned to the same classifications as their initial ones based on the available morphological characters. Based on both of the morphological and the SIP identification, the 27 sponge species were divided into four categories. Category I, nine sponge species of which the re-examined identities matched with the SIP identities at the genus level. Category II, seven sponge species matched only at the order or family level, but where the orders or families have highly similar morphological features. Category III, eight sponge species matched only at order level and Category IV (three species) where sponges did not match even at the order level.

Importantly, the initial morphological identities of seven sponge species were corrected and revised (Category V). Of these, when the morphological identities were re-examined three matched with the SIP identities at the genus level, and another three matched at the order level. Only one sponge species did not have a match even at the order level.

### Approach to the final identity and the improved discrimination

The differences between morphological classification and SIP identification occurred at various taxonomic levels (Table 3). The final identities were concluded by comprehensively considering the confidence level for both the molecular and morphological identification and following the rules in the “Materials and Methods- Morphological re-examination and final identification” section.

In Category I (Table 3), the final identities of all the nine sponge species was based on SIP as their identities generated by SIP matched the morphological identification at the genus level. In particular, the species *Ecionemia robusta* (SAMA S1962) inferred by SIP has been reported in South Australia. It was possible to achieve a species-level identity. In category II, the final identities of the seven sponge species followed the SIP identification due to their highly similar morphological features. For example, the SIP identity of the sponge SAMA S1989 was *Igernella notabilis* (Duchassaing & Michelotti, 1864), a fleshy pink conulose Caribbean sponge. The morphological ID gave *Aplysilla rosea* (Barrois, 1876), a fleshy pink conulose sponge from the NE Atlantic region and the Mediterranean area. van Soest^5^ stated that other records of *A. rosea* are inaccurate, although *A. rosea* is listed in Australia^38^ and commonly cited. Due to the need to revise the genus *Aplysilla*, it is possible for sponge SAMA S1989 to be corrected as *Igernella*.

In terms of the 18 sponges in categories III, IV, and V, their final identities were determined by the same rule by considering both SIP and morphological identification. Three sponge species matched identities at the genus level (Category V: SAMA S1979, S1985, and S1988). Therefore, their SIP identities were the final identification. Of the 15 remaining sponge species, where identification between morphology and SIP did not match at family or order levels, SIP identities were accepted for eight based on the threshold of ≥98% sequence similarity. The other seven were based on the morphological identification.

Overall, 27 of the 34 sponge species were finally identified by SIP and seven by morphology. These final identities of the 34 sponge species were assigned to the submissions of the three loci into the NCBI database.

Of note, five sponges identified initially as *Chondropsis* sp. (based on morphological examination) were distinguished into three genera based on re-examination of their morphological features guided by their SIP identification: *Tedania* sp. (SAMA S1982, S1991, and S1994), *Desmapsamma* sp. (SAMA S1978), and *Chondropsis* sp. (SAMA S1984).

### SIP reliability evaluated by morphological identification

To mitigate the limitation of the incomplete gene database, the sponge identities inferred from any two of the DNA loci were taken into account to confirm an identity. The comparison with the re-examined morphological classification was to evaluate the reliability of the identification. Specifically, the sequence information of 18 sponges (Supplementary Table S3) with both the COI mtDNA and 28S rRNA gene loci were compared with the revised morphological identification at the genus, family, and order levels. There were better matches at the higher taxonomic rank with a maximum matching rate of 94% at the order level (Table 4). When the locus used to identify the sponges was compared with the morphological classification, the order level matching rate (%) remained the same (94%); however, the matching rates at the family and genus levels were noticeably higher. For sponges with at least two valid sequencing results, the three DNA markers supported each other as evidenced by the high matching rate at the order, family, and genus levels (91%, 73%, and 45%, respectively).

### Suitability and capacity of three DNA markers for sponge identification

The resolution of the three DNA loci to identify sponges was analysed by separately comparing the matching rate between the molecular information of each locus and the re-examined morphological classification for a given sponge species (Table 4). The molecular identification results for 19 out of the 20 valid 28S rRNA sequence pairs matched the morphological classification at the order level, nine at the family level, and six at the genus level. Similarly, the COI mtDNA and ITS sequence data were analysed using the 29 and 11 valid sequencing results, respectively. Of the 29 for COI loci and 11 ITS loci, 27 and 10, respectively, matched at the order level. The 28S locus showed the highest matching rate (%) among these three loci at the order and family levels. For the genus level, COI and 28S loci showed a similar performance but significantly better than the ITS locus. For these sponges with sequence similarities ≥98%, more sponge species can be inferred by their COI locus than their 28S locus at the order, family, and genus levels, which matched their morphological identity. However, the 28S locus had a slightly higher matching rate than the COI locus at all three taxonomic levels, possibly due to its lower number of the valid sequences. Notably, the ITS locus showed a significantly lower matching rate than the other two loci in all these cases.

### Limited entries in database

The limited number of the ITS submissions in the reference databases often resulted in a low Bit Score and sequence similarity as well as the potential for misidentification. To validate this hypothesis, we enumerated the total number of submissions in the NCBI database for COI mtDNA, 28S rRNA gene, and ITS belonging to the 10 orders of Porifera identified in this study (Supplementary Table S6). The number of submissions associated with the ITS region was the lowest of the three loci. A total of 964 submissions for the ITS locus included 367 sequences covering 26 different species from the order Clionaida, that has the largest contribution of the 10 orders. In contrast, the smallest contribution was for the order Tethyida with only 11 accessions.

For the sponge species belonging to the orders Haplosclerida (e.g. SAMA S1971), Poecilosclerida (e.g. SAMA S1982), and Tetractinellida (e.g. SAMA S1983), the COI mtDNA and 28S rRNA loci had a much higher Bit Score and sequence similarity (≥98%) than the ITS locus (Supplementary Table S3). Supplementary Table S7 shows some sponge species as examples in the orders Haplosclerida, Poecilosclerida, and Tetractinellida having the varying numbers of the submissions associated with the three DNA loci in the database.

## Discussion

Primer selection is a crucial part of PCR amplification to conduct the proposed SIP for sponge identification. Particularly for marine sponges, no single pair of primers could amplify the desired gene from all the sponge species used in this study (Supplementary Table S2 and Table 1). The quality of the sponge specimens was another essential factor for successful amplification. The degradation or contamination of the sponge specimens may result in a failure of gene amplification (Supplementary Table S4: SAMA S1975, S1977, S1978, S1979, and S1980). Consistent with the previous study^39^, our results indicate that identification using the ITS locus had much less value than the other loci. The ITS region has a higher evolutionary rate than the other loci^40^ and insufficient database entries. The advantage of the ITS locus is the variety in size due to indels which may be informative at some level. Consequently, using secondary structure to guide the alignment may also help in this situation.

The Sponge Identification Protocol (SIP) developed in this study was tested and evaluated with 111 individual sponges. The outcomes revealed the problems and challenges inherent in the current molecular identification process using a single molecular marker. The reliability of the developed SIP has been validated and well matched by phylogenetic analysis (Table 2 and Supplementary Figs S1 and S2). The character-based (Maximum Likelihood) phylogenetic trees demonstrated that the final inferences from SIP matched exactly with the identities inferred from both the 28S rRNA gene and COI mtDNA phylogenetic trees, if the databases for the two DNA loci had matching entries. Otherwise, the identity with the higher Bit Score always had a closer phylogenetic relationship with the query sequence based on the trees for the COI and 28S loci. In the distance-based (Neighbour Joining) phylogenetic trees, the results obtained were consistent. All of these analyses implied that the incomplete database entries not only limit the BLAST search but also restrict the application of phylogenetic analysis for sponge identification, unless a multi-locus approach such as SIP, developed in this study, can be utilised.

In the SIP, applying the normalised similarity score (Bit Score) as the identification indicator, in conjunction with multiple DNA markers, was proven to be a highly efficient and reliable sponge identification approach. The combination of the three loci not only offered a broader coverage of identifiable sponges (92%) but also improved the reliability when the identities from two or more loci matched each other. Compared to the identification performance of the single marker in SIP (COI mtDNA: 11; 28S rRNA: 19; ITS region: 4; refer to Supplementary Table S2), the multilocus approach substantially increased the identification efficacy (Table 1). Thirty four sponges were classified following the established SIP. Only three sponges failed to be identified due either to no PCR amplicon, substandard Coverage Region, or a non-Porifera sequence. More than 50% of sponges had a ≥98% sequence similarity. When the sequence similarity was ≥90%, 85% of the sponges could be classified. Without SIP, the BLAST results from any one of the three loci were: COI mtDNA: 29; 28S rRNA: 20; ITS region: 11 (refer to Supplementary Table S2). However, most of these results for the COI mtDNA (18 out of 29) and ITS region (7 out of 11) were unable to infer the sponge identity due to their lower similarities or lower sequence quality (Supplementary Table S3).

Mismatches between morphological and molecular identifications are common in sponges and fall across a spectrum of discordance^41^. Re-examination of the morphological features after the molecular identification is essential to achieve a more reliable identity when considering the limitations of both of the molecular and morphological approaches. On the one hand, the Genbank data are limited by the number and the diversity of entries. The reliability of the sequence data submitted to the database also greatly impacts the identification. On the other hand, there are many sponge taxa with little or no spicule diversity at all for species level discrimination^42^. Some families even showed a high degree of similarity in their spicules^42^. Particularly, for some local sponge species (e.g. South Australian sponges), it was difficult to identify them to the species level solely on their morphological features, without the accepted type species available. In our study, a 98% sequence similarity was selected as a threshold value as this level of confidence is required for a robust SIP identification. In conjunction with the morphological re-examination, the SIP offered a more reliable identification.

A multilocus approach has been employed by other researchers using different combinations with mixed results^11^,^33^,^43^,^44^. In addition, the amount of congruence observed between molecular and morphological data sets varied among different types of molecular data and sponge taxa^45^,^46^. In this study, when compared with morphological classification (Table 4), the molecular identification had a higher matching rate at the order level for both of these two categories: three element matching (COI mtDNA, 28 rRNA, and morphological classification) and two element matching (the locus used to infer the identity and morphological classification). Notably, the latter category increased at the family and genus levels. Additionally, the matching rates of the identities from any two DNA markers were much higher than the previous two categories at the family and genus levels (Table 4). Therefore, the reliability of the developed SIP (molecular loci only) was further validated, as the main principle of SIP is also to utilise one of the three loci with the highest similarity (Bit Score).

The multilocus-based BLAST protocol greatly improves the quality of inferences by coupling the accuracy present in different DNA markers. The resolution of the DNA markers was jointly demonstrated with the morphological classifications in three aspects: accuracy, reliability and suitability (Table 4). In our study, the nuclear 28S rRNA gene was found to have higher resolution than the mitochondrial COI gene or the nuclear ITS region. However, the COI locus is common for sponges as it offered the largest number of valid sequences. In some cases, such as the sponges in Poecilosclerida, the COI locus showed a better resolution at the order level (Supplementary Table S8).

Notably, limited database submissions can skew the identification accuracy regardless of the BLAST approach or the phylogenetic tree method. Caution should be exercised in assigning a new species or genus when the sequence similarity was lower than 98% using any one locus alone (Supplementary Table S6). The nucleotide database for ITS locus is not only limited by the small number of submissions, but also the lack of species diversity. For example, in the order Clionaida with the largest number of ITS submissions among the 10 sponge orders in this study, the numbers of accessions associated with COI, 28S, and ITS loci are 26, 379 and 367, respectively (Supplementary Tables S6). However, the ITS locus only covered 26 species compared to the 28S locus covering more than 170 species^37^. The application of multiple loci reflects the limitation of a single locus based approach and highlights the necessity to consider the diversity of the database entries when identifying sponges.

Overall, the results indicate that the application of multiple loci is essential for both of the SIP and phylogenetic approach to achieve a level of confidence for sponge identification due to the limited gene database entry of single DNA marker. The SIP, in which the various resolutions of three DNA markers complement each other, was completely validated by the phylogenetic analysis. SIP is more effective and practical than building different trees for every query sequence identification. Re-examination of the morphological identification guided by SIP identities leads to revised sponge identities, which reinforces the value of SIP. In conclusion, the multilocus-based SIP integrated with morphological identification offers an improved protocol that is effective for a more reliable sponge identification.

## Materials and Methods

### Sponge collection and morphological classification

**Figure 4:**
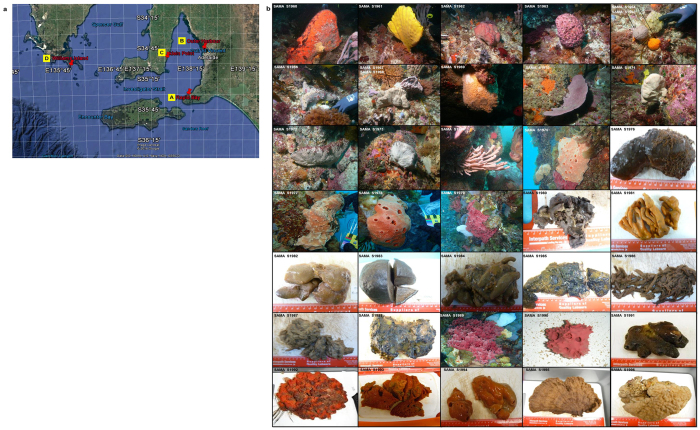
The sampling locations and the photos of the 37 sponge species. (**a**) Four sampling locations (A–D), Map data: Google Earth. (**b**) Photos of the 37 sponge species (underwater photos courtesy of David Wiltshire).

Sponges in this study were sampled under Exemption Permit Number 9902620 by the South Australian Research Development Institute (SARDI), issued by Primary Industries and Regions South Australia. Materials were used by Flinders University under a Material Transfer Agreement with SARDI and did not involve endangered or protected species. Sponges were collected from four different geographic locations in South Australia in 2012 and 2013 (Fig. 4a): Rapid Bay (35°31′17.25″S 138°11′15.26″E), Outer Harbour (34°46′26.90″S 138°29′08.54″E), Klein Point (34°55′41.79″S 137°47′19.42″E), and Williams Island (35°01′37.51″S 135°58′2.11″E). A portable fridge and iceboxes kept the sponge chilled during transportation.

The sample treatment was conducted immediately after the samples arrived at the laboratory. They were washed by sterilized sea water to remove soil or contamination. The photos were taken as records. The sponges were cut into small pieces (about 1 cm^3^) and were stored in sealed sample bags. They were kept in −80 °C for the following analysis and long term storage. Several pieces covering the surface and internal tissues were stored in a sample jar with 70% ethanol for morphological identification.

All the 37 potential species (111 sponge individuals) were first classified using morphological features^6^. Preparation for histological sections and spicule preparations followed the methods in ‘sponguide’^47^. The classification followed the revised Demosponge classification^48^. All the species are lodged with the South Australian Museum in Adelaide, South Australia with the museum vouchers SAMA S1960 to SAMA S1996. The photos of the 37 sponge species are presented in Fig. 4b. Brief descriptions of the morphological characteristics are available in the Supplementary Table S5.

### DNA extraction

Whole genomic DNA was extracted from sponge tissue frozen at −80 °C. A conventional hexadecyltrimethylammonium bromide (CTAB)-based protocol^49^ was used for isolating DNA. Briefly, the sponge tissues were ground under liquid nitrogen. The CTAB extraction buffer was applied to lyse tissues and then combined with polyvinylpyrrolidone (PVP) and β-mercaptoethanol to help remove phenolic compounds and tannins in the extract. To separate the proteins and polysaccharides from nucleic acids, phenol: chloroform: isoamyl alcohol (25:24:1) was utilised before DNA was precipitated with chilled isopropanol. DNeasy Blood & Tissue Kit (QIAGEN, Germany) was used for sponges that did not yield high quality DNA with the CTAB method. For each potential species, triplicate DNA extractions from three different individuals were obtained. The purified DNA was resuspended in 35 μl of sterile distilled water and stored at −20 °C. The purity and quantity of DNA were determined with a Nanodrop 1000 Spectrophotometer (Thermo Scientific, Wilmington, DE, USA) and only high quality DNA was used for subsequent PCR reactions.

### PCR amplification and sequencing

The COI locus was amplified using the universal primers LCO1490 (5′-GGT CAA CAA ATC ATA AAG ATA TTG G-3′) and HCO2198 (5′-TAA ACT TCA GGG TGA CCA AAA AAT CA-3′)^25^. The thermocycler was programmed as follows: a 1-min initial denaturation at 94 °C; 5 cycles of 94 °C for 30 sec, 45 °C for 90 sec and 72 °C for 1 min; 35 cycles of 94 °C for 30 sec, 51 °C for 40 sec and 72 °C for 1 min; and a final extension step at 72 °C for 5 min. For the sponges that could not be amplified using these universal primers, the following two pairs of universal metazoan primers were applied in a nested-PCR to amplify the target gene: C1-J2165 (5′-AAG TTT ATA TTT TAA TTT TAC CCC AGT GG-3′) and C-Npor 2760 (5′-TCT AGG TAA TCC AGC TAA ACC-3′)^50^; CO1porF1 (5′-CCN CAN TTN KCN GMN AAA AAA CA-3′) and CO1porR1 (5′-AAN TG N TGN GGR AAR AAN G-3′)^51^. For the second round of the nested-PCR, 5 μl of the amplicon adjusted to a DNA concentration of 50 ng/μl was used as the template.

One set of primers developed to amplify the partial 28S rRNA gene was NL4F (5′-GAC CCG AAA GAT GGT GAA CTA-3′) and NL4R (5′-ACC TTG GAG ACC TGA TGC G-3′)^11^ for regions D3- D5. The complete 28S rRNA gene alignment demonstrated that this region exhibited suitable levels of variability between sponge taxa allowing for the resolution of relatively deep phylogenetic relationships^52^. Thermocycler conditions were as follows: a 10-min initial denaturation at 95 °C; 35 cycles of 95 °C for 1 min, 56 °C for 1 min and 72 °C for 1 min; and a final extension step at 72 °C for 7 min. For the sponges that could not be amplified, alternative primers were employed: RD3A (5′-GAC CCG TCT TGA AAC ACG A-3′) and RD5B2 (5′-ACA CAC TCC TTA GCG GA-3′)^22^.

The ITS was amplified using the following thermocycler program: 94 °C for 2 min; 35 cycles of 94 °C for 30 s, 45 °C for 20 s, 65 °C for 60 s; and a final extension step of 72 °C for 10 min. The ITS primers were ITSRA2 (5′-GTC CCT GCC CTT TGT ACA CA-3′) and ITS2.2 (5′-CCT GGT TAG TTT CTT TTC CTC CGC-3′)^53^.

Duplicate PCRs of each locus were prepared for three individuals belonging to one potential species. The amplification products were purified using the Ultra Clean^®^ PCR Clean-Up Kit (MoBio), then Sanger-sequenced at the Institute of Microbiology, Chinese Academy of Sciences, Beijing, China.

### Data processing

The following protocol was developed to process the sequence data in order to work through the proposed SIP in this study (Fig. 1), including trimming the sequences (forward and reverse), checking the validity, phasing heterozygous sequences, as well as consensus generation and filtering.

Generally, the raw data of the forward and reverse sequences were trimmed individually by Sequencer 5.3^54^ under the setting: for the 5′ end, trimming no more than 25%, trim until the first 50 bases contain less than 1 ambiguity; and for the 3′ end, starting from 100 bases after the 5′ trim, trim the first 50 bases containing more than 1 ambiguity. The trimmed forward and reverse sequences were aligned separately against the sequences in NCBI Genbank database^37^. The sequences not belonging to Porifera and the ones with substandard lengths (<200 bp) were excluded.

The heterozygosity was checked to separate the sequence pairs from each sponge species into three groups: (1) the pairs are homozygous (45%); (2) the pairs with their heterozygotes presenting a single double peak in the chromatogram (30%); (3) the pairs with multiple length-variant heterozygotes (25%) on either one or both of the sequence strands. For the first two groups, the data can be edited easily following the protocol in Fig. 1. In group (3), the ambiguous bases were replaced with a mixed base symbol for nuclear marker phasing. The converted sequences were reconstructed to an optimal sequence of the corresponding two alleles^55^ before running the online Basic Local Alignment Search Tool (BLAST) searches. The reconstruction was accomplished by a web tool (Champuru) available online at http://jfflot.mnhn.fr/champuru/ that automates the process^56^. The assembled consensus sequences derived from every sponge species were checked directly using BLAST searches^57^ against the NCBI GenBank database^37^. Additionally, the results from the BLAST search, when consisted of less than 50% Coverage Region, were considered unreliable and excluded. In other words, the participating part of the sequence needs to represent more than half of the query.

### Development of a Sponge Identification Protocol (SIP)

The proposed Sponge Identification Protocol (SIP) is shown in Fig. 2. The molecular identities of each potential sponge species were inferred by SIP. The application of the multiple-locus strategy in SIP was an attempt to mitigate the effects of database limitations existing for single DNA marker based protocols for reliable sponge identification. Instead of building a phylogenetic tree separately, the three identities were referred to each other to infer the classification, as using any single DNA locus is restricted by its limited number of database submissions, resulting in inaccurate identifications. However, these limitations could not be fully avoided, due to unreliable morphologically identified submissions in the database.

Reliable inferences require reliable statistical estimates. The Bit Score is a prominent statistical indicator used in addition to the E-value in a BLAST output^58^. As a raw similarity score, Bit Score and E-value reflect the evolutionary distance of the two aligned sequences, the length of the sequences, and the scoring matrix used for the alignment. A Bit Score is normalized with respect to the scoring system and can be used to compare alignment scores from different searches. The higher the Bit Score, the more highly significant the match is. In contrast, sequence similarity (%) is not as sensitive or reliable. It is a useful approximation for analyses that depend on evolutionary distance, but evolutionary distance is not linear with percent similarity. The evolutionary distance associated with a 10% change in similarity is much greater at longer distances. Here, the Bit Score and E-value are far more useful. However, the E-values showed 0.0 for most cases, which rules it out as a comparative indicator. Therefore, the Bit Score was selected as the priority identification parameter for sponges in this newly proposed SIP: (1) The one with the highest Bit Score in the BLAST result list was selected as the identity for every single DNA locus. It is important to consider the top 20 BLAST results in the list for any possible errors, otherwise the one with highest Bit Score accompanied with the best E-value, sequence similarity, and percentage coverage was selected. (2) The one with the highest Bit Score among the three loci was chosen to be the final SIP identity. All of the consensus sequences used for providing the identities of different individuals were submitted into NCBI GenBank under the accession numbers KJ546351-546362, KJ546354-546368, KJ620376-620395, KJ620398-620409, KJ782592, KJ782595, KJ782600, KJ782602, KJ782604, KJ801654, KJ801656 and KJ801658-801661.

### Phylogenetic analysis

The valid consensus for each of the query sequence were submitted to the NCBI database to be searched by online Basic Local Alignment Search Tool (BLAST)^57^. The top 20 sequences were downloaded as a FASTA format file. The outgroup was chosen based on the general principle that it has a close enough relationship with the in-group but is not part of the in-group. Considering many genera of sponges are not monophyletic, representative sequences belonging to a few genera were chosen to be the outgroup. Software BioEdit^59^ was applied to combine the query sequence and the references in the FASTA file obtained from the previous BLAST search. Software MEGA6^60^ was used to align the sequences using ClustalW algorithm, and trim the aligned sequences to make them ready for tree construction. The alignment was exported as a MEGA format file. The Maximum Likelihood (ML) and Neighbor Joining (NJ) methods were utilized to construct the phylogenetic trees for COI mtDNA and 28S rRNA gene separately. Both of the methods were set with 1000 of bootstrap replications. ML trees used Tamura-Nei model and NJ trees applied p-distance model. The best-fit of substitution models for the Maximum Likelihood method was evaluated by facilitating the analysis of “Find Best DNA/Protein Models (ML)” in the menu “Models” of MEGA6^60^. “Automatic (Neighbor-joining tree)”^61^ was set as the choice of tree to use and “Partial deletion” with “95% Site Coverage Cutoff” was selected as the method to treat missing data and alignment gaps. The model with the lowest Bayesian information criterion (BIC) score was considered to describe the substitution pattern the best.

### Morphological re-examination and final identification

Guided by the molecular identification of SIP (the discrepancy between the SIP and initial morphological identities), a re-examination of the morphological features was conducted. The re-examination followed the protocol mentioned in section Sponge collection and morphological classification. The rules applied in this study were (1) if the re-examined morphological identity matched with the SIP identity at genus level, the SIP identity was chosen to the final identity; otherwise, (2) to check the morphological features of these sponges matched at the order or family level, if the morphological features are highly similar and difficult to discriminate, their SIP identity was selected as the final; (3) if they are not morphologically similar, a threshold of 98% for the sequence similarity was utilised to determine the final identity. The SIP identity with ≥98% sequence similarity against the database entries was selected as the final identity, the others were assigned the final identity based on the morphological identification.

## Additional Information

**How to cite this article:** Yang, Q. *et al*. Development of a multilocus-based approach for sponge (phylum Porifera) identification: refinement and limitations. *Sci. Rep.* **7**, 41422; doi: 10.1038/srep41422 (2017).

**Publisher's note:** Springer Nature remains neutral with regard to jurisdictional claims in published maps and institutional affiliations.

## Supplementary Material

Supplementary Information

## Figures and Tables

**Table 1:** Summary of molecular identification of thirty-four sponges using three DNA markers and following the sponge order in Supplementary Table S1.

Museum Voucher	Identification Result (Order; Family; Genus/Species) (ID locus, Bit score, sequence similarity, coverage %)	Accession No.
**SAMA S1991**	Poecilosclerida; Tedaniidae; *Tedania tubulifera* (28S, 992, 99%, 97%)	KJ620377
**SAMA S1994**	Poecilosclerida; Tedaniidae; *Tedania tubulifera* (28S, 1000, 99%, 96%)	KJ620378
**SAMA S1982**	Poecilosclerida; Tedaniidae; *Tedania tubulifera* (28S, 979, 98%, 98%)	KJ620381
**SAMA S1984**	Poecilosclerida; Tedaniidae; *Tedania tubulifera* (28S, 870, 95%, 92%)	KJ620384
**SAMA S1978**	Poecilosclerida; Desmacididae; *Desmapsamma anchorata* (COI, 581, 97%, 93%)	KJ546367
**SAMA S1987**	Poecilosclerida; Mycalidae; *Mycale setosa* (28S, 305, 84%, 55%)	KJ620385
**SAMA S1966**	Poecilosclerida; Mycalidae; *Mycale setosa* (28S, 1008, 99%, 85%)	KJ620392
**SAMA S1975**	Poecilosclerida; Hymedesmiidae; *Phorbas bihamiger* (COI, 469, 91%, 95%)	KJ546364
**SAMA S1977**	Poecilosclerida; Hymedesmiidae; *Phorbas bihamiger* (COI, 453, 91%, 90%)	KJ546366
**SAMA S1993**	Poecilosclerida; Desmacididae; *Desmapsamma anchorata* (COI, 614, 98%, 97%)	KJ546354
**SAMA S1969**	Dictyoceratida; Irciniidae; *Ircinia felix* f. *felix* (ITS, 358, 87%, 70%)	KJ801659
**SAMA S1976**	Suberitida; Halichondriidae; *Halichondria okadai* (ITS, 617, 94%, 99%)	KJ801656
**SAMA S1962**	Tetractinellida; Ancorinidae; *Ecionemia robusta* (COI, 1010, 99%, 99%)	KJ620388
**SAMA S1983**	Tetractinellida; Ancorinidae; *Tethyopsis mortenseni* (28S, 967, 98%, 97%)	KJ620383
**SAMA S1963**	Tetractinellida; Ancorinidae; *Stelletta clavosa* (28S, 1023, 99%, 97%)	KJ620389
**SAMA S1968**	Poecilosclerida; Microcionina; *Clathria rugosa* (COI, 510, 93%, 94%)	KJ620406
**SAMA S1996**	Dictyoceratida; Irciniidae; *Ircinia strobilina* (28S, 845, 94%, 94%)	KJ620380
**SAMA S1974**	Dictyoceratida; Irciniidae; *Ircinia felix* f. *felix* (ITS, 398, 83%, 100%)	KJ801661
**SAMA S1979**	Dendroceratida; Dictyodendrillidae; *Acanthodendrilla australis* (COI, 526, 97%, 84%)	KJ546368
**SAMA S1970**	Dictyoceratida; Irciniidae; *Ircinia felix* f. *felix* (ITS, 349, 81%, 99%)	KJ801660
**SAMA S1995**	Axinellida; Raspailiidae; *Raspailia vestigifera* (28S, 822, 96%, 82%)	KJ620379
**SAMA S1967**	Haplosclerida; Petrosiidae; *Petrosia lignosa* (28S, 826, 93%, 96%)	KJ620393
**SAMA S1972**	Axinellida; Raspailiidae; *Echinodictyum cancellatum* (COI, 467, 93%, 85%)	KJ620408
**SAMA S1973**	Verongiida; Aplysinidae; *Aplysina archeri* (28S, 1005, 98%, 97%)	KJ620395
**SAMA S1985**	Verongiida; Pseudoceratinidae; *Pseudoceratina* sp. (COI, 634, 99%, 95%)	KJ546361
**SAMA S1988**	Verongiida; Pseudoceratinidae; *Pseudoceratina* sp. (COI, 619, 99%, 93%)	KJ546363
**SAMA S1971**	Haplosclerida; Chalinidae; *Cladocroce* sp. (28S, 1085, 99%, 98%)	KJ620394
**SAMA S1980**	Suberitida; Suberitidae; ‘*Protosuberites*’ sp. (COI, 404, 87%, 96%)	KJ620398
**SAMA S1960**	Clionaida; Spirastrellidae; *Spirastrella hartmani* (28S, 1012, 99%, 98%)	KJ620386
**SAMA S1961**	Poecilosclerida; Podospongiidae; *Diacarnus spinipoculum* (28S, 953, 99%, 92%)	KJ620387
**SAMA S1989**	Dendroceratida; Dictyodendrillidae; *Igernella notabilis* (28S, 977, 98%, 98%)	KJ620376
**SAMA S1965**	Suberitida; Halichondriidae; *Hymeniacidon heliophila* (28S, 1004, 99%, 96%)	KJ620391
**SAMA S1981**	Suberitida; Suberitidae; *Suberites aurantiacus* (28S, 975, 98%, 97%)	KJ620381
**SAMA S1964**	Tethyida; Tethyidae; *Tethya* sp. (28S, 973, 98%, 98%)	KJ620390

**Table 2:** Reliability of SIP validated by phylogenetic analysis.

	BLAST result		Inference from SIP	Phylogenetic analysis- Maximum Likelihood		Phylogenetic analysis- Neighbor-Joining	
	COI identity (Bit Score, % similarity)	28S identity (Bit Score, % similarity)		COI locus	28S locus	COI locus	28S locus
**SAMA S1981**	*Rhizaxinella* sp. (608, 98%)	*Suberites aurantiacus* (975, 98%)	*Suberites aurantiacus*	*Suberites aurantiacus*	*Suberites aurantiacus*	*Suberites aurantiacus*	*Suberites aurantiacus*
**SAMA S1963**	*Ecionemia* sp. (641, 99%)	*Stelletta clavosa* (1023, 99%)	*Stelletta clavosa*	*Stelletta clavosa*	*Stelletta clavosa*	*Stelletta clavosa*	*Stelletta clavosa*
**SAMA S1965**	*Protosuberites* sp. (598, 97%)	*Hymeniacidon heliophila* (1004, 99%)	*Hymeniacidon heliophila*	*H. perlevis; H. helliophila; H. sinapium*	*Hymeniacidon heliophila*	*H. perlevis; H. helliophila; H. sinapium*	*Hymeniacidon heliophila*
**SAMA S1973**	*Aplysina lacunose* (611, 98%)	*Aplysina archeri* (1005, 98%)	*Aplysina archeri*	*Aplysina archeri*	*Aplysina archeri*	*Aplysina archeri*	*Aplysina archeri*
**SAMA S1989**	*Igernella notabilis* (629, 98%)	*Igernella notabilis* (977, 98%)	*Igernella notabilis*	*Igernella notabilis*	*Igernella notabilis*	*Igernella notabilis*	*Igernella notabilis*
**SAMA S1961**	*Diacarnus spinipoculum* (657, 99%)	*Diacarnus spinipoculum* (953, 99%)	*Diacarnus spinipoculum*	*Diacarnus spinipoculum*	*Diacarnus spinipoculum*	*Diacarnus spinipoculum*	*Diacarnus spinipoculum*
**SAMA S1962**	*Ecionemia robusta* (1010, 99%)	*Stelletta clavosa* (642, 99%)	*Ecionemia robusta*	*Ecionemia robusta*	*Ecionemia acervus*	*Ecionemia robusta*	*Ecionemia acervus*
**SAMA S1991**	*Desmapsamma anchorata* (612, 98%)	*Tedania tubulifera* (992, 99%)	*Tedania tubulifera*	*T. ignis; T. klausi*	*Tedania tubulifera*	*T. ignis; T. klausi*	*Tedania tubulifera*
**SAMA S1982**	*Desmapsamma anchorata* (615, 99%)	*Tedania tubulifera* (979, 98%)	*Tedania tubulifera*	*T. ignis; T. klausi*	*Tedania tubulifera*	*T. ignis; T. klausi*	*Tedania tubulifera*
**SAMA S1994**	*Tedania ignis* (604, 98%)	*Tedania tubulifera* (1000, 99%)	*Tedania tubulifera*	*Tedania ignis*	*Tedania tubulifera*	*Tedania ignis*	*Tedania tubulifera*
**SAMA S1966**	*Mycale mirabilis* (633, 99%)	*Mycale setosa* (1008, 99%)	*Mycale setosa*	*Mycale mirabilis*	*Mycale setosa*	*Mycale mirabilis*	*Mycale setosa*
**SAMA S1960**	*Clionaopsis platei* (605, 97%)	*Spirastrella hartmani* (1012, 99%)	*Spirastrella hartmani*	*Cliona chilensis*	*Spirastrella hartmani*	*Cliona chilensis*	*Spirastrella hartmani*
**SAMA S1971**	*Callyspongia siphonella* (581, 98%)	*Cladocroce* sp. (1085, 99%)	*Cladocroce* sp.	*Callyspongia siphonella*	*Cladocroce* sp.	*Callyspongia siphonella*	*Cladocroce* sp.
**SAMA S1983**	*Ancorina* sp. (600, 98%)	*Tethyopsis mortenseni* (967, 98%)	*Tethyopsis mortenseni*	*Pleroma menoui*	*Tethyopsis mortenseni*	*Pleroma menoui*	*Tethyopsis mortenseni*

**Table 3:** Re-examination of morphological classification and comparison with SIP identification.

Museum Voucher	Initial morphological classification	Re-examination of morphological classification	SIP identification	Notes on difference in IDs between morphology and molecular
**Category I: Genus level match between morphology and SIP**				
**SAMA S1962**	*Ecionemia* sp.	*Ecionemia* sp.	*Ecionemia robusta* (1010, 99%, 99%)	*E. robusta* is now accepted as *Ancorina robusta*, occurs in South Australia. This is possibly a species match.
**SAMA S1964**	*Tethya* cf. *bergquistae*	*Tethya* cf. *bergquistae*	*Tethya* sp. (973, 98%, 98%)	
**SAMA S1966**	*Mycale (Arenochalina*) sp.	*Mycale (Arenochalina*) sp.	*Mycale setosa* (1008, 99%, 85%)	*M. setosa* is a Red Sea sponge.
**SAMA S1972**	*Echinodictyum mesenterinum*	*Echinodictyum mesenterinum*	*Echinodictyum cancellatum* (467, 93%, 85%)	Both E. *mesenterinum. and E. cancellatum* occur in South Australia (SA), but their morphology is distinct.
**SAMA S1973**	*Aplysina lendenfeldi*	*Aplysina lendenfeldi*	*Aplysina archeri* (1005, 98%, 97%)	*A. archeri* (Higgin, 1875) is a Caribbean yellow tubular sponge; it is very similar in appearance to Australia’s *A. lendenfeldi* (Bergquist, 1980) which is also a yellow tubular sponge.
**SAMA S1974**	*Ircinia* sp.	*Ircinia* sp.	*Ircinia felix* f. *felix* (398, 83%, 100%)	*I. felix* is a Caribbean/Brazilian sponge.
**SAMA S1981**	*Suberites* sp.	*Suberites* sp.	*Suberites aurantiacus* (975, 98%, 97%)	*S. aurantiacus* is a Caribbean sponge.
**SAMA S1987**	*Mycale (Zygomycale*) sp.	*Mycale (Zygomycale*) sp.	*Mycale setosa* (305, 84%, 55%)	*M. setosa* is a Red Sea sponge.
**SAMA S1996**	*Ircinia* sp.	*Ircinia* sp.	*Ircinia strobilina* (845, 94%, 94%)	*I. stobilina* is a Caribbean/Brazilian sponge.
**Category II: Order/family level match between morphology and SIP; highly similar each other**				
**SAMA S1960**	*Cliona* sp.	*Cliona* sp.	*Spirastrella hartmani* (1012, 99%, 98%)	*S. hartmani* (Boury-Esnault, Klautau, Bézac, Wulff & Solé-Cava, 1999) is a Caribbean/Brazillian sponge. The families containing *Cliona* (Clionaidae) and *Spirastrella* (Spirastrellidae) are close families, with historically some genera moving from one to another^6^.
**SAMA S1971**	*Callyspongia (Callyspongia*) sp.	*Callyspongia (Callyspongia*) sp.	*Cladocroce* sp. (1085, 99%, 98%)	*Callyspongia* (Callyspongiidae) and *Cladocroce* (Chalinidae) are in different families.
**SAMA S1975**	*Crella* sp. 1	*Crella* sp. 1	*Phorbas bihamiger* (469, 91%, 95%)	*P. bihamiger* (Waller, 1878) is a green encrusting UK/North Atlantic sponge. The families containing *Crella* (Crellidae) and *Phorbas* (Hymedesmiidae) are very close differing in only the surface arrangement of spicules^6^.
**SAMA S1977**	*Crella* sp. 1	*Crella* sp. 1	*Phorbas bihamiger* (453, 91%, 90%)	As above.
**SAMA S1978**	*Chondropsis* sp.	*Chondropsis* sp.	*Desmapsamma anchorata* (581, 97%, 93%)	*Desmapsamma* (Desmacididae) and *Chondropsis* (Chondropsidae) are both Poecilosclerid. sand-bearing sponges, but in different families.
**SAMA S1989**	*Aplysilla rosea*	*Aplysilla rosea*	*Igernella notabilis* (977, 98%, 98%)	*I. notabilis* (Duchassaing & Michelotti, 1864) is a fleshy pink conulose Caribbean sponge. *A. rosea* (Barrois, 1876) is a fleshy pink conulose sponge from NE Atlantic region and the Mediterranean area. van Soest *et al*.^5^ state that other records are inaccurate, although *A. rosea* is listed in Australia^38^ and commonly cited.
**SAMA S1995**	*Echinodictyum mesenterinum*	*Echinodictyum mesenterinum*	*Raspailia vestigifera* (822, 96%, 82%)	*E. mesenterinum* and *R. vestigifera* share the same family.
**Category III: Order level match between morphology and SIP**				
**SAMA S1963**	Ancorinid sp.	*Ancorinid sp.*	*Stelletta clavosa* (1023, 99%, 97%)	*S. clavosa* does occur in SA.
**SAMA S1965**	*Caulospongia* sp.	*Caulospongia* sp.	*Hymeniacidon heliophila* (1004, 99%, 96%)	
**SAMA S1982**	*Chondropsis* sp.	*Chondropsis* sp.	*Tedania tubulifera* (979, 98%, 98%)	
**SAMA S1983**	Geodiid sp.	*Geodiid sp.*	*Tethyopsis mortenseni* (967, 98%, 97%)	
**SAMA S1984**	*Chondropsis* sp.	*Chondropsis* sp.	*Tedania tubulifera* (870, 95%, 92%)	
**SAMA S1991**	*Chondropsis* sp.	*Chondropsis* sp.	*Tedania tubulifera* (992, 99%, 97%)	
**SAMA S1992**	*Tedania* cf. *anhelans*	*Tedania* cf. *anhelans*	*Desmapsamma anchorata* (614, 98%, 97%)	
**SAMA S1994**	*Chondropsis* sp.	*Chondropsis* sp.	*Tedania tubulifera* (1000, 99%, 96%)	
**Category IV: Not match at order level**				
**SAMA S1961**	*Spheciospongia* sp.	*Spheciospongia* sp.	*Diacarnus spinipoculum* (953, 99%, 92%)	*D. spinipoculum* does occur in SA.
**SAMA S1968**	Astrophorin sp.	*Astrophorin sp.*	*Clathria rugosa* (510, 93%, 94%)	Possible contamination by an encrusting sponge.
**SAMA S1980**	*Haliclona* sp.	*Haliclona* sp.	'*Protosuberites*’ sp. (404, 87%, 96%)	
**Category V: Incorrect initial morphological classification or classification refined further on re-examination**				
**SAMA S1985**	Aplysinellid sp.	*Pseudoceratina* sp.	*Pseudoceratina* sp. (634, 99%, 95%)	Updated ID match at genus level
**SAMA S1988**	Verongid sp.	*Pseudoceratina* sp.	*Pseudoceratina* sp. (619, 99%, 93%)	Updated ID match at genus level
**SAMA S1979**	*Euryspongia* cf. *arenaria*	*Acanthodendrilla* sp.	*Acanthodendrilla australis* (526, 97%, 84%)	Updated ID match at genus level
**SAMA S1967**	*Echinodictyum mesenterinum*	*Callyspongia bilamellata*	*Petrosia lignosa* (826, 93%, 96%)	Updated ID match at order level
**SAMA S1969**	*Clathria* sp.	*Spongiid sp.*	*Ircinia felix* f. *felix* (358, 87%, 70%)	Updated ID match at order level
**SAMA S1970**	*Thorectandra* sp.	*Thorectid sp.*	*Ircinia felix* f. *felix* (349, 81%, 99%)	Updated ID match at order level
**SAMA S1976**	*Ecionemia* sp.	*Chondrosia* sp.	*Halichondria okadai* (617, 94%, 99%)	

**Table 4:** Comparison of molecular identification by the selected DNA marker(s) with or without morphological classification.

	COI and 28S Loci & Morphological classification			One locus used for inferring identity & Morphological classification			Any two loci matching each other		
	Genus match	Family match	Order match	Genus match	Family match	Order match	Genus match	Family match	Order match
Number of sponges	3	6	17	6	8	17	10	16	20
Percentage	3/18	6/18	17/18	6/18	8/18	17/18	10/22	16/22	20/22
%	17%	33%	94%	33%	44%	94%	45%	73%	91%
**Single locus & morphological classification**									
	ITS: 11 valid sequences			COI: 29 valid sequences			28S: 20 valid sequences		
Number of sponges	2	3	10	9	13	27	6	9	19
Percentage	2/11	3/11	10/11	9/29	13/29	27/29	6/20	9/20	19/20
%	18%	27%	90%	31%	44%	93%	30%	45%	95%
**Identity sequence similarity ≥98%**									
	ITS			COI			28S		
Number of sponges	0	0	0	6	8	17	4	6	14
Percentage	—	—	—	6/9	8/13	17/27	4/6	6/9	14/18
%	0%	0&	0%	66%	61%	62%	67%	67%	78%
