# Supplementary material for: Development of a multilocus-based approach for sponge (phylum Porifera) identification: refinement and limitations
Source: Sci Rep. 2017 Feb 2;7:41422. doi: 10.1038/srep41422 (PMC5288722; doi:10.1038/srep41422)
Supplement: Supplementary Information [file srep41422-s1.pdf]

Development of a multilocus-based approach for sponge (phylum Porifera) identification: refinement and limitations

Qi Yang[1, 2], Christopher M. M. Franco[1, 2*], Shirley J. Sorokin[1, 2, 3], Wei Zhang[1, 2, 4*]

[1] Centre for Marine Bioproducts Development, Flinders University, Adelaide, South Australia, SA 5042, Australia

[2] Department of Medical Biotechnology, School of Medicine, Faculty of Medicine, Nursing and Health Sciences, Flinders University, Adelaide, South Australia, SA 5042, Australia

[3] SARDI Aquatic Sciences, 2 Hamra Ave, West Beach, SA 5024, Australia

[4] Centre for Marine Drugs, Renji Hospital, Shanghai Jiaotong University, Shanghai, China, 200240

* Corresponding authors: Chris M. M. Franco and Wei Zhang

E-mail: chris.franco@flinders.edu.au (C.M.M.F.); wei.zhang@flinders.edu.au (W.Z)

Supplementary Information

Supplementary Table S1. Initial morphological classification of thirty-seven sponges including sampling locations and collection dates

Museum Voucher	Order	Genus/ Species	Location	Date
**SAMA S1991**	Poecilosclerida	*Chondropsis* sp.	Rapid Bay	25/02/13
**SAMA S1994**		*Chondropsis* sp.	Rapid Bay	25/02/13
**SAMA S1982**		*Chondropsis* sp.	Outer Harbour	01/03/13
**SAMA S1984**		*Chondropsis* sp.	Outer Harbour	01/03/13
**SAMA S1978**		*Chondropsis* sp.	Klein Point	04/03/13
**SAMA S1987**		*Mycale* (*Zygomycale*) sp.	Outer Harbour	01/03/13
**SAMA S1966**		*Mycale* (*Arenochalina*) sp.	Williams Island	19/05/13
**SAMA S1975**		*Crella* sp. 1	Klein Point	04/03/13
**SAMA S1977**		*Crella* sp. 1	Klein Point	04/03/13
**SAMA S1992**		Poecilosclerid sp.	Rapid Bay	25/02/13
**SAMA S1993**		*Tedania* cf. *anhelans*	Rapid Bay	25/02/13
**SAMA S1969**		*Clathria* sp.	Williams Island	19/05/13
**SAMA S1976**	Tetractinellida	*Ecionemia* sp.	Klein Point	04/03/13
**SAMA S1962**		*Ecionemia* sp.	Williams Island	19/05/13
**SAMA S1983**		Geodiid sp.	Outer Harbour	01/03/13
**SAMA S1963**		Ancorinid sp.	Williams Island	19/05/13
**SAMA S1968**		Astrophorin sp.	Williams Island	19/05/13
**SAMA S1996**	Dictyoceratida	*Ircinia* sp.	Rapid Bay	25/02/13
**SAMA S1974**		*Ircinia* sp.	Williams Island	19/05/13
**SAMA S1979**		*Euryspongia* cf. *arenaria*	Klein Point	04/03/13
**SAMA S1970**		*Thorectandra* sp.	Williams Island	19/05/13
**SAMA S1995**	Axinellida	*Echinodictyum mesenterinum*	Rapid Bay	25/02/13
**SAMA S1967**		*Echinodictyum mesenterinum*	Williams Island	19/05/13
**SAMA S1972**		*Echinodictyum mesenterinum*	Williams Island	19/05/13
**SAMA S1973**	Verongiida	*Aplysina lendenfeldi*	Williams Island	19/05/13
**SAMA S1985**		Aplysinellid sp.	Outer Harbour	01/03/13
**SAMA S1988**		Verongiid sp.	Outer Harbour	01/03/13
**SAMA S1971**	Haplosclerida	*Callyspongia* (*Callyspongia*) sp.	Williams Island	19/05/13
**SAMA S1986**		*Chalinula* sp.	Outer Harbour	01/03/13
**SAMA S1980**		*Haliclona* sp.	Klein Point	04/03/13
**SAMA S1960**	Clionaida	*Cliona* sp.	Williams Island	19/05/13
**SAMA S1961**		*Spheciospongia* sp.	Williams Island	19/05/13
**SAMA S1989**	Dendroceratida	*Aplysilla rosea*	Rapid Bay	27/06/12
**SAMA S1990**		*Aplysilla rosea*	Rapid Bay	22/08/12
**SAMA S1965**	Suberitida	*Caulospongia* sp.	Williams Island	19/05/13
**SAMA S1981**		*Suberites* sp.	Outer Harbour	01/03/13
**SAMA S1964**	Tethyida	*Tethya* cf. *bergquistae*	Williams Island	19/05/13

Supplementary Table S2. **Summary of molecular identification of the thirty-seven potential sponge species using multilocus approach**

		COI mtDNA	Success rate	28S rRNA gene	Success rate	ITS region	Success rate
**Data processing**	**DNA preparation**	37	100%	37	100%	37	100%
	**PCR products**	31	84%	25	68%	32	86%
	**Sequencing results**	31	100%	22	88%	27	84%
	**Valid results**	29	94%	20	91%	12	44%
	**Belong to Porifera**	29	100%	20	100%	11	92%
**SIP**	**Putative identification**	11	-	19	-	4	-
	**Putative different species**	8	-	15	-	2	-

Supplementary Table S3. DNA loci sequencing results with Bit Score, sequence similarity, coverage percentage and the morphological classifications of thirty-seven sponges. No. 3-1 ~ 3-5 are the sponges with three valid loci sequence information; No. 2-1 ~ 2-17are the sponges with two loci sequence information; No. 1-1 ~ 1-12 are the sponges with only one locus information; No. 0-1 ~ 0-3 are the sponges without inferences according to the Sponge Identification Protocol (SIP) in this study; ∨ indicates the one inferring the final identity; Underline labels the excluded data with substandard coverage region (<50%).

No.	Museum Voucher	COI Identities				28S Identities				ITS Identities				Morphological Classification (Order-Family-Genus/Species)
		PCR	Seq	Accession No.	Result (Order-Family-Genus/Species)	PCR	Seq	Accession No.	Result (Order-Family-Genus/Species)	PCR	Seq	Accession No.	Result (Order-Family-Genus/Species)	
3-1	SAMA S1981	∨	∨	KJ546357	Suberitida;<br>Suberitidae;<br>*Rhizaxinella* sp.<br>(608, 98%, 96%)	∨	∨	KJ620381	Suberitida;<br>Suberitidae;<br>*Suberites aurantiacus*<br>(975 ∨, 98%, 97%)	∨	∨	KJ782592	Suberitida;<br>Halichondriidae;<br>*Hymeniacidon heliophila*<br>(274, 84%, 62%)	Suberitida;<br>Suberitidae;<br>*Suberites* sp.
3-2	SAMA S1960	∨	∨	KJ620399	Clionaida;<br>Clionaidae;<br>*Clionaopsis platei*<br>(605, 97%, 96%)	∨	∨	KJ620386	Clionaida;<br>Spirastrellidae;<br>*Spirastrella hartmani*<br>(1012 ∨, 99%, 98%)	∨	∨	KJ782595	Clionaida;<br>Spirastrellidae;<br>*Spirastrella hartmani*<br>(480, 88%, 99%)	Clionaida;<br>Clionaidae;<br>*Cliona* sp.
3-3	SAMA S1965	∨	∨	KJ620404	Suberitida;<br>Suberitidae;<br>'*Protosuberites*' sp.<br>(598, 97%, 97%)	∨	∨	KJ620391	Suberitida;<br>Halichondriidae;<br>*Hymeniacidon heliophila*<br>(1004 ∨, 99%, 96%)	∨	∨	KJ782600	Suberitida;<br>Halichondriidae;<br>*Hymeniacidon heliophila*<br>(388, 84%, 98%)	Suberitida;<br>Suberitidae;<br>*Caulospongia* sp.
3-4	SAMA S1971	∨	∨	KJ620407	Haplosclerida;<br>Callyspongiidae;<br>*Callyspongia siphonella*<br>(581, 98%, 91%)	∨	∨	KJ620394	Haplosclerida;<br>Chalinidae;<br>*Cladocroce* sp.<br>(1085 ∨, 99%, 98%)	∨	∨	KJ782602	Haplosclerida;<br>Chalinidae;<br>*Haliclona* sp.<br>(183, 80%, 55%)	Haplosclerida;<br>Callyspongiidae;<br>*Callyspongia* sp.
3-5	SAMA S1973	∨	∨	KJ620409	Verongiida;<br>Aplysinidae;<br>*Aplysina lacunose*<br>(611, 98%, 96%)	∨	∨	KJ620395	Verongiida;<br>Aplysinidae;<br>*Aplysina archeri*<br>(1005 ∨, 98%, 97%)	∨	∨	KJ782604	Verongiida;<br>Aplysinidae;<br>*Verongula gigantean*<br>(335, 90%, 81%)	Verongiida;<br>Aplysinidae;<br>*Aplysina lendenfeldi*
2-1	SAMA S1989	∨	∨	KJ546351	Dendroceratida;<br>Dictyodendrillidae;<br>*Igernella notabilis*<br>(629, 98%, 98%)	∨	∨	KJ620376	Dendroceratida;<br>Dictyodendrillidae;<br>*Igernella notabilis*<br>(977 ∨, 98%, 98%)	∨	×		CANCEL<br>(The other two are all valid and matched at the species level.)	Dendroceratida;<br>Darwinellidae;<br>*Aplysilla rosea*
2-2	SAMA S1991	∨	∨	KJ546352	Poecilosclerida;<br>Desmacididae;<br>*Desmapsamma anchorata*<br>(612, 98%, 97%)	∨	∨	KJ620377	Poecilosclerida;<br>Tedaniidae;<br>*Tedania tubulifera*<br>(992 ∨, 99%, 97%)	∨	∨	-	Polymastiida;<br>Polymastiidae;<br>*Polymastia pachymastia*<br>(34, 98%, 3%)	Poecilosclerida;<br>Chondropsidae;<br>*Chondropsis* sp.
2-3	SAMA S1993	∨	∨	KJ546354	Poecilosclerida;<br>Desmacididae;<br>*Desmapsamma anchorata*<br>(614 ∨, 98%, 97%)	×	×		Try the second pair of primers (Positive)	∨	∨		Not sponge	Poecilosclerida;<br>Tedaniidae;<br>*Tedania* cf. *anhelans*

2-4	SAMA<br>S1994	∨	∨	KJ546355	Poecilosclerida;<br>Tedaniidae;<br>*Tedania ignis*<br>(604, 98%, 95%)	∨	∨	KJ620378	Poecilosclerida;<br>Tedaniidae;<br>*Tedania tubulifera*<br>(1000 ∨, 99%, 96%)	×	×		CANCEL<br>(The other two are all valid and matched at the genus level.)	Poecilosclerida;<br>Chondropsidae;<br>*Chondropsis* sp.
2-5	SAMA<br>S1995	∨	∨	KJ546356	Axinellida;<br>Raspailiidae;<br>*Echinodictyum cancellatum*<br>(470, 93%, 86%)	∨	∨	KJ620379	Axinellida;<br>Raspailiidae;<br>*Raspailia vestigifera*<br>(822 ∨, 96%, 82%)	∨	×		CANCEL<br>(The other two are all valid and matched at the family level.)	Axinellida;<br>Raspailiidae;<br>*Echinodictyum mesenterium*
2-6	SAMA<br>S1996	×	×		CANCEL<br>(The other two are all valid and matched at the genus level.)	∨	∨	KJ620380	Dictyoceratida;<br>Irciniidae;<br>*Ircinia strobilina*<br>(845 ∨, 94%, 94%)	∨	∨	KJ801654	Dictyoceratida;<br>Irciniidae;<br>*Ircinia felix* f. *felix*<br>(595, 90%, 96%)	Dictyoceratida;<br>Irciniidae;<br>*Ircinia* sp.
2-7	SAMA<br>S1982	∨	∨	KJ546358	Poecilosclerida;<br>Desmacididae;<br>*Desmapsamma anchorata*<br>(615, 99%, 93%)	∨	∨	KJ620382	Poecilosclerida;<br>Tedaniidae;<br>*Tedania tubulifera*<br>(979 ∨, 98%, 98%)	∨	∨	-	Poecilosclerida;<br>Tedaniidae;<br>*Tedania ignis*<br>(115, 90%, 20%)	Poecilosclerida;<br>Chondropsidae;<br>*Chondropsis* sp.
2-8	SAMA<br>S1983	∨	∨	KJ546359	Tetractinellida;<br>Ancorinidae;<br>*Ancorina* sp.<br>(600, 98%, 94%)	∨	∨	KJ620383	Tetractinellida;<br>Ancorinidae;<br>*Tethyopsis mortenseni*<br>(967 ∨, 98%, 97%)	∨	∨	-	Tetractinellida;<br>Geodiidae;<br>*Pachymatisma johnstonia*<br>(153, 96%, 17%)	Tetractinellida;<br>Geodiidae;<br>Geodiid sp.
2-9	SAMA<br>S1984	∨	∨	KJ546360	Poecilosclerida;<br>Desmacididae;<br>*Desmapsamma anchorata*<br>(612, 98%, 96%)	∨	∨	KJ620384	Poecilosclerida;<br>Tedaniidae;<br>*Tedania tubulifera*<br>(870 ∨, 95%, 92%)	∨	×		CANCEL<br>(The other two are all valid and matched at the Order level.)	Poecilosclerida;<br>Chondropsidae;<br>*Chondropsis* sp.
2-10	SAMA<br>S1987	∨	∨	KJ546362	Poecilosclerida;<br>Podospongiidae;<br>*Diacarnus spinipoculum*<br>(173, 75%, 97%)	∨	∨	KJ620385	Poecilosclerida;<br>Mycalidae;<br>*Mycale setosa*<br>(305 ∨, 84%, 55%)	×	×		CANCEL<br>(The other two are all valid and matched at the Order level.)	Poeciliosclerida;<br>Mycalidae;<br>*Mycale* (*Zygomycale*) sp.
2-11	SAMA<br>S1976	∨	∨	KJ546365	Suberitida;<br>Halichondriidae;<br>*Halichondria okadai*<br>(576, 96%, 95%)	∨	×		CANCEL<br>(The other two are all valid and matched at the species level.)	∨	∨	KJ801656	Suberitida;<br>Halichondriidae;<br>*Halichondria okadai*<br>(617 ∨, 94%, 99%)	Chondrillida<br>Chondrillidae<br>*Chondrosia* sp.
2-12	SAMA<br>S1961	∨	∨	KJ620400	Poecilosclerida;<br>Podospongiidae;<br>*Diacarnus spinipoculum*<br>(657, 99%, 97%)	∨	∨	KJ620387	Poecilosclerida;<br>Podospongiidae;<br>*Diacarnus spinipoculum*<br>(953 ∨, 99%, 92%)	∨	∨	-	Poecilosclerida;<br>Tedaniidae;<br>*Tedania ignis*<br>(153, 92%, 21%)	Clionaida;<br>Spirastrellidae;<br>*Spheciospongia* sp.
2-13	SAMA<br>S1962	∨	∨	KJ620401	Tetractinellida;<br>Ancorinidae;<br>*Ecionemia robusta*<br>(1010 ∨, 99%, 99%)	∨	∨	KJ620388	Tetractinellida;<br>Ancorinidae;<br>*Stelletta clavosa*<br>(642, 99%, 95%)	∨	∨	-	Tetractinellida;<br>Geodiidae;<br>*Pachymatisma johnstonia*<br>(154, 97%, 18%)	Tetractinellida;<br>Ancorinidae;<br>*Ecionemia* sp.
2-14	SAMA<br>S1963	∨	∨	KJ620402	Tetractinellida;<br>Ancorinidae;<br>*Ecionemia* sp.<br>(641, 99%, 94%)	∨	∨	KJ620389	Tetractinellida;<br>Ancorinidae;<br>*Stelletta clavosa*<br>(1023 ∨, 99%, 97%)	∨	∨	-	Tetractinellida;<br>Tetillidae;<br>*Cinachyrella apion*<br>(169, 98%, 18%)	Tetractinellida;<br>Ancorinidae;<br>Ancorinid sp.

2-15	SAMA<br>S1964	∨	∨	KJ620403	Tethyida;<br>Tethyidae;<br>*Tethya californiana*<br>(576, 96%, 96%)	∨	∨	KJ620390	Tethyida;<br>Tethyidae;<br>*Tethya* sp.<br>(973 ∨, 98%, 98%)	∨	∨	-	Tethyida;<br>Tethyidae;<br>*Tethya* sp.<br>(163, 100%, 38%)	Tethyida;<br>Tethyidae;<br>*Tethya* cf. *bergquistae*
2-16	SAMA<br>S1966	∨	∨	KJ620405	Poecilosclerida;<br>Mycalidae;<br>*Mycale mirabilis*<br>(633, 99%, 94%)	∨	∨	KJ620392	Poecilosclerida;<br>Mycalidae;<br>*Mycale setosa*<br>(1008 ∨, 99%, 85%)	∨	∨	-	Agelasida;<br>Agelasidae;<br>*Agelas schmidti*<br>(132, 93%, 167/912=18%)	Poecilosclerida;<br>Mycalidae;<br>*Mycale* sp.
2-17	SAMA<br>S1968	∨	∨	KJ620406	Poecilosclerida;<br>Microcionina;<br>*Clathria rugosa*<br>(510 ∨, 93%, 94%)	∨	×		CANCEL<br>(The other two are all available.)	∨	∨	KJ801658	Tetractinellida;<br>Ancorinidae;<br>*Stryphnus mucronatus*<br>(380, 90%, 65%)	Tetractinellida;<br>Astrophorin sp.
1-1	SAMA<br>S1985	∨	∨	KJ546361	Verongiida;<br>Pseudoceratinidae;<br>*Pseudoceratina* sp.<br>(634 ∨, 99%, 95%)	∨	×		Try the second pair of primers<br>(Negative)	∨	×		Try other primers<br>(Not done in this study.)	Verongiida;<br>Pseudoceratinidae;<br>*Pseudoceratina* sp.
1-2	SAMA<br>S1988	∨	∨	KJ546363	Verongiida;<br>Pseudoceratinidae;<br>*Pseudoceratina* sp.<br>(619 ∨, 99%, 93%)	×	×		Try the second pair of primers<br>(Negative)	∨	∨	-	R: not belong to Porifera	Verongiida;<br>Pseudoceratinidae;<br>*Pseudoceratina* sp.
1-3	SAMA<br>S1975	∨	∨	KJ546364	Poecilosclerida;<br>Hymedesmiidae;<br>*Phorbas bihamiger*<br>(469 ∨, 91%, 95%)	×	×		Try the second pair of primers<br>(Negative)	×	×		Try other primers<br>(Not done in this study.)	Poecilosclerida;<br>Crellidae;<br>*Crella* sp.
1-4	SAMA<br>S1977	∨	∨	KJ546366	Poecilosclerida;<br>Hymedesmiidae;<br>*Phorbas bihamiger*<br>(453 ∨, 91%, 90%)	×	×		Try the second pair of primers<br>(Negative)	×	×		Try other primers<br>(Not done in this study.)	Poecilosclerida;<br>Crellidae;<br>*Crella* sp.
1-5	SAMA<br>S1978	∨	∨	KJ546367	Poecilosclerida;<br>Desmacididae;<br>*Desmapsamma anchorata*<br>(581 ∨, 97%, 93%)	×	×		Try the second pair of primers<br>(Negative)	×	×		Try other primers<br>(Not done in this study.)	Poecilosclerida;<br>Chondropsidae;<br>*Chondropsis* sp.
1-6	SAMA<br>S1979	∨	∨	KJ546368	Dendroceratida;<br>Dictyodendrillidae;<br>*Acanthodendrilla australis*<br>(526 ∨, 97%, 84%)	×	×		Try the second pair of primers<br>(Negative)	∨	∨	-	R: not belong to Porifera	Dendroceratida;<br>Dictyodendrillidae;<br>*Acanthodendrilla* sp.
1-7	SAMA<br>S1980	∨	∨	KJ620398	Suberitida;<br>Suberitidae;<br>'*Protosuberites*' sp.<br>(404 ∨, 87%, 96%)	×	×		Try the second pair of primers<br>(Negative)	∨	×		Try other primers<br>(Not done in this study.)	Haplosclerida;<br>Chalinidae;<br>*Haliclona* sp.
1-8	SAMA<br>S1967	∨	∨	-	F: not belong to Porifera	∨	∨	KJ620393	Haplosclerida;<br>Petrosiidae;<br>*Petrosia lignosa*<br>(826 ∨, 93%, 96%)	∨	∨	-	Haplosclerida;<br>Niphatidae;<br>*Amphimedon chloros*<br>(130, 93%, 19%)	Haplosclerida;<br>Callyspongiidae;<br>*Callyspongia bilamellata*

1-9	SAMA<br>S1969	×	×		Try nested-PCR<br>(Negative)	×	×		Try the second pair of primers<br>(Positive)	∨	∨	KJ801659	Dictyoceratida;<br>Irciniidae;<br>*Ircinia felix* f. *felix*<br>(358 ∨, 87%, 70%)	Dictyoceratida;<br>Spongiid sp.
1-10	SAMA<br>S1970	×	×		Try nested-PCR<br>(Negative)	×	×		Try the second pair of primers<br>(Negative)	∨	∨	KJ801660	Dictyoceratida;<br>Irciniidae;<br>*Ircinia felix* f. *felix*<br>(349 ∨, 81%, 99%)	Dictyoceratida;<br>Thorectid sp.
1-11	SAMA<br>S1972	∨	∨	KJ620408	Axinellida;<br>Raspailiidae;<br>*Echinodictyum cancellatum*<br>(467 ∨, 93%, 85%)	∨	∨	-	R: not belong to Porifera	∨	∨	-	Axinellida;<br>Axinellidae;<br>*Acanthella pulcherrima*<br>(250, 95%, 36%)	Axinellida;<br>Raspailiidae;<br>*Echinodictyum mesenterinum*
1-12	SAMA<br>S1974	×	×		Try nested-PCR<br>(Negative)	×	×		Try the second pair of primers<br>(positive)	∨	∨	KJ801661	Dictyoceratida;<br>Irciniidae;<br>*Ircinia felix* f. *felix*<br>(389 ∨, 83%, 100%)	Dictyoceratida;<br>Irciniidae;<br>*Ircinia* sp.
0-1	SAMA<br>S1990	×	×		Try nested-PCR<br>(Negative)	×	×		Try the second pair of primers<br>(Positive)	∨	∨	-	Dendroceratida;<br>Dictyodendrillidae;<br>*Igernella notabilis*<br>(163, 99%, 19%)	Dendroceratida;<br>Darwinellidae ;<br>*Aplysilla rosea*
0-2	SAMA<br>S1992	∨	∨	-	Poecilosclerida;<br>Microcionidae;<br>*Microciona prolifera*<br>(124, 97%, 80%)<br>F: only 170 bp in total	×	×		CANCEL<br>(The other two are all available.)	∨	∨	-	Poecilosclerida;<br>Microcionidae;<br>*Microciona prolifera*<br>(109, 91%, 155/853=18%)	Poecilosclerida;<br>Poecilosclerid sp.
0-3	SAMA<br>S1986	×	×		CANCEL<br>(The other two are all available.)	∨	∨	-	Haplosclerida;<br>unclassified Haplosclerida;<br>*Haplosclerine* sp.<br>(705, 88%, 93%)<br>R: not belong to Porifera	∨	∨	-	Haplosclerida;<br>Niphatidae;<br>*Amphimedon queenslandica*<br>(119, 91% , 24%)<br>F: not belong to Porifera	Haplosclerida;<br>Chalinidae;<br>*Chalinula* sp.

Supplementary Table S4. **Trial results of alternative primers for the 28S rRNA gene amplification on the failed sponges and the sampling locations**

	Sponge SAMA S1990, S1993<br>Rapid Bay	Sponge SAMA S1985, S1988<br>Outer Harbour	Sponge SAMA S1975, S1977, S1978, S1979, S1980<br>Klein Point	Sponge SAMA S1969, S1970, S1974<br>Williams Island
**Specimen working with alternative primers**	SAMA S1990<br>SAMA S1993	-	-	SAMA S1969<br>SAMA S1974
**Success rate**	2/2	0/2	0/5	2/3
**Overall successful rate**		4/12		

Supplementary Table S5. Morphological descriptions of thirty-seven sponges

Museum Voucher	Morphological classification	Brief morphological description	Category
**SAMA S1962**	*Ecionemia* sp.	Massive; russet brown live, cream in ethanol; firm; oscules on ridge. Anatriaenes, oxeas, spiny microrhabds, oxyasters.	Cat I
**SAMA S1964**	*Tethya* cf. *bergquistae*	Small spherical (3.5cm diam.); orange externally; brown internally; thick cortex (5 mm).	Cat I
**SAMA S1966**	*Mycale* (*Arenochalina*) sp.	Red fibres. Mucoid. Very soft. Falls apart out of water. Large open oscules. Mycalostyles, no microscleres.	Cat I
**SAMA S1972**	*Echinodictyum mesenterinum*	Stalked; bilaminate; sculptured ventral surface; reddish brown. Oxeas (many), acanthostyles (many) some with swollen tip.	Cat I
**SAMA S1973**	*Aplysina lendenfeldi*	Erect; digitate/lobate; black (oxidised) oscule on top of lobe; slimy to touch; fibrous. Yellow internally oxidating to dark blue.	Cat I
**SAMA S1974**	*Ircinia* sp.	Massive; black surface; creamy yellow inside; firm-compressible. Irciniid filaments.	Cat I
**SAMA S1981**	*Suberites* sp.	Digitate; orange; very firm, barely compressible; oscules 0.5 - 1 mm diam. obscured by emerging hydroid.	Cat I
**SAMA S1987**	*Mycale* (*Zygomycale*) sp.	Small soft lobes; heavily fouled; oscules inconspicuous. Styles/ mycalostyles (465 µm), Anisochelae (45 µm). Palmate isochelae (15 µm)	Cat I
**SAMA S1996**	*Ircinia* sp.	Massive, with ventral crater; beige; conulose; tough/firm.	Cat I
**SAMA S1960**	*Cliona* sp.	Massive; bright orange; orange internally; firm; oscules 3-4 mm diam. No pore sieves seen. Spicules are tylostyles (tyle on top or just below top of spicule) and spirasters. This is put in Clionaidae as it is not encrusting.	Cat II
**SAMA S1971**	*Callyspongia* (*Callyspongia*) sp.	Digitate; beige with pink tinge; soft; 2-3 mm oscules along branches. Very firm, springy. Very little tissue between fibres. Strong multispicular fibre network, different size meshes on surface, and not so many spicules. Oxeas very small (60 µm).	Cat II
**SAMA S1975**	*Crella* sp. 1	Springy small; beige with pink tinge; beige in ethanol; oscules in recesses 2 mm diam; Spicules are tornotes in bunches, acanthostyles, arcuate chelae. Ectosomal tangential layer of acanthostyles.	Cat II
**SAMA S1977**	*Crella* sp. 1	Small sponge lacunate structure; beige with pink tinge; beige in ethanol; oscules in recesses 2 mm diam. Acanthostyles (60 µm), oxeas - tornotes (165 µm) no styles. Arcuate chelae.	Cat II
**SAMA S1978**	*Chondropsis* sp.	Lobate to amorphous; beige; skin-like ectosome, does not feel arenaceous; firm not soft. Surface armoured with sand and foreign spicules, the only whole spicules are thin strongyles.	Cat II
**SAMA S1989**	*Aplysilla rosea*	Pink, fleshy, conulose, encrusting.	Cat II
**SAMA S1995**	*Echinodictyum mesenterinum*	Bilamellate fan; brown; rugose; firm, oxeas (many) and acanthostyles (few) with swelling at base end.	Cat II
**SAMA S1963**	Ancorinid sp.	Spherical; dark red with crustose red algae on surface; thick cortex; beige internally; firm; oscules 5 mm diam. Oxeas, euasters, microrhabds (no triaenes).	Cat III
**SAMA S1965**	*Caulospongia* sp.	Stalked with pagoda-like branches; light brown; easily compressible. Spicules are tylostyles with flattened tyles.	Cat III
**SAMA S1982**	*Chondropsis* sp.	Lobate; beige; compressible. Oscule at top of lobe. Foreign spicules and strongyles.	Cat III
**SAMA S1983**	Geodiid sp.	Spherical; black; hard; thick cortex (4 mm), brown internally. Triaenes, oxeas, oxyasters, microrhabds, surface sterrasters.	Cat III
**SAMA S1984**	*Chondropsis* sp.	Lobate; beige; quite thick surface armour; oscules not seen. Spicules are strongyles, sigmas and chelae. Very soft.	Cat III

**SAMA S1991**	*Chondropsis* sp.	Lobate; brown; firm-compressible; oscules 6 mm diam. on top of lobes. Whole spicules are strongyles (not numerous) and sigmas.	Cat III
**SAMA S1992**	*Tedania* cf. *anhelans*	Red; firm-compressible; skin-like ectosome. Microspined tylotes, styles, onychaetes (with no tyle).	Cat III
**SAMA S1994**	*Chondropsis* sp.	Lobate; orange; firm-compressible; oscules 3 mm diam. at tip of lobes.	Cat III
**SAMA S1961**	*Spheciospongia* sp.	Massive; pale mottled; very firm; thick skin, ectosomal layer; oscules on ridge 2-3 mm diam. Spicules are styles and diplasters (3 categories).	Cat IV
**SAMA S1968**	Astrophorin sp.	Massive, pale. Encrusted by soft orange sponge. Thick cortex. Oscules on peaks 2 mm diam. Large oxeas, sanidasters, no oxyasters.	Cat IV
**SAMA S1980**	*Haliclona* sp.	Creeping digitate; soft; oscules 1mm diam. along branches. Regular ladder-like network - joining fibres one spicule long. Very thin oxeas (90-120 µm).	Cat IV
**SAMA S1985**	*Pseudoceratina* sp.	Massive; black (oxidised); firm/hard; many small blunt conules close together. Fibres heavily cored with sand. Very slightly lighter inside then externally. Sand in fibres. Although the sponge tissue looks solid, under the microscope it is lacunose in areas. Fibre walls are not thick.	Cat V
**SAMA S1988**	*Pseudoceratina* sp.	Massive; firm/hard; black (oxidised); blunt conules. Pigment granules.	Cat V
**SAMA S1979**	*Acanthodendrilla* sp.	Encrusting; pink; soft, but not slippery; conulose. Fibres are reticulate (but not neatly) Many whole poecilosclerid spicules (chelae, styles, acanthostyles) in tissue. Surface is armoured and some of the primary fibres completed cored with sand.	Cat V
**SAMA S1967**	*Callyspongia bilamellata*	Bilaminate; stalked; russet brown; sculptured underside; sticky to touch; Pale brown inside. Spicules are oxeas.	Cat V
**SAMA S1969**	Spongiid sp.	Erect; bright orange. Firm-compressible. Fibres obvious through ectosome. Clear fibres. Dictyoceratida, Spongiidae. No irciniid fibres.	Cat V
**SAMA S1970**	Thorectid sp.	Fibrous. Slightly mucoid. Pale. Striated surface. Oscules recessed. Laminated reticulate uncored fibres. Fibres darker than choanosome.	Cat V
**SAMA S1967**	*Chondrosia* sp.	Spherical; brown externally (ectosomal skin), beige internally. Compressible. Spicules seem to be foreign, mixed eg. oxeas, styles, anisooxeas, acanthostyles and many broken spicules. Fibrous choanosome.	Cat V

Supplementary Table S6. The submissions of the three DNA loci based on different Porifera orders in the NCBI database. The data were retrieved by the 'Advanced' search under 'Nucleotide' category in the NCBI database. The particular gene and sponge taxonomic group were used as the factors to conduct the search. The sequences numbers were checked manually and calculated.

Porifera Order	COI mtDNA Submissions	28S rRNA gene Submissions	ITS region Submissions
**Haplosclerida**	717	374	15
**Poecilosclerida**	280	502	27
**Tetractinellida**	237	272	27
**Dictyoceratida**	127	292	159
**Suberitida**	98	178	159
**Verongiida**	87	177	147
**Tethyida**	30	77	11
**Axinellida**	28	258	37
**Clionaida**	26	379	367
**Dendroceratida**	6	31	15
**Total**	1636	2540	964

Supplementary Table S7. The nucleotide submissions from different sponge families, genera and species in the NCBI database.

Porifera Order	Family	Genus	Species	COI mtDNA nucleotide No.	28S rRNA gene nucleotide No.	ITS region nucleotide No.
Haplosclerida	Callyspongiidae	*Callyspongia*	*Callyspongia siphonella*	1	1	0
			Other species	561	74	0
		Other genera	-	1	5	0
	Chalinidae	*Cladocroce*	*Cladocroce* sp.	0	2	0
			Other species	1	3	0
		Haliclona	*Haliclona* sp.	4	23	2
			Other species	37	30	5
		Other genera	-	0	26	0
Poecilosclerida	Desmacididae	*Desmapsamma*	*Desmapsamma anchorata*	7	2	0
			Other species	0	0	0
		Other genera	-	0	0	0
	Tedaniidae	*Tedania*	*Tedania tubulifera*	0	1	0
			Tedania ignis	12	3	1
			Other species	6	3	0
		Other genera	-	0	0	0
Tetractinellida	Ancorinidae	*Ancorina*	*Ancorina* sp.	1	1	0
			Other species	0	2	0
		Tethyopsis	*Tethyopsis mortenseni*	0	1	0
			Other species	0	1	0
		Other genera	-	31	45	3
	Geodiidae	*Pachymatisma*	*Pachymatisma johnstonia*	12	9	7
			Other species	8	6	5
		Other genera	-	86	41	2

Supplementary Table S8. Sponges with any two matched identities among the three DNA loci and the morphological classification at different taxonomic levels. COI for COI mtDNA, 28S for 28S rRNA gene, ITS for nuclear ITS region and M for morphological classification.

Order level match			
**No.**	**Sponge Code**	**Loci providing the identities**	**Identity Information (Order)**
1	SAMA S1991	COI, 28S, M	Poecilosclerida
2	SAMA S1993	COI, M	
3	SAMA S1982	COI, 28S, M	
4	SAMA S1984	COI, 28S, M	
5	SAMA S1975	COI, M	
6	SAMA S1977	COI, M	
7	SAMA S1978	COI, M	
8	SAMA S1968	ITS, M	Tetractinellida
9	SAMA S1970	ITS, M	Dictyoceratida
10	SAMA S1969	ITS, M	
11	SAMA S1967	28S, M	Haplosclerida

Family level match				
**No.**	**Sponge Code**	**Loci providing the identities**	**Identity Information (Family-Order)**	
1	SAMA S1983	COI, 28S	Ancorinidae	Tetractinellida
2	SAMA S1963	COI, M		

Genus level match					
**No.**	**Sponge Code**	**Loci providing the identities**	**Identity Information (Genus-Family-Order)**		
1	SAMA S1994	COI, 28S	*Tedania*	Tedaniidae	Poecilosclerida
2	SAMA S1987	28S, M	*Mycale*	Mycalidae	
3	SAMA S1966	COI, 28S, M			
4	SAMA S1995	COI, M	*Echinodictyum*	Raspailiidae	Axinellida
5	SAMA S1972	COI, M			
6	SAMA S1981	28S, M	*Suberite*	Suberitidae	Suberitida
7	SAMA S1964	COI, 28S, M	*Tethya*	Tethyidae	Tethyida
8	SAMA S1971	COI, M	*Callyspongia*	Callyspongiidae	Haplosclerida
9	SAMA S1996	28S, ITS, M	*Ircinia*	Irciniidae	Dictyoceratida
10	SAMA S1974	ITS, M			
11	SAMA S1962	COI, M	*Ecionemia*	Ancorinidae	Tetractinellida
12	SAMA S1973	COI, 28S, M	*Aplysina*	Aplysinidae	Verongiida
13	SAMA S1985	COI, M	*Pseudoceratina*	Pseudoceratinidae	
14	SAMA S1988	COI, M			
15	SAMA S1979	COI, M	*Acanthodendrilla*	Dictyodendrillidae	Dendroceratida

Species level match					
**No.**	**Sponge Code**	**Loci providing the identities**	**Identity Information (Species-Family-Order)**		
1	SAMA S1965	28S, ITS	*Hymeniacidon heliophila*	Hymeniacidonidae	Suberitida
2	SAMA S1976	COI, ITS	*Halichondria okadai*	Halichondriidae	
3	SAMA S1960	28S, ITS	*Spirastrella hartmani*	Spirastrellidae	Clionaida
4	SAMA S1961	COI, 28S	*Diacarnus spinipoculum*	Podospongiidae	Poecilosclerida
5	SAMA S1989	COI, 28S	*Igernella notabilis*	Dictyodendrillidae	Dendroceratida

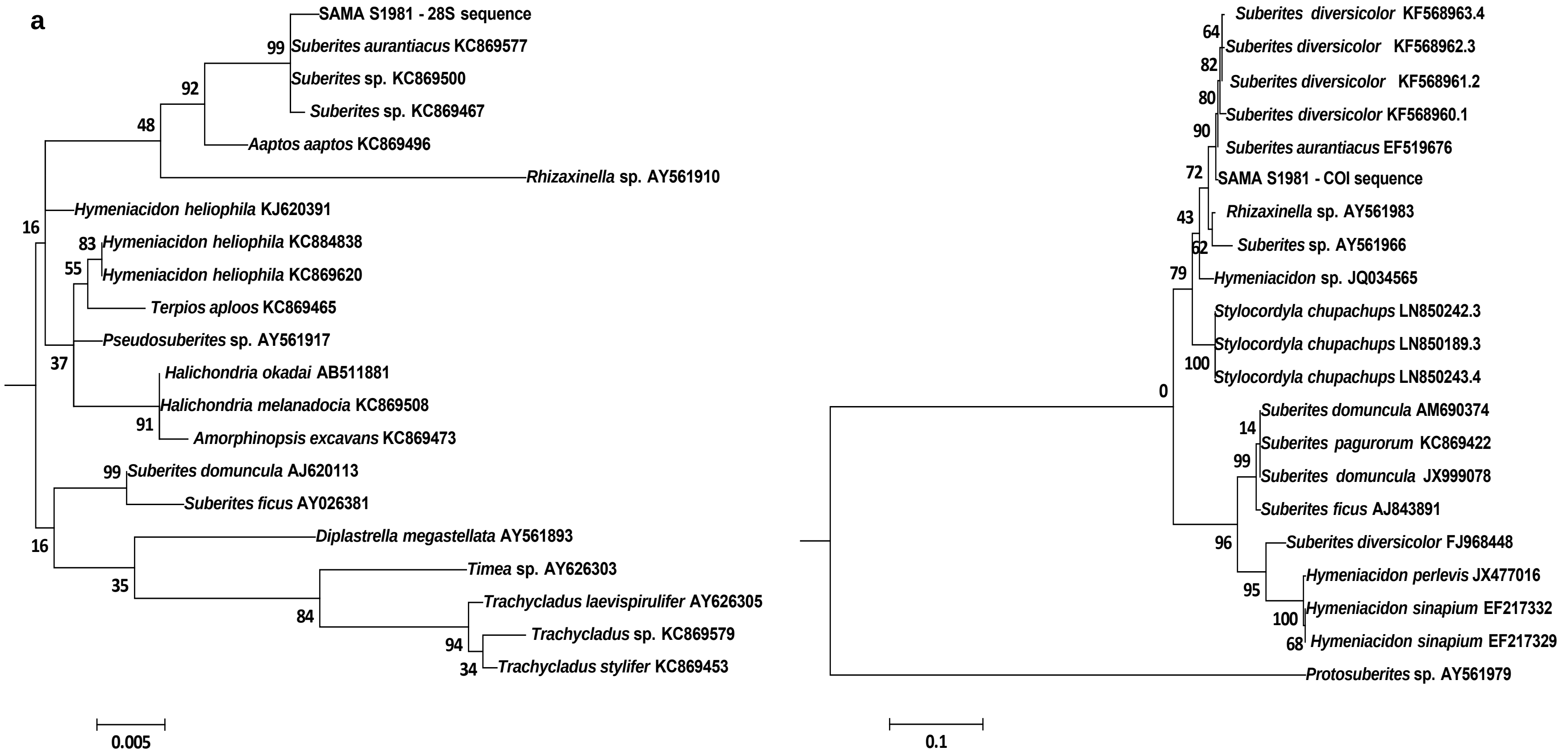
a
SAMA S1981 - 28S sequence
Suberites aurantiacus KC869577
Suberites sp. KC869500
Suberites sp. KC869467
Aaptos aaptos KC869496
Rhizaxinella sp. AY561910
Hymeniacidon heliophila KJ620391
Hymeniacidon heliophila KC884838
Hymeniacidon heliophila KC869620
Terpios aploos KC869465
Pseudosuberites sp. AY561917
Halichondria okadai AB511881
Halichondria melanadocia KC869508
Amorphinopsis excavans KC869473
Suberites domuncula AJ620113
Suberites ficus AY026381
Diplastrella megastellata AY561893
Timea sp. AY626303
Trachycladus laevispirulifer AY626305
Trachycladus sp. KC869579
Trachycladus stylifer KC869453
99
92
48
16
83
55
37
91
99
16
35
84
94
34
0.005
Suberites diversicolor KF568963.4
Suberites diversicolor KF568962.3
Suberites diversicolor KF568961.2
Suberites diversicolor KF568960.1
Suberites aurantiacus EF519676
SAMA S1981 - COI sequence
Rhizaxinella sp. AY561983
Suberites sp. AY561966
Hymeniacidon sp. JQ034565
Stylocordyla chupachups LN850242.3
Stylocordyla chupachups LN850189.3
Stylocordyla chupachups LN850243.4
Suberites domuncula AM690374
Suberites pagurorum KC869422
Suberites domuncula JX999078
Suberites ficus AJ843891
Suberites diversicolor FJ968448
Hymeniacidon perlevis JX477016
Hymeniacidon sinapium EF217332
Hymeniacidon sinapium EF217329
Protosuberites sp. AY561979
64
82
80
90
72
43
62
79
100
0
14
99
96
95
100
68
0.1

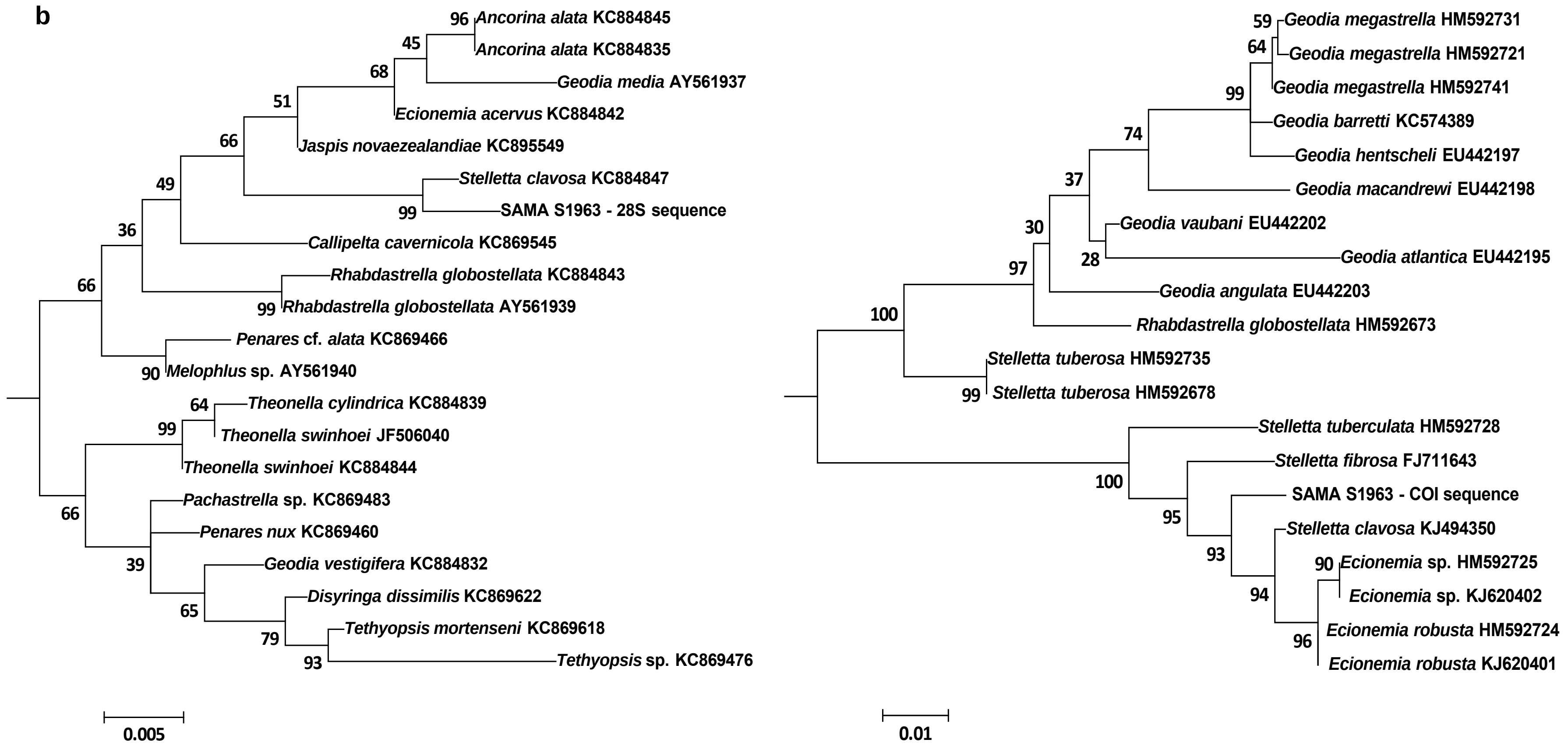
b
Ancorina alata KC884845
Ancorina alata KC884835
Geodia media AY561937
Ecionemia acervus KC884842
Jaspis novaezealandiae KC895549
Stelletta clavosa KC884847
SAMA S1963 - 28S sequence
Callipelta cavernicola KC869545
Rhabdastrella globostellata KC884843
Rhabdastrella globostellata AY561939
Penares cf. alata KC869466
Melophlus sp. AY561940
Theonella cylindrica KC884839
Theonella swinhoei JF506040
Theonella swinhoei KC884844
Pachastrella sp. KC869483
Penares nux KC869460
Geodia vestigifera KC884832
Disyringa dissimilis KC869622
Tethyopsis mortenseni KC869618
Tethyopsis sp. KC869476
0.005
Geodia megastrella HM592731
Geodia megastrella HM592721
Geodia megastrella HM592741
Geodia barretti KC574389
Geodia hentscheli EU442197
Geodia macandrewi EU442198
Geodia vaubani EU442202
Geodia atlantica EU442195
Geodia angulata EU442203
Rhabdastrella globostellata HM592673
Stelletta tuberosa HM592735
Stelletta tuberosa HM592678
Stelletta tuberculata HM592728
Stelletta fibrosa FJ711643
SAMA S1963 - COI sequence
Stelletta clavosa KJ494350
Ecionemia sp. HM592725
Ecionemia sp. KJ620402
Ecionemia robusta HM592724
Ecionemia robusta KJ620401
0.01

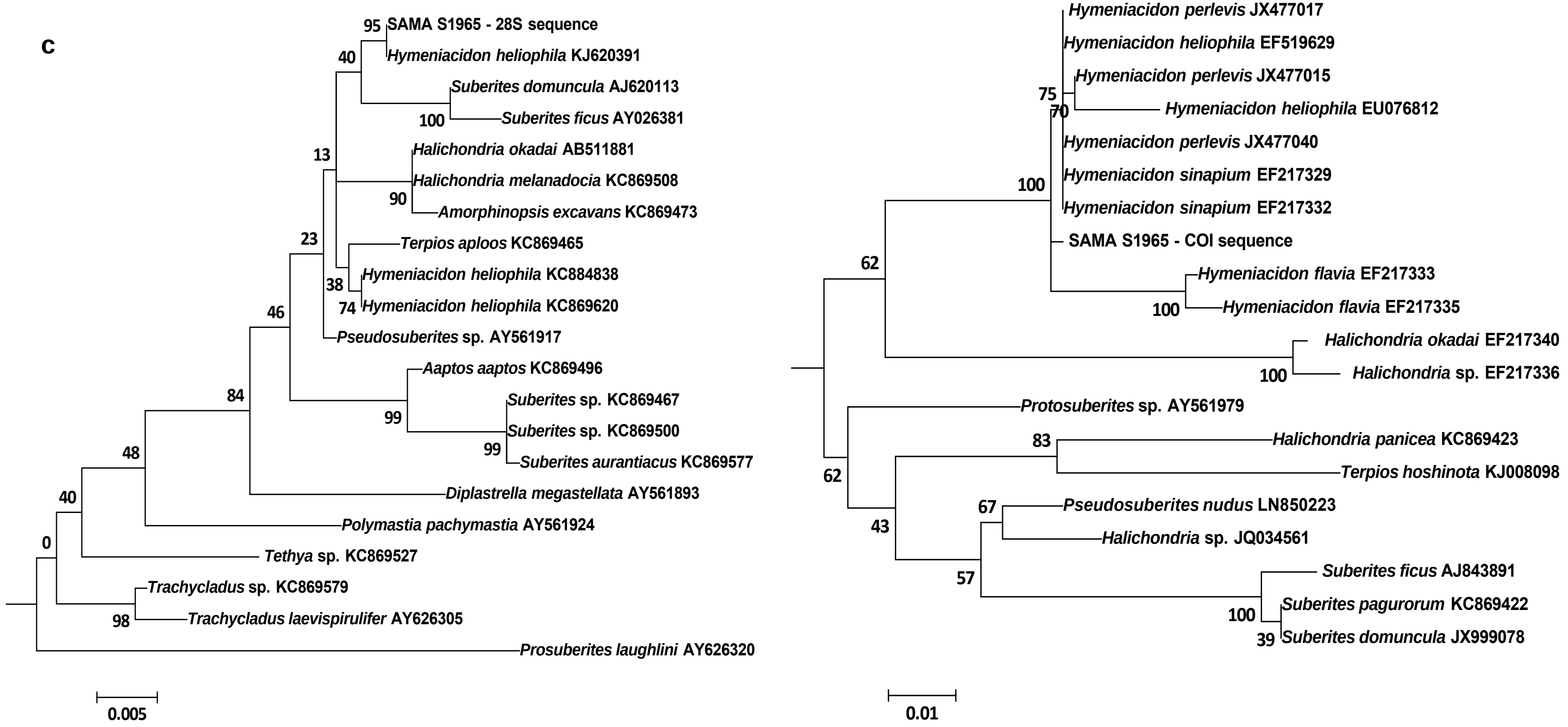
c
SAMA S1965 - 28S sequence
Hymeniacidon heliophila KJ620391
Suberites domuncula AJ620113
Suberites ficus AY026381
Halichondria okadai AB511881
Halichondria melanadocia KC869508
Amorphinopsis excavans KC869473
Terpios aploos KC869465
Hymeniacidon heliophila KC884838
Hymeniacidon heliophila KC869620
Pseudosuberites sp. AY561917
Aaptos aaptos KC869496
Suberites sp. KC869467
Suberites sp. KC869500
Suberites aurantiacus KC869577
Diplastrella megastellata AY561893
Polymastia pachymastia AY561924
Tethya sp. KC869527
Trachycladus sp. KC869579
Trachycladus laevispirulifer AY626305
Prosuberites laughlini AY626320
95
40
100
13
90
23
38
74
46
99
99
84
48
40
0
98
0.005
Hymeniacidon perlevis JX477017
Hymeniacidon heliophila EF519629
Hymeniacidon perlevis JX477015
Hymeniacidon heliophila EU076812
Hymeniacidon perlevis JX477040
Hymeniacidon sinapium EF217329
Hymeniacidon sinapium EF217332
SAMA S1965 - COI sequence
Hymeniacidon flavia EF217333
Hymeniacidon flavia EF217335
Halichondria okadai EF217340
Halichondria sp. EF217336
Protosuberites sp. AY561979
Halichondria panicea KC869423
Terpios hoshinota KJ008098
Pseudosuberites nudus LN850223
Halichondria sp. JQ034561
Suberites ficus AJ843891
Suberites pagurorum KC869422
Suberites domuncula JX999078
75
70
100
62
100
100
83
62
43
67
57
100
39
0.01

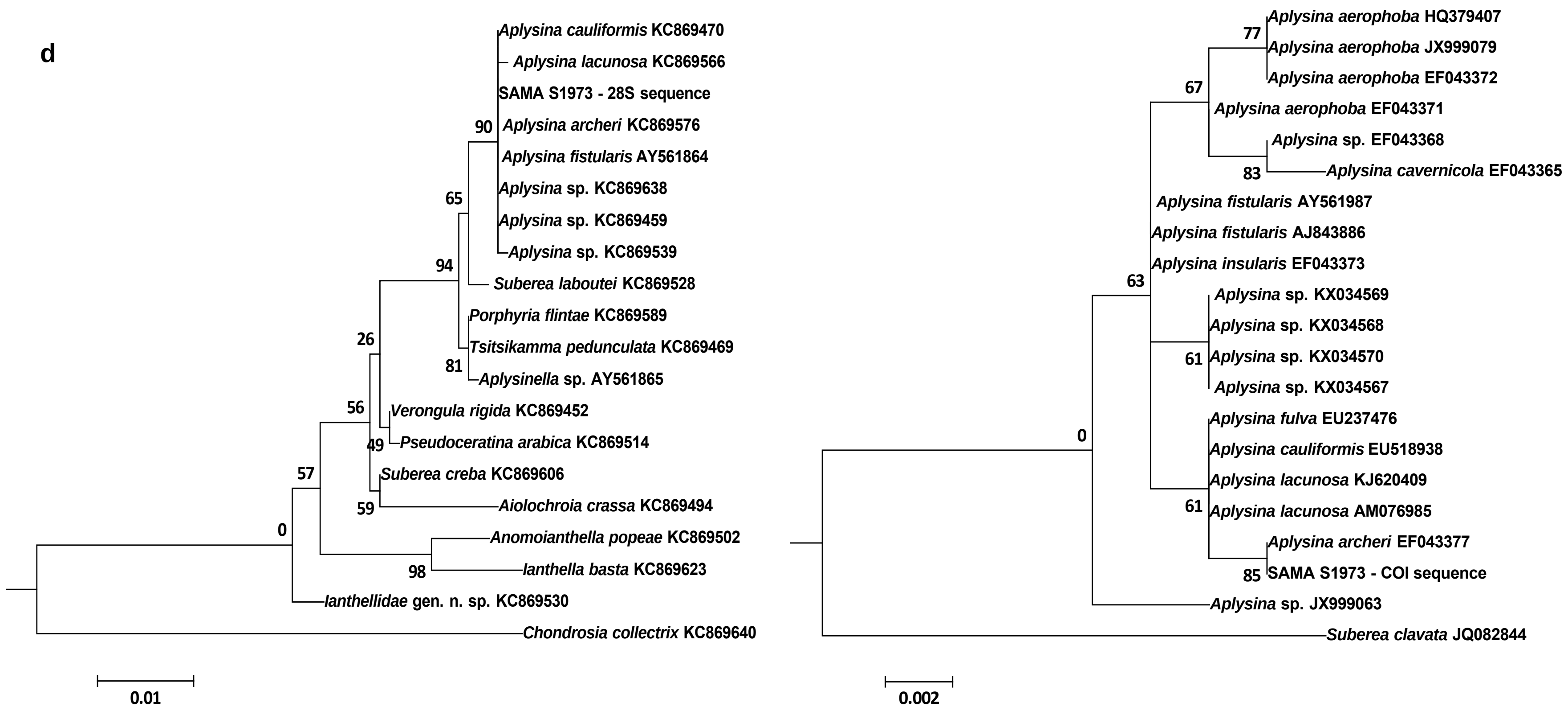
d
Aplysina cauliformis KC869470
Aplysina lacunosa KC869566
SAMA S1973 - 28S sequence
Aplysina archeri KC869576
Aplysina fistularis AY561864
Aplysina sp. KC869638
Aplysina sp. KC869459
Aplysina sp. KC869539
Suberea laboutei KC869528
Porphyria flintae KC869589
Tsitsikamma pedunculata KC869469
Aplysinella sp. AY561865
Verongula rigida KC869452
Pseudoceratina arabica KC869514
Suberea creba KC869606
Aiolochroia crassa KC869494
Anomoianthella popeae KC869502
Ianthella basta KC869623
Ianthellidae gen. n. sp. KC869530
Chondrosia collectrix KC869640
90
65
94
81
26
56
49
59
57
0
98
0.01
Aplysina aerophoba HQ379407
Aplysina aerophoba JX999079
Aplysina aerophoba EF043372
Aplysina aerophoba EF043371
Aplysina sp. EF043368
Aplysina cavernicola EF043365
Aplysina fistularis AY561987
Aplysina fistularis AJ843886
Aplysina insularis EF043373
Aplysina sp. KX034569
Aplysina sp. KX034568
Aplysina sp. KX034570
Aplysina sp. KX034567
Aplysina fulva EU237476
Aplysina cauliformis EU518938
Aplysina lacunosa KJ620409
Aplysina lacunosa AM076985
Aplysina archeri EF043377
SAMA S1973 - COI sequence
Aplysina sp. JX999063
Suberea clavata JQ082844
77
67
83
63
61
0
61
85
0.002

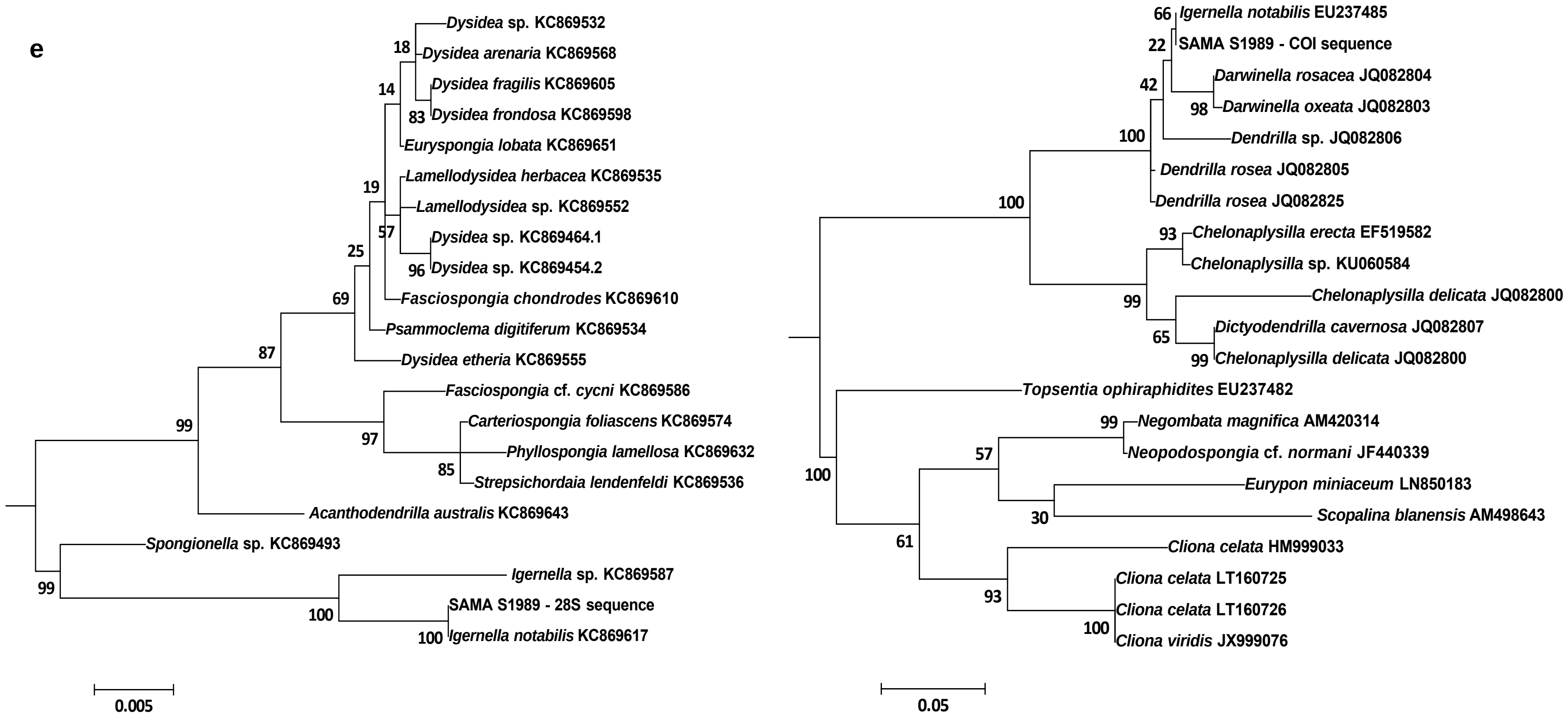
e
Dysidea sp. KC869532
Dysidea arenaria KC869568
Dysidea fragilis KC869605
Dysidea frondosa KC869598
Euryspongia lobata KC869651
Lamellodysidea herbacea KC869535
Lamellodysidea sp. KC869552
Dysidea sp. KC869464.1
Dysidea sp. KC869454.2
Fasciospongia chondrodes KC869610
Psammoclema digitiferum KC869534
Dysidea etheria KC869555
Fasciospongia cf. cycni KC869586
Carteriospongia foliascens KC869574
Phyllospongia lamellosa KC869632
Strepsichordaia lendenfeldi KC869536
Acanthodendrilla australis KC869643
Spongionella sp. KC869493
Igernella sp. KC869587
SAMA S1989 - 28S sequence
Igernella notabilis KC869617
0.005
Igernella notabilis EU237485
SAMA S1989 - COI sequence
Darwinella rosacea JQ082804
Darwinella oxeata JQ082803
Dendrilla sp. JQ082806
Dendrilla rosea JQ082805
Dendrilla rosea JQ082825
Chelonaplysilla erecta EF519582
Chelonaplysilla sp. KU060584
Chelonaplysilla delicata JQ082800
Dictyodendrilla cavernosa JQ082807
Chelonaplysilla delicata JQ082800
Topsentia ophiraphidites EU237482
Negombata magnifica AM420314
Neopodospongia cf. normani JF440339
Eurypon miniaceum LN850183
Scopalina blanensis AM498643
Cliona celata HM999033
Cliona celata LT160725
Cliona celata LT160726
Cliona viridis JX999076
0.05

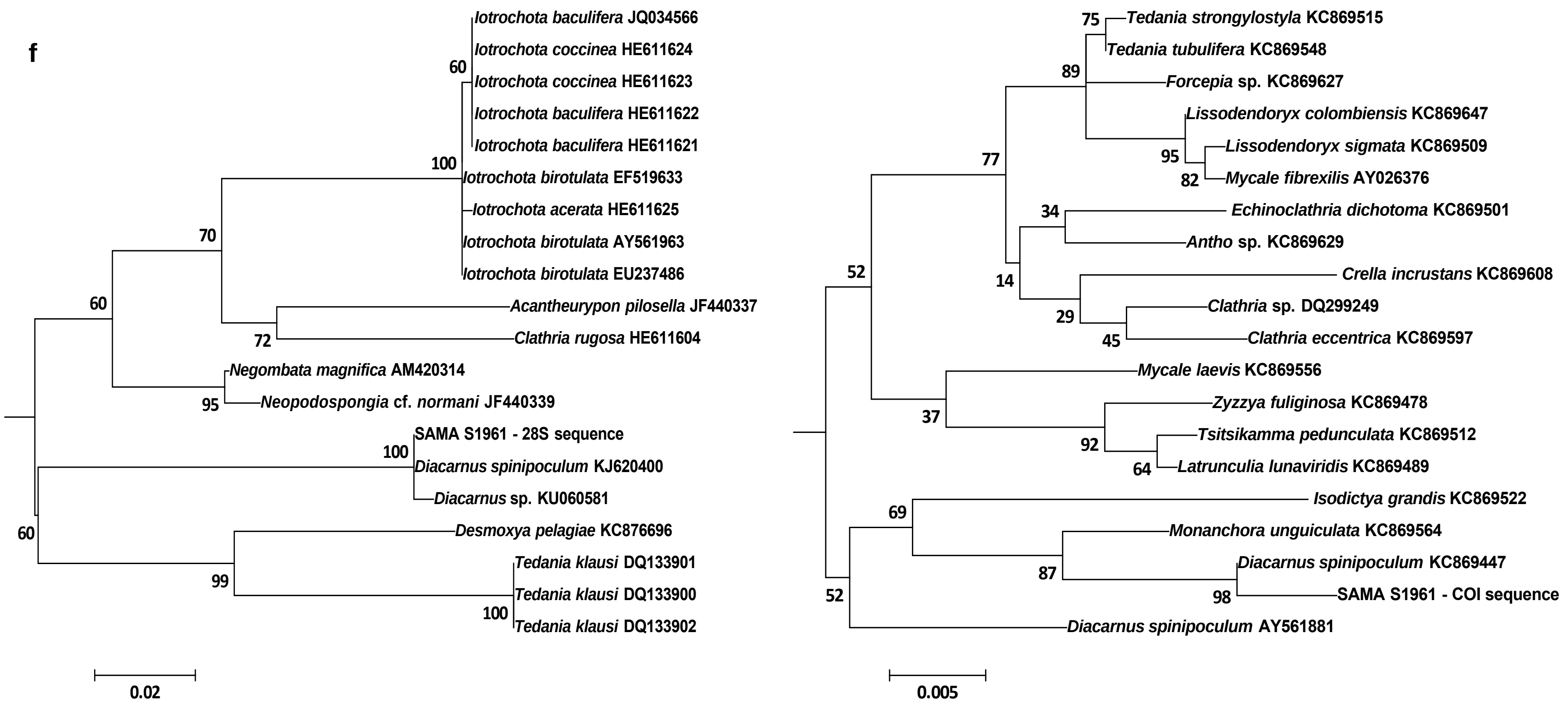
f
Iotrochota baculifera JQ034566
Iotrochota coccinea HE611624
Iotrochota coccinea HE611623
Iotrochota baculifera HE611622
Iotrochota baculifera HE611621
Iotrochota birotulata EF519633
Iotrochota acerata HE611625
Iotrochota birotulata AY561963
Iotrochota birotulata EU237486
Acantheurypon pilosella JF440337
Clathria rugosa HE611604
Negombata magnifica AM420314
Neopodospongia cf. normani JF440339
SAMA S1961 - 28S sequence
Diacarnus spinipoculum KJ620400
Diacarnus sp. KU060581
Desmoxya pelagiae KC876696
Tedania klausi DQ133901
Tedania klausi DQ133900
Tedania klausi DQ133902
60
100
70
60
72
95
100
60
99
100
0.02
Tedania strongylostyla KC869515
Tedania tubulifera KC869548
Forcepia sp. KC869627
Lissodendoryx colombiensis KC869647
Lissodendoryx sigmata KC869509
Mycale fibrexilis AY026376
Echinoclathria dichotoma KC869501
Antho sp. KC869629
Crella incrustans KC869608
Clathria sp. DQ299249
Clathria eccentrica KC869597
Mycale laevis KC869556
Zyzzya fuliginosa KC869478
Tsitsikamma pedunculata KC869512
Latrunculia lunaviridis KC869489
Isodictya grandis KC869522
Monanchora unguiculata KC869564
Diacarnus spinipoculum KC869447
SAMA S1961 - COI sequence
Diacarnus spinipoculum AY561881
75
89
95
82
77
34
14
52
29
45
37
92
64
69
87
98
52
0.005

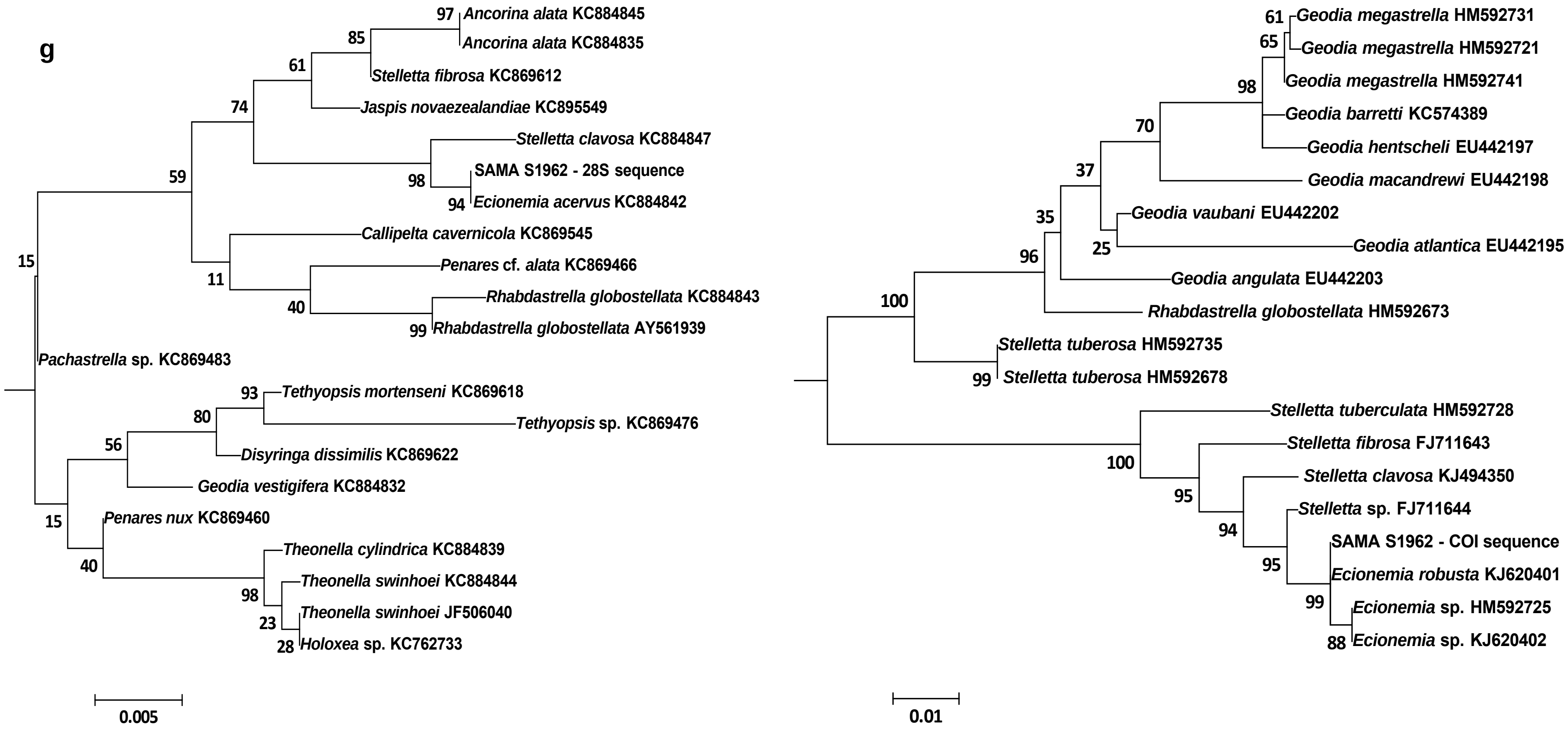
g
Ancorina alata KC884845
Ancorina alata KC884835
Stelletta fibrosa KC869612
Jaspis novaezealandiae KC895549
Stelletta clavosa KC884847
SAMA S1962 - 28S sequence
Ecionemia acervus KC884842
Callipelta cavernicola KC869545
Penares cf. alata KC869466
Rhabdastrella globostellata KC884843
Rhabdastrella globostellata AY561939
Pachastrella sp. KC869483
Tethyopsis mortenseni KC869618
Tethyopsis sp. KC869476
Disyringa dissimilis KC869622
Geodia vestigifera KC884832
Penares nux KC869460
Theonella cylindrica KC884839
Theonella swinhoei KC884844
Theonella swinhoei JF506040
Holoxea sp. KC762733
97
85
61
74
59
98
94
15
11
40
99
93
80
56
15
40
98
23
28
0.005
Geodia megastrella HM592731
Geodia megastrella HM592721
Geodia megastrella HM592741
Geodia barretti KC574389
Geodia hentscheli EU442197
Geodia macandrewi EU442198
Geodia vaubani EU442202
Geodia atlantica EU442195
Geodia angulata EU442203
Rhabdastrella globostellata HM592673
Stelletta tuberosa HM592735
Stelletta tuberosa HM592678
Stelletta tuberculata HM592728
Stelletta fibrosa FJ711643
Stelletta clavosa KJ494350
Stelletta sp. FJ711644
SAMA S1962 - COI sequence
Ecionemia robusta KJ620401
Ecionemia sp. HM592725
Ecionemia sp. KJ620402
61
65
98
70
37
35
25
96
100
99
100
95
94
95
99
88
0.01

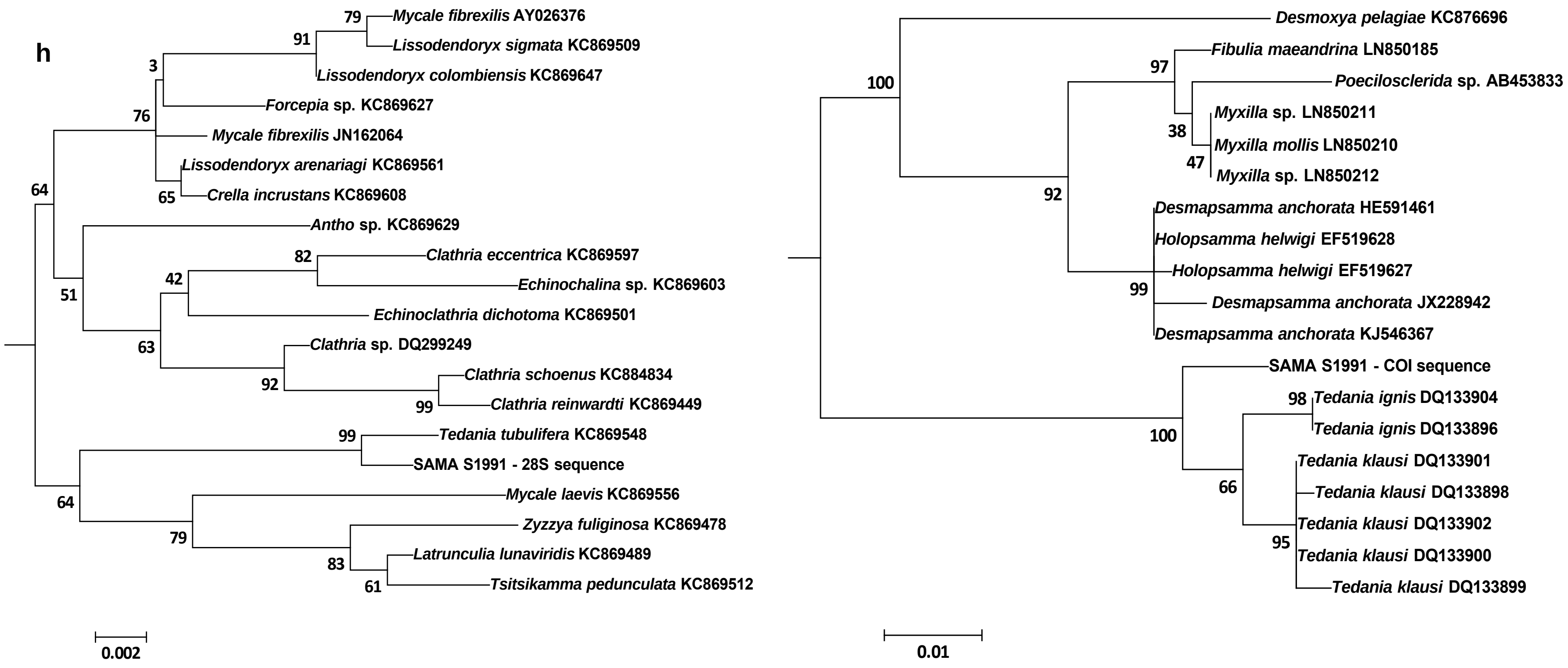
h
79
Mycale fibrexilis AY026376
91
Lissodendoryx sigmata KC869509
3
Lissodendoryx colombiensis KC869647
Forcepia sp. KC869627
76
Mycale fibrexilis JN162064
Lissodendoryx arenariagi KC869561
64
65
Crella incrustans KC869608
Antho sp. KC869629
82
Clathria eccentrica KC869597
42
Echinochalina sp. KC869603
51
Echinoclathria dichotoma KC869501
63
Clathria sp. DQ299249
Clathria schoenus KC884834
92
99
Clathria reinwardti KC869449
99
Tedania tubulifera KC869548
SAMA S1991 - 28S sequence
64
Mycale laevis KC869556
79
Zyzzya fuliginosa KC869478
83
Latrunculia lunaviridis KC869489
61
Tsitsikamma pedunculata KC869512
0.002
Desmoxya pelagiae KC876696
Fibulia maeandrina LN850185
97
100
Poecilosclerida sp. AB453833
Myxilla sp. LN850211
38
Myxilla mollis LN850210
47
Myxilla sp. LN850212
92
Desmapsamma anchorata HE591461
Holopsamma helwigi EF519628
Holopsamma helwigi EF519627
99
Desmapsamma anchorata JX228942
Desmapsamma anchorata KJ546367
SAMA S1991 - COI sequence
98
Tedania ignis DQ133904
Tedania ignis DQ133896
100
Tedania klausi DQ133901
66
Tedania klausi DQ133898
Tedania klausi DQ133902
95
Tedania klausi DQ133900
Tedania klausi DQ133899
0.01

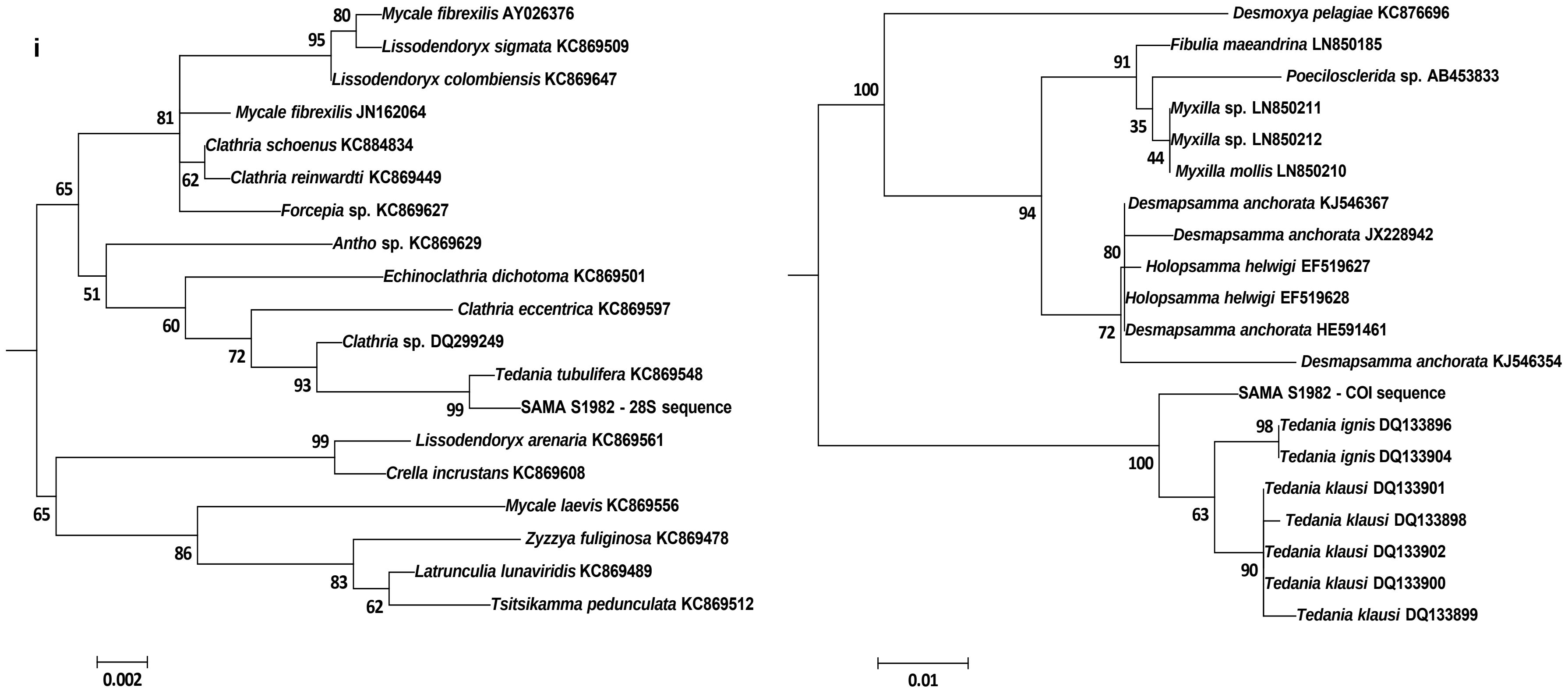
i
Mycale fibrexilis AY026376
Lissodendoryx sigmata KC869509
Lissodendoryx colombiensis KC869647
Mycale fibrexilis JN162064
Clathria schoenus KC884834
Clathria reinwardti KC869449
Forcepia sp. KC869627
Antho sp. KC869629
Echinoclathria dichotoma KC869501
Clathria eccentrica KC869597
Clathria sp. DQ299249
Tedania tubulifera KC869548
SAMA S1982 - 28S sequence
Lissodendoryx arenaria KC869561
Crella incrustans KC869608
Mycale laevis KC869556
Zyzzya fuliginosa KC869478
Latrunculia lunaviridis KC869489
Tsitsikamma pedunculata KC869512
0.002
Desmoxya pelagiae KC876696
Fibulia maeandrina LN850185
Poecilosclerida sp. AB453833
Myxilla sp. LN850211
Myxilla sp. LN850212
Myxilla mollis LN850210
Desmapsamma anchorata KJ546367
Desmapsamma anchorata JX228942
Holopsamma helwigi EF519627
Holopsamma helwigi EF519628
Desmapsamma anchorata HE591461
Desmapsamma anchorata KJ546354
SAMA S1982 - COI sequence
Tedania ignis DQ133896
Tedania ignis DQ133904
Tedania klausi DQ133901
Tedania klausi DQ133898
Tedania klausi DQ133902
Tedania klausi DQ133900
Tedania klausi DQ133899
0.01

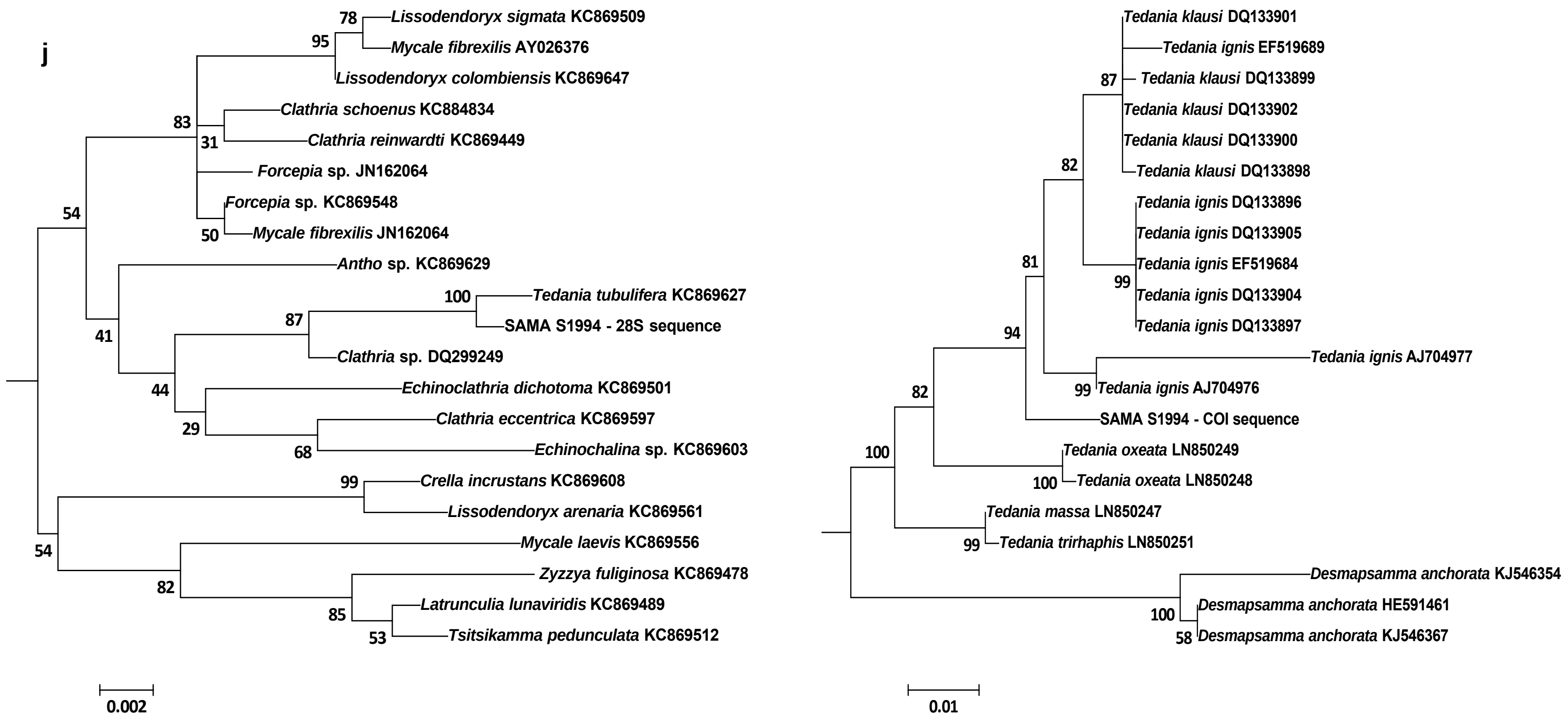
j
Lissodendoryx sigmata KC869509
Mycale fibrexilis AY026376
Lissodendoryx colombiensis KC869647
Clathria schoenus KC884834
Clathria reinwardti KC869449
Forcepia sp. JN162064
Forcepia sp. KC869548
Mycale fibrexilis JN162064
Antho sp. KC869629
Tedania tubulifera KC869627
SAMA S1994 - 28S sequence
Clathria sp. DQ299249
Echinoclathria dichotoma KC869501
Clathria eccentrica KC869597
Echinochalina sp. KC869603
Crella incrustans KC869608
Lissodendoryx arenaria KC869561
Mycale laevis KC869556
Zyzzya fuliginosa KC869478
Latrunculia lunaviridis KC869489
Tsitsikamma pedunculata KC869512
78
95
83
31
50
54
41
100
87
44
29
68
99
54
82
85
53
0.002
Tedania klausi DQ133901
Tedania ignis EF519689
Tedania klausi DQ133899
Tedania klausi DQ133902
Tedania klausi DQ133900
Tedania klausi DQ133898
Tedania ignis DQ133896
Tedania ignis DQ133905
Tedania ignis EF519684
Tedania ignis DQ133904
Tedania ignis DQ133897
Tedania ignis AJ704977
Tedania ignis AJ704976
SAMA S1994 - COI sequence
Tedania oxeata LN850249
Tedania oxeata LN850248
Tedania massa LN850247
Tedania trirhaphis LN850251
Desmapsamma anchorata KJ546354
Desmapsamma anchorata HE591461
Desmapsamma anchorata KJ546367
87
82
81
99
94
99
82
100
100
99
100
58
0.01

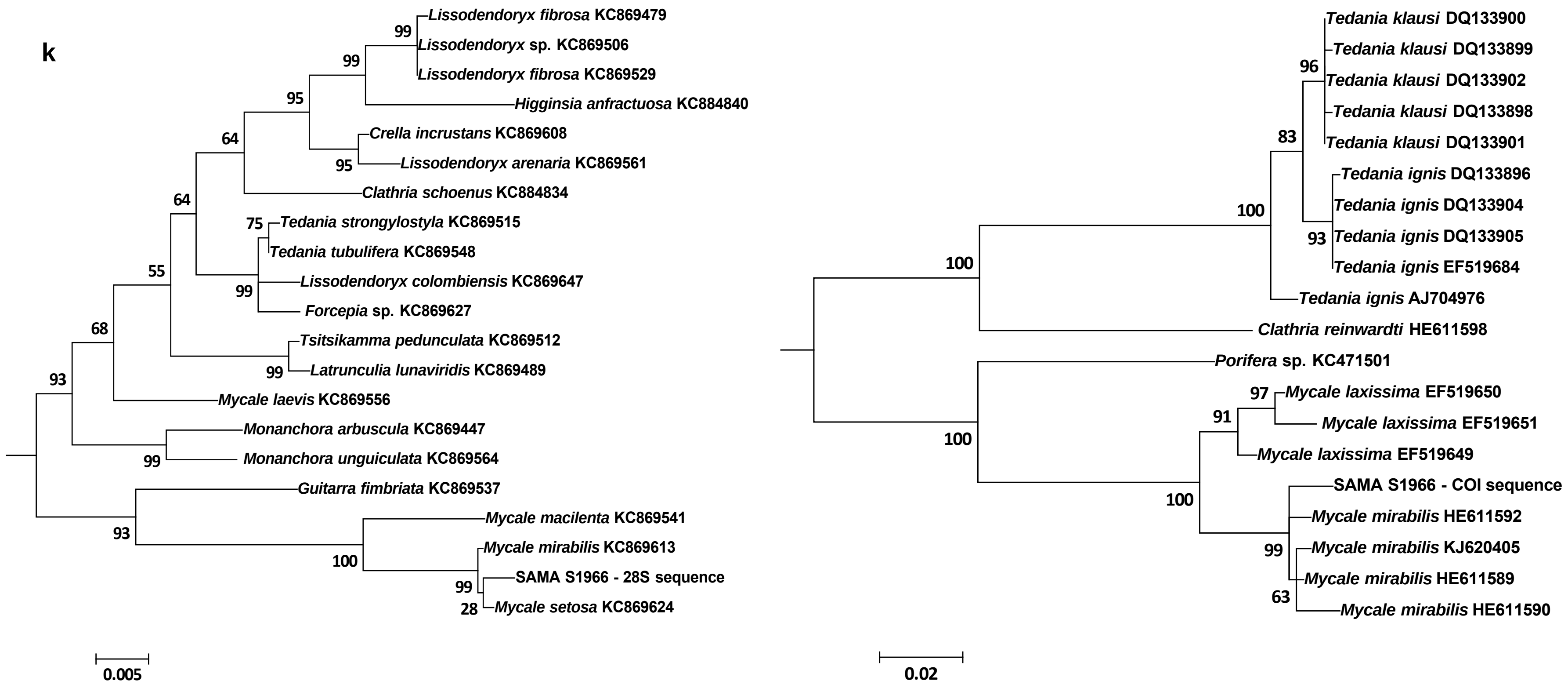
k
Lissodendoryx fibrosa KC869479
Lissodendoryx sp. KC869506
Lissodendoryx fibrosa KC869529
Higginsia anfractuosa KC884840
Crella incrustans KC869608
Lissodendoryx arenaria KC869561
Clathria schoenus KC884834
Tedania strongylostyla KC869515
Tedania tubulifera KC869548
Lissodendoryx colombiensis KC869647
Forcepia sp. KC869627
Tsitsikamma pedunculata KC869512
Latrunculia lunaviridis KC869489
Mycale laevis KC869556
Monanchora arbuscula KC869447
Monanchora unguiculata KC869564
Guitarra fimbriata KC869537
Mycale macilenta KC869541
Mycale mirabilis KC869613
SAMA S1966 - 28S sequence
Mycale setosa KC869624
0.005
Tedania klausi DQ133900
Tedania klausi DQ133899
Tedania klausi DQ133902
Tedania klausi DQ133898
Tedania klausi DQ133901
Tedania ignis DQ133896
Tedania ignis DQ133904
Tedania ignis DQ133905
Tedania ignis EF519684
Tedania ignis AJ704976
Clathria reinwardti HE611598
Porifera sp. KC471501
Mycale laxissima EF519650
Mycale laxissima EF519651
Mycale laxissima EF519649
SAMA S1966 - COI sequence
Mycale mirabilis HE611592
Mycale mirabilis KJ620405
Mycale mirabilis HE611589
Mycale mirabilis HE611590
0.02

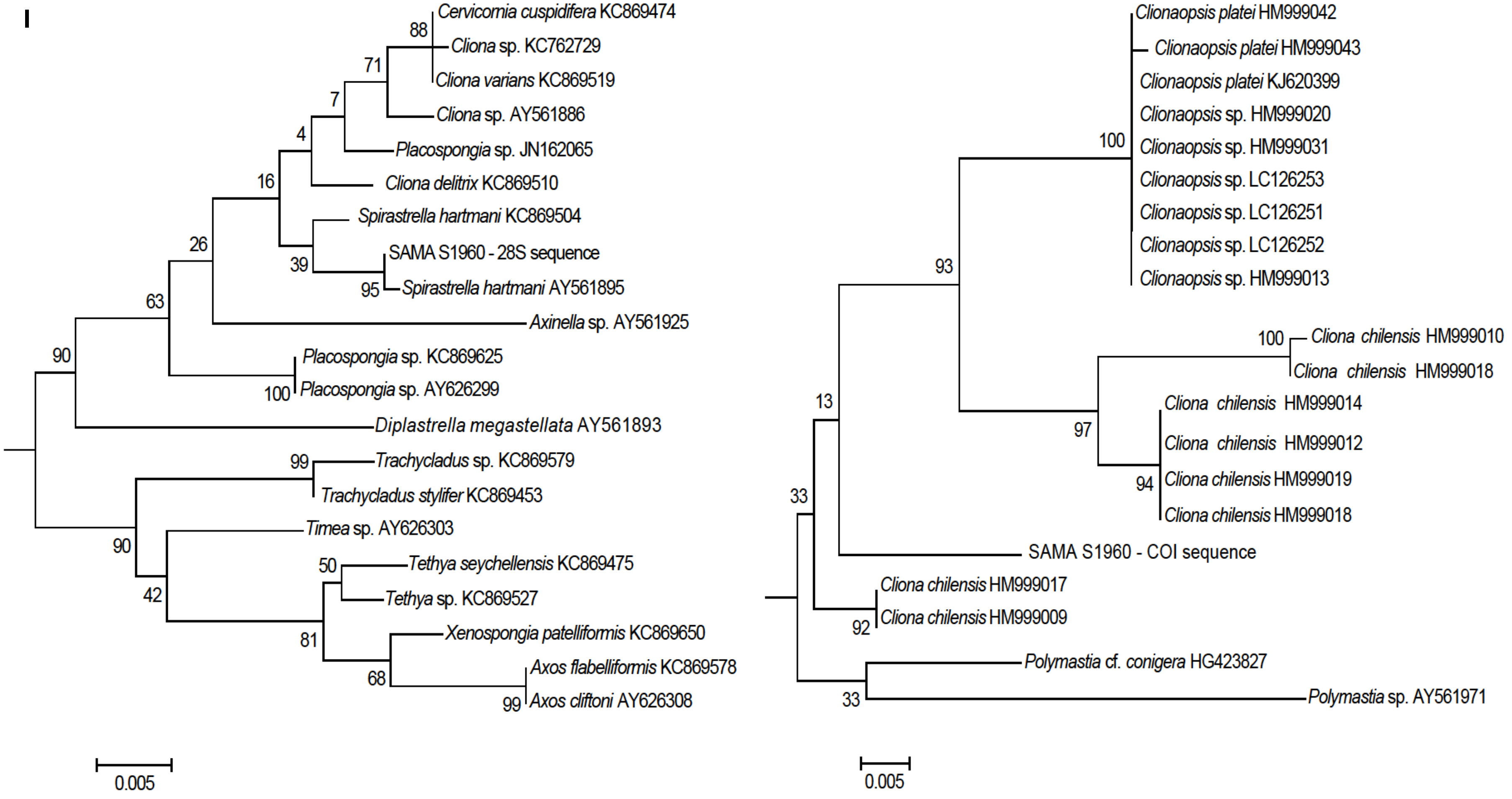
I
Cervicornia cuspidifera KC869474
Cliona sp. KC762729
Cliona varians KC869519
Cliona sp. AY561886
Placospongia sp. JN162065
Cliona delitrix KC869510
Spirastrella hartmani KC869504
SAMA S1960 - 28S sequence
Spirastrella hartmani AY561895
Axinella sp. AY561925
Placospongia sp. KC869625
Placospongia sp. AY626299
Diplastrella megastellata AY561893
Trachycladus sp. KC869579
Trachycladus stylifer KC869453
Timea sp. AY626303
Tethya seychellensis KC869475
Tethya sp. KC869527
Xenospongia patelliformis KC869650
Axos flabelliformis KC869578
Axos cliftoni AY626308
0.005
Clionaopsis platei HM999042
Clionaopsis platei HM999043
Clionaopsis platei KJ620399
Clionaopsis sp. HM999020
Clionaopsis sp. HM999031
Clionaopsis sp. LC126253
Clionaopsis sp. LC126251
Clionaopsis sp. LC126252
Clionaopsis sp. HM999013
Cliona chilensis HM999010
Cliona chilensis HM999018
Cliona chilensis HM999014
Cliona chilensis HM999012
Cliona chilensis HM999019
Cliona chilensis HM999018
SAMA S1960 - COI sequence
Cliona chilensis HM999017
Cliona chilensis HM999009
Polymastia cf. conigera HG423827
Polymastia sp. AY561971
0.005

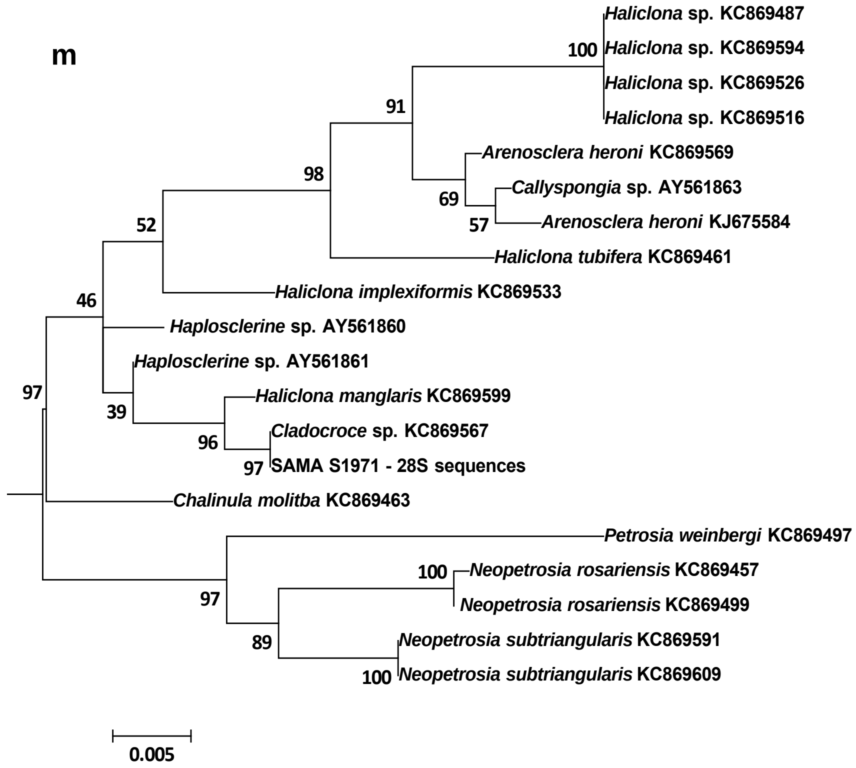
m
Haliclona sp. KC869487
Haliclona sp. KC869594
Haliclona sp. KC869526
Haliclona sp. KC869516
Arenosclera heroni KC869569
Callyspongia sp. AY561863
Arenosclera heroni KJ675584
Haliclona tubifera KC869461
Haliclona implexiformis KC869533
Haplosclerine sp. AY561860
Haplosclerine sp. AY561861
Haliclona manglaris KC869599
Cladocroce sp. KC869567
SAMA S1971 - 28S sequences
Chalinula molitba KC869463
Petrosia weinbergi KC869497
Neopetrosia rosariensis KC869457
Neopetrosia rosariensis KC869499
Neopetrosia subtriangularis KC869591
Neopetrosia subtriangularis KC869609
100
91
98
69
57
52
46
97
39
96
97
100
97
89
100
0.005
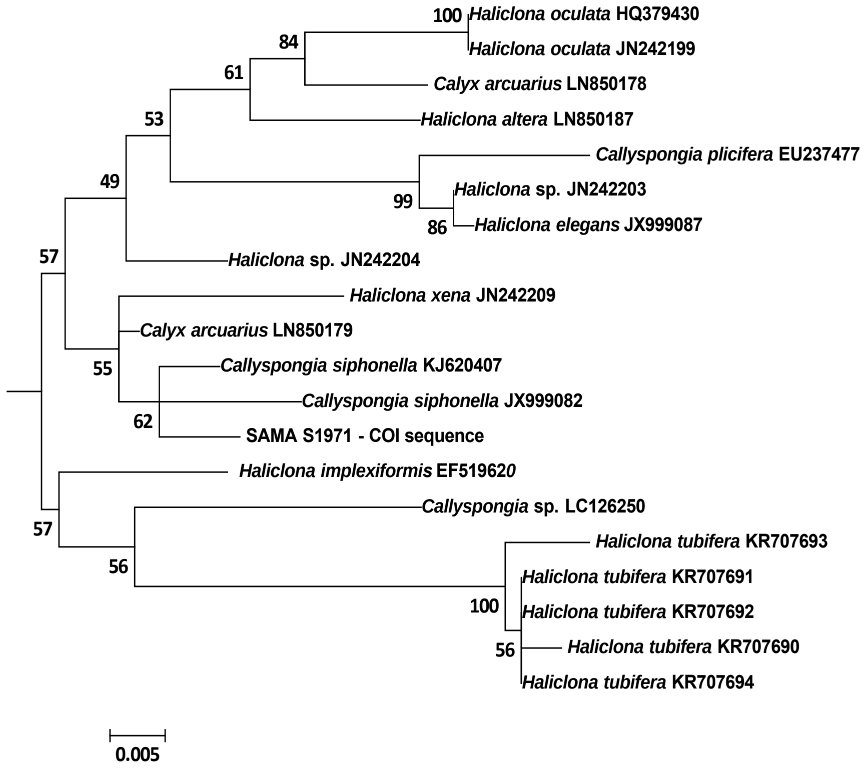
Haliclona oculata HQ379430
Haliclona oculata JN242199
Calyx arcuarius LN850178
Haliclona altera LN850187
Callyspongia plicifera EU237477
Haliclona sp. JN242203
Haliclona elegans JX999087
Haliclona sp. JN242204
Haliclona xena JN242209
Calyx arcuarius LN850179
Callyspongia siphonella KJ620407
Callyspongia siphonella JX999082
SAMA S1971 - COI sequence
Haliclona implexiformis EF519620
Callyspongia sp. LC126250
Haliclona tubifera KR707693
Haliclona tubifera KR707691
Haliclona tubifera KR707692
Haliclona tubifera KR707690
Haliclona tubifera KR707694
100
84
61
53
49
99
86
57
55
62
57
56
100
56
0.005

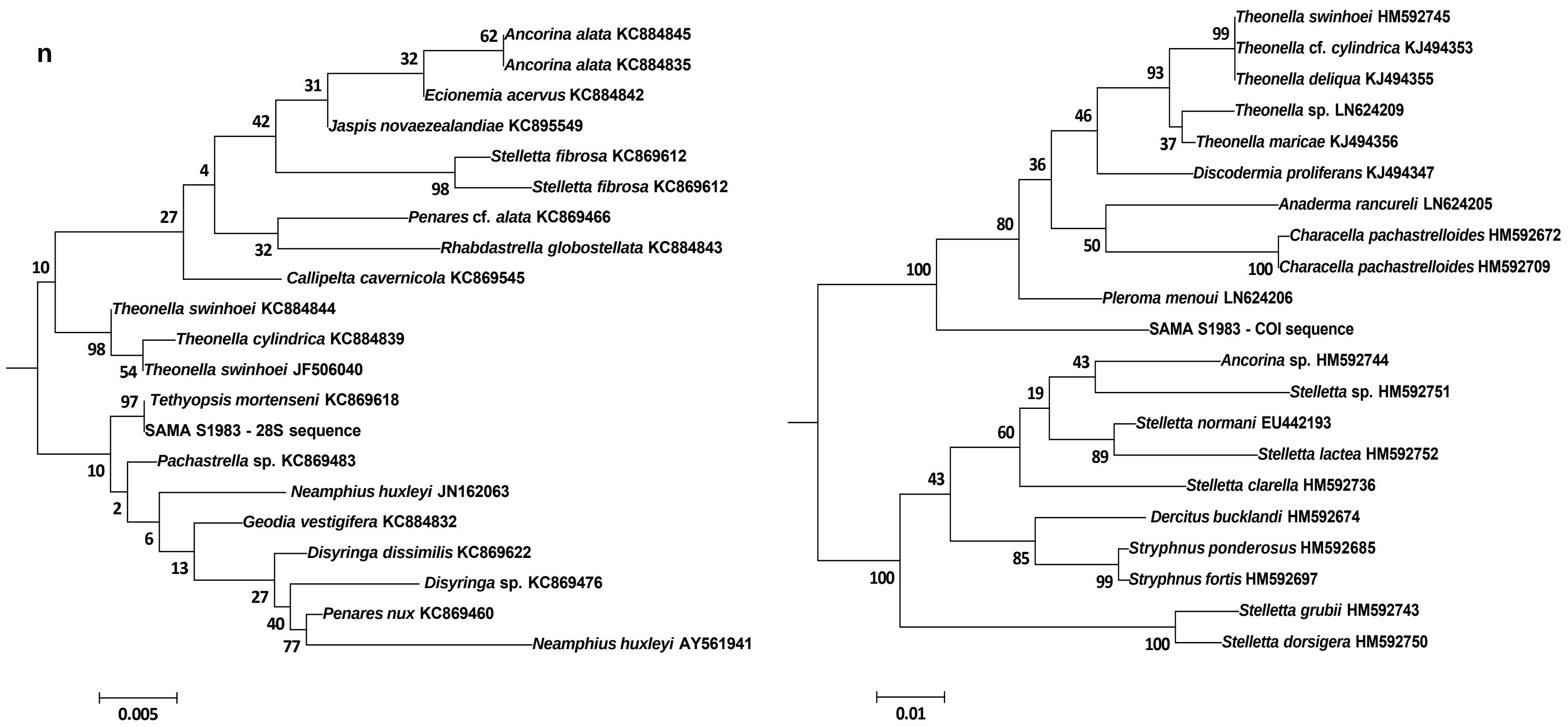

Supplementary Figure S1. Phylogenetic relationship of fourteen sponges using Maximum Likelihood method based on 28S rRNA gene and COI mtDNA. **a.** Phylogenetic relationship of sponge SAMA S1981. **b.** Phylogenetic relationship of sponge SAMA S1963. **c.** Phylogenetic relationship of sponge SAMA S1965. **d.** Phylogenetic relationship of sponge SAMA S1973. **e.** Phylogenetic relationship of sponge SAMA S1989. **f.** Phylogenetic relationship of sponge SAMA S1961. **g.** Phylogenetic relationship of sponge SAMA S1962. **h.** Phylogenetic relationship of sponge SAMA S1991. **i.** Phylogenetic relationship of sponge SAMA S1982. **j.** Phylogenetic relationship of sponge SAMA S1994. **k.** Phylogenetic relationship of sponge SAMA S1966. **l.** Phylogenetic relationship of sponge SAMA S1960. **m.** Phylogenetic relationship of sponge SAMA S1971. **n.** Phylogenetic relationship of sponge SAMA S1983.

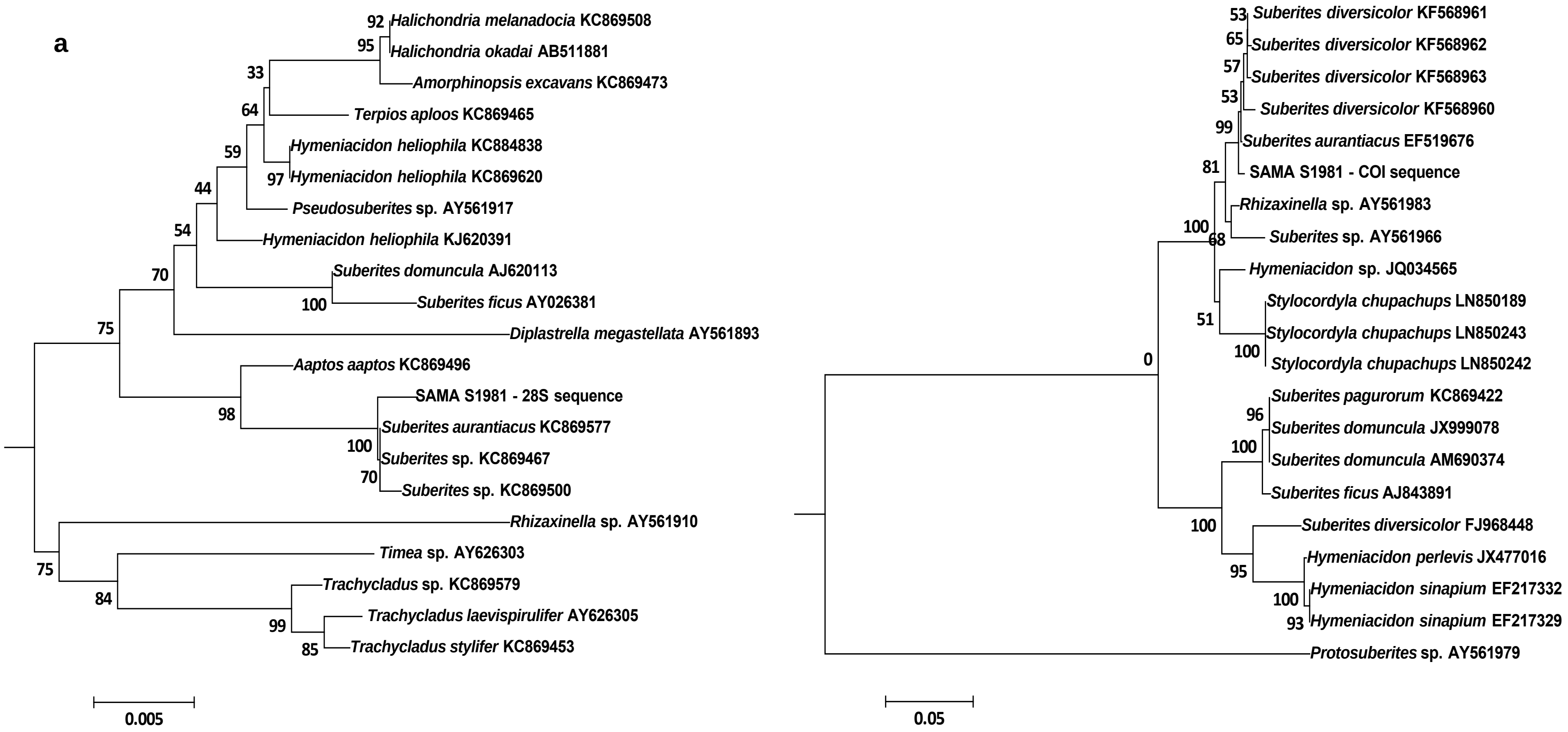
a
Halichondria melanadocia KC869508
Halichondria okadai AB511881
Amorphinopsis excavans KC869473
Terpios aploos KC869465
Hymeniacidon heliophila KC884838
Hymeniacidon heliophila KC869620
Pseudosuberites sp. AY561917
Hymeniacidon heliophila KJ620391
Suberites domuncula AJ620113
Suberites ficus AY026381
Diplastrella megastellata AY561893
Aaptos aaptos KC869496
SAMA S1981 - 28S sequence
Suberites aurantiacus KC869577
Suberites sp. KC869467
Suberites sp. KC869500
Rhizaxinella sp. AY561910
Timea sp. AY626303
Trachycladus sp. KC869579
Trachycladus laevispirulifer AY626305
Trachycladus stylifer KC869453
0.005
Suberites diversicolor KF568961
Suberites diversicolor KF568962
Suberites diversicolor KF568963
Suberites diversicolor KF568960
Suberites aurantiacus EF519676
SAMA S1981 - COI sequence
Rhizaxinella sp. AY561983
Suberites sp. AY561966
Hymeniacidon sp. JQ034565
Stylocordyla chupachups LN850189
Stylocordyla chupachups LN850243
Stylocordyla chupachups LN850242
Suberites pagurorum KC869422
Suberites domuncula JX999078
Suberites domuncula AM690374
Suberites ficus AJ843891
Suberites diversicolor FJ968448
Hymeniacidon perlevis JX477016
Hymeniacidon sinapium EF217332
Hymeniacidon sinapium EF217329
Protosuberites sp. AY561979
0.05

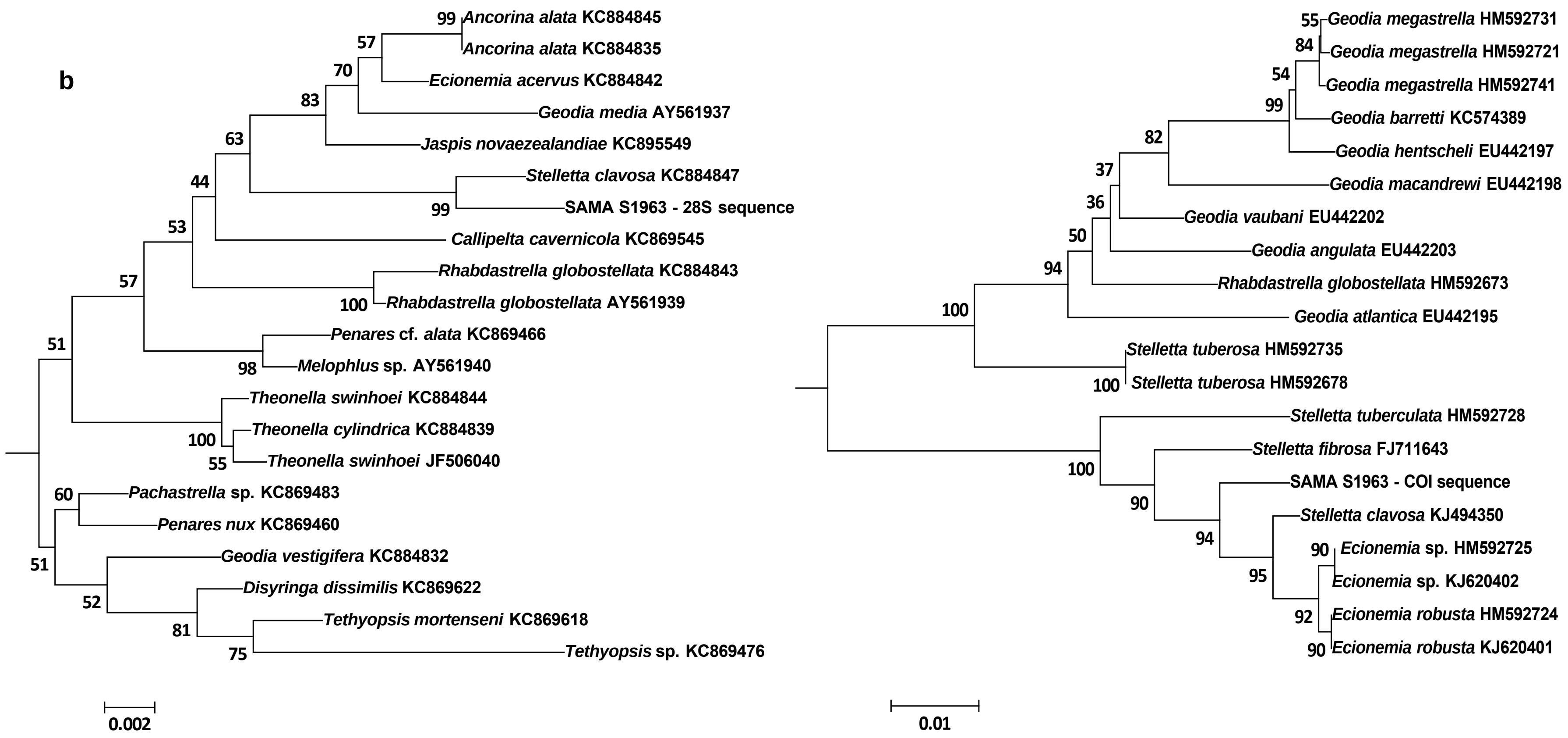
b
Ancorina alata KC884845
Ancorina alata KC884835
Ecionemia acervus KC884842
Geodia media AY561937
Jaspis novaezealandiae KC895549
Stelletta clavosa KC884847
SAMA S1963 - 28S sequence
Callipelta cavernicola KC869545
Rhabdastrella globostellata KC884843
Rhabdastrella globostellata AY561939
Penares cf. alata KC869466
Melophlus sp. AY561940
Theonella swinhoei KC884844
Theonella cylindrica KC884839
Theonella swinhoei JF506040
Pachastrella sp. KC869483
Penares nux KC869460
Geodia vestigifera KC884832
Disyringa dissimilis KC869622
Tethyopsis mortenseni KC869618
Tethyopsis sp. KC869476
0.002
Geodia megastrella HM592731
Geodia megastrella HM592721
Geodia megastrella HM592741
Geodia barretti KC574389
Geodia hentscheli EU442197
Geodia macandrewi EU442198
Geodia vaubani EU442202
Geodia angulata EU442203
Rhabdastrella globostellata HM592673
Geodia atlantica EU442195
Stelletta tuberosa HM592735
Stelletta tuberosa HM592678
Stelletta tuberculata HM592728
Stelletta fibrosa FJ711643
SAMA S1963 - COI sequence
Stelletta clavosa KJ494350
Ecionemia sp. HM592725
Ecionemia sp. KJ620402
Ecionemia robusta HM592724
Ecionemia robusta KJ620401
0.01

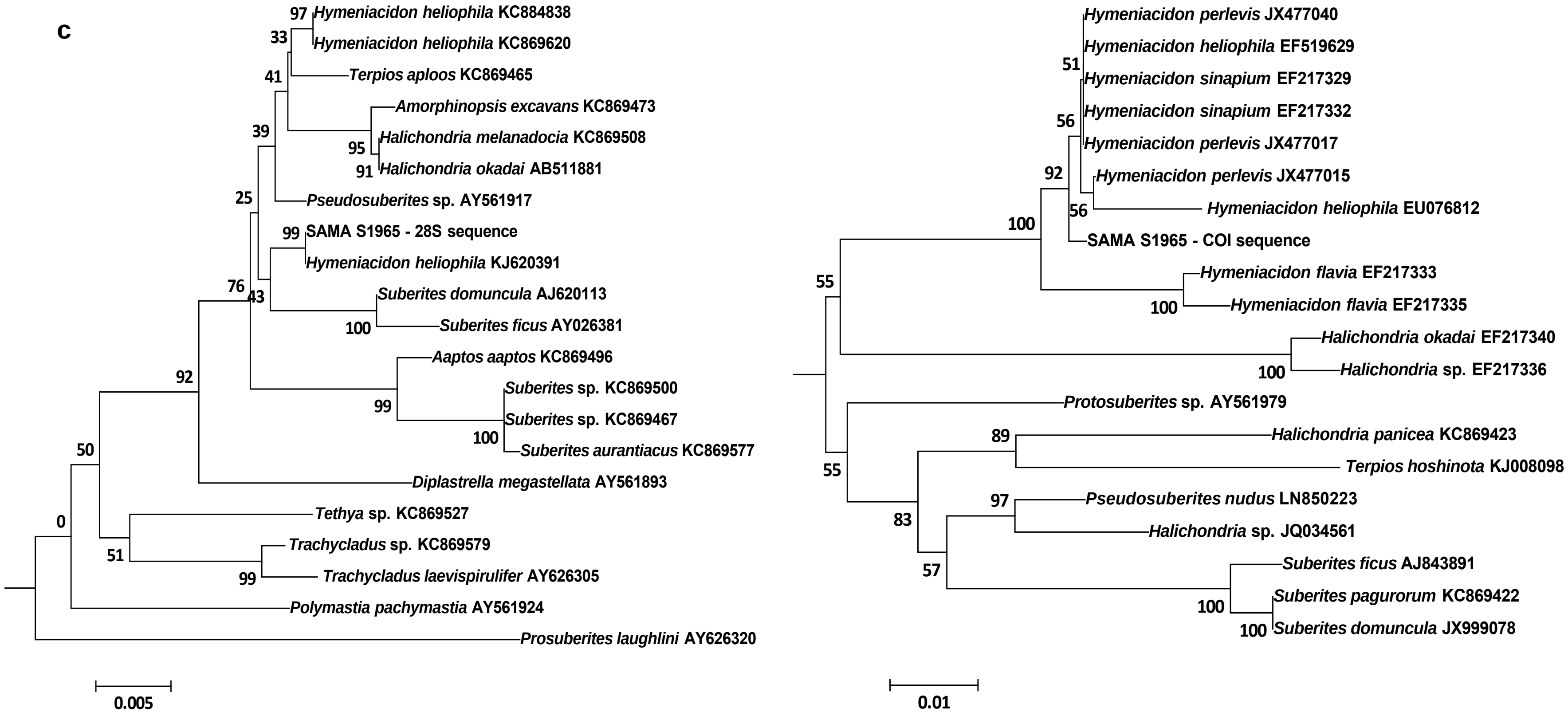
c
Hymeniacidon heliophila KC884838
Hymeniacidon heliophila KC869620
Terpios aploos KC869465
Amorphinopsis excavans KC869473
Halichondria melanadocia KC869508
Halichondria okadai AB511881
Pseudosuberites sp. AY561917
SAMA S1965 - 28S sequence
Hymeniacidon heliophila KJ620391
Suberites domuncula AJ620113
Suberites ficus AY026381
Aaptos aaptos KC869496
Suberites sp. KC869500
Suberites sp. KC869467
Suberites aurantiacus KC869577
Diplastrella megastellata AY561893
Tethya sp. KC869527
Trachycladus sp. KC869579
Trachycladus laevispirulifer AY626305
Polymastia pachymastia AY561924
Prosuberites laughlini AY626320
0.005
Hymeniacidon perlevis JX477040
Hymeniacidon heliophila EF519629
Hymeniacidon sinapium EF217329
Hymeniacidon sinapium EF217332
Hymeniacidon perlevis JX477017
Hymeniacidon perlevis JX477015
Hymeniacidon heliophila EU076812
SAMA S1965 - COI sequence
Hymeniacidon flavia EF217333
Hymeniacidon flavia EF217335
Halichondria okadai EF217340
Halichondria sp. EF217336
Protosuberites sp. AY561979
Halichondria panicea KC869423
Terpios hoshinota KJ008098
Pseudosuberites nudus LN850223
Halichondria sp. JQ034561
Suberites ficus AJ843891
Suberites pagurorum KC869422
Suberites domuncula JX999078
0.01

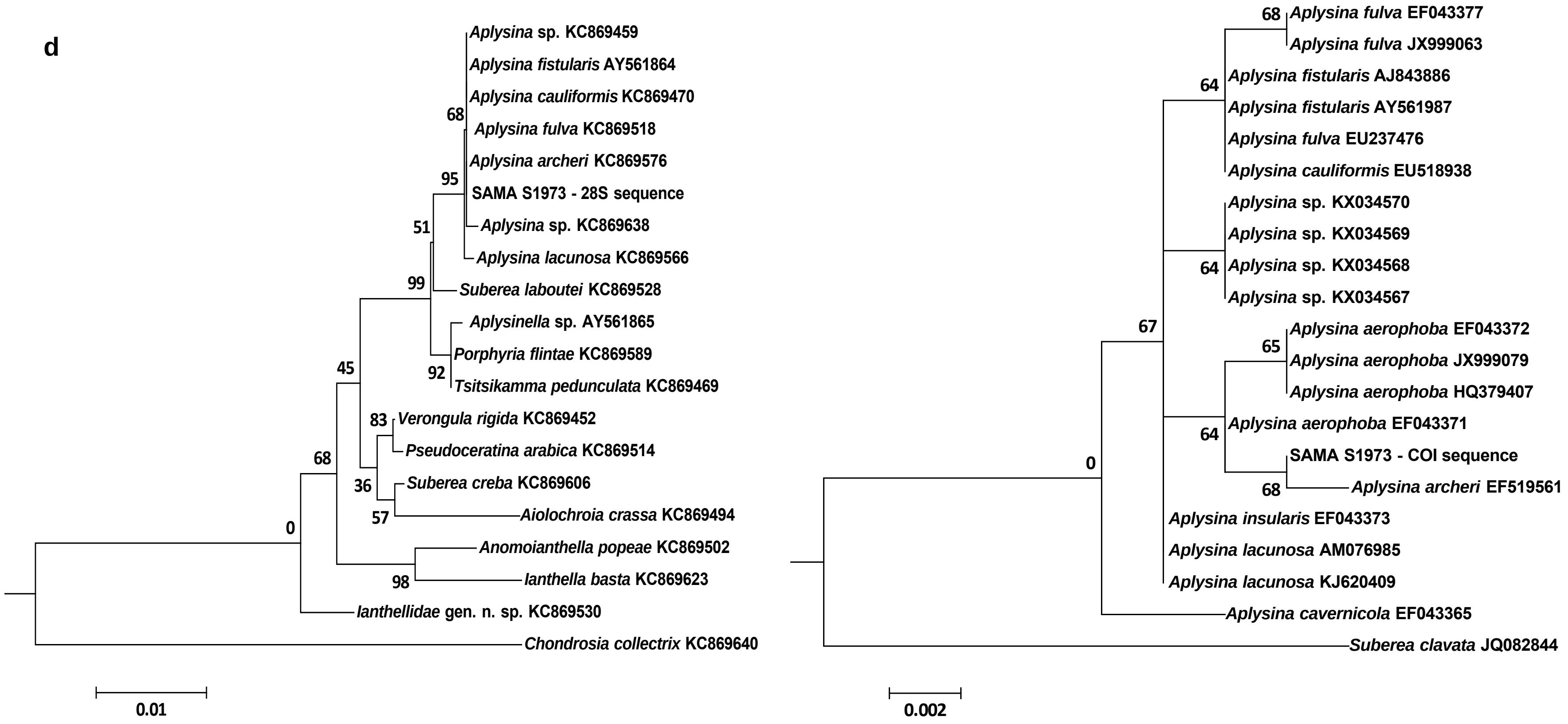
d
Aplysina sp. KC869459
Aplysina fistularis AY561864
Aplysina cauliformis KC869470
Aplysina fulva KC869518
Aplysina archeri KC869576
SAMA S1973 - 28S sequence
Aplysina sp. KC869638
Aplysina lacunosa KC869566
Suberea laboutei KC869528
Aplysinella sp. AY561865
Porphyria flintae KC869589
Tsitsikamma pedunculata KC869469
Verongula rigida KC869452
Pseudoceratina arabica KC869514
Suberea creba KC869606
Aiolochroia crassa KC869494
Anomoianthella popeae KC869502
Ianthella basta KC869623
Ianthellidae gen. n. sp. KC869530
Chondrosia collectrix KC869640
0.01
Aplysina fulva EF043377
Aplysina fulva JX999063
Aplysina fistularis AJ843886
Aplysina fistularis AY561987
Aplysina fulva EU237476
Aplysina cauliformis EU518938
Aplysina sp. KX034570
Aplysina sp. KX034569
Aplysina sp. KX034568
Aplysina sp. KX034567
Aplysina aerophoba EF043372
Aplysina aerophoba JX999079
Aplysina aerophoba HQ379407
Aplysina aerophoba EF043371
SAMA S1973 - COI sequence
Aplysina archeri EF519561
Aplysina insularis EF043373
Aplysina lacunosa AM076985
Aplysina lacunosa KJ620409
Aplysina cavernicola EF043365
Suberea clavata JQ082844
0.002

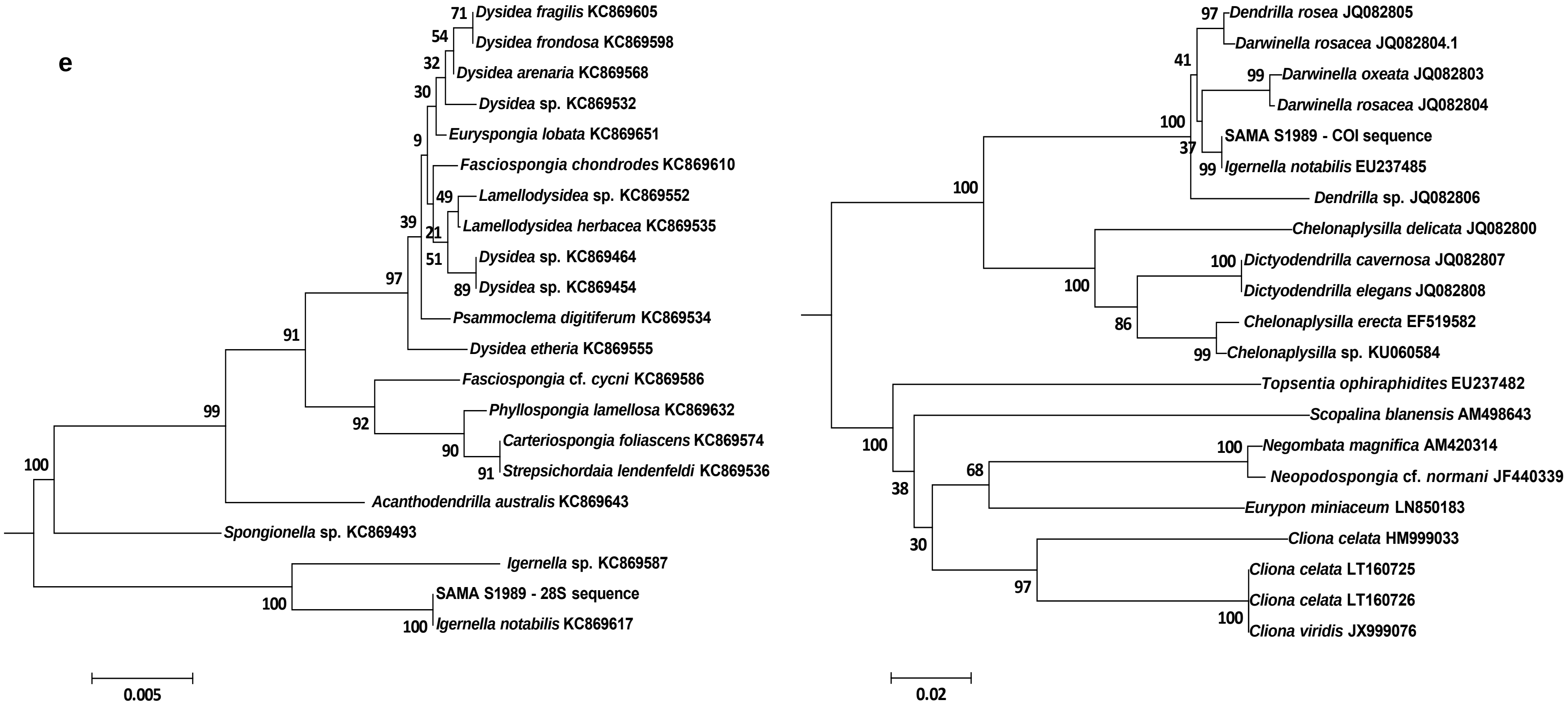
e
Dysidea fragilis KC869605
Dysidea frondosa KC869598
Dysidea arenaria KC869568
Dysidea sp. KC869532
Euryspongia lobata KC869651
Fasciospongia chondrodes KC869610
Lamellodysidea sp. KC869552
Lamellodysidea herbacea KC869535
Dysidea sp. KC869464
Dysidea sp. KC869454
Psammoclema digitiferum KC869534
Dysidea etheria KC869555
Fasciospongia cf. cycni KC869586
Phyllospongia lamellosa KC869632
Carteriospongia foliascens KC869574
Strepsichordaia lendenfeldi KC869536
Acanthodendrilla australis KC869643
Spongionella sp. KC869493
Igernella sp. KC869587
SAMA S1989 - 28S sequence
Igernella notabilis KC869617
0.005
Dendrilla rosea JQ082805
Darwinella rosacea JQ082804.1
Darwinella oxeata JQ082803
Darwinella rosacea JQ082804
SAMA S1989 - COI sequence
Igernella notabilis EU237485
Dendrilla sp. JQ082806
Chelonaplysilla delicata JQ082800
Dictyodendrilla cavernosa JQ082807
Dictyodendrilla elegans JQ082808
Chelonaplysilla erecta EF519582
Chelonaplysilla sp. KU060584
Topsentia ophiraphidites EU237482
Scopalina blanensis AM498643
Negombata magnifica AM420314
Neopodospongia cf. normani JF440339
Eurypon miniaceum LN850183
Cliona celata HM999033
Cliona celata LT160725
Cliona celata LT160726
Cliona viridis JX999076
0.02

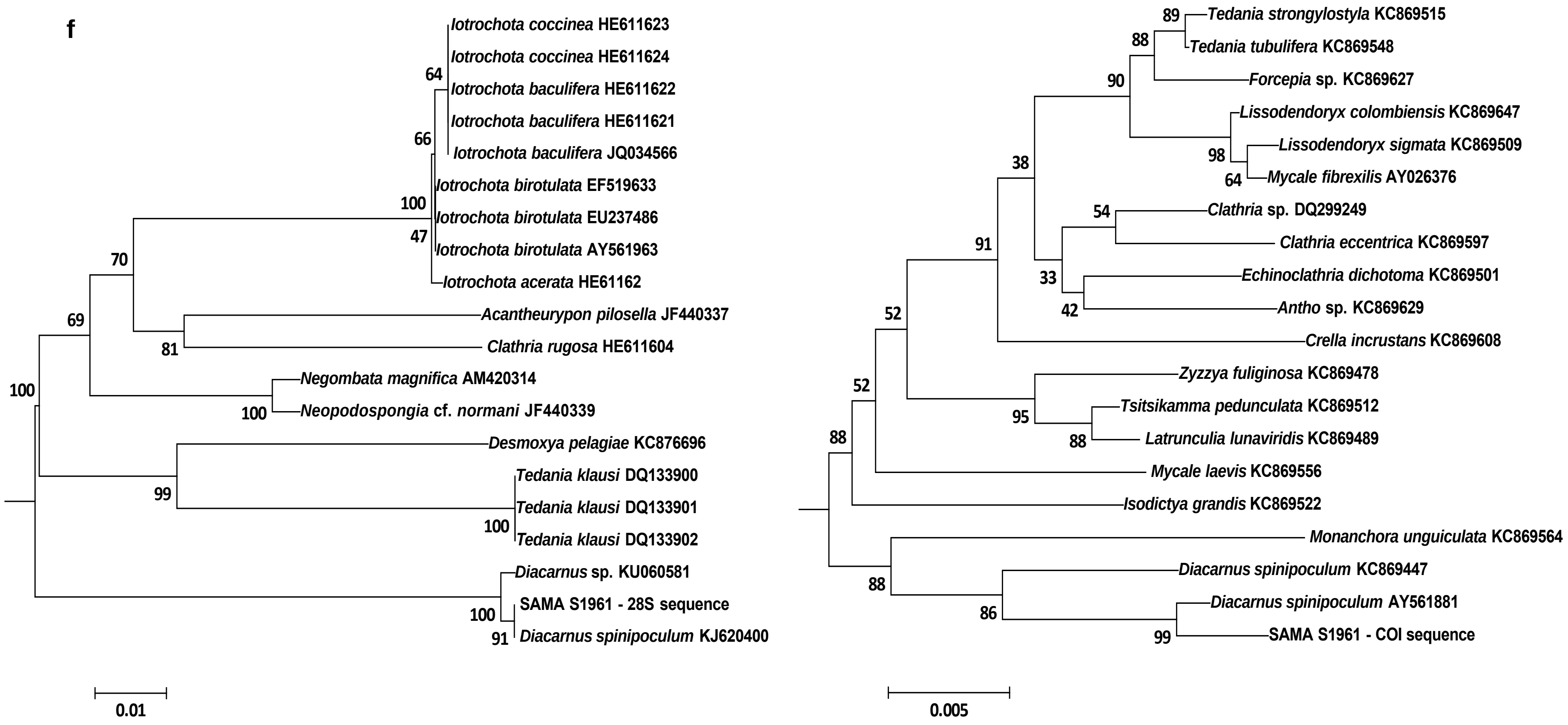
f
Iotrochota coccinea HE611623
Iotrochota coccinea HE611624
Iotrochota baculifera HE611622
Iotrochota baculifera HE611621
Iotrochota baculifera JQ034566
Iotrochota birotulata EF519633
Iotrochota birotulata EU237486
Iotrochota birotulata AY561963
Iotrochota acerata HE61162
Acantheurypon pilosella JF440337
Clathria rugosa HE611604
Negombata magnifica AM420314
Neopodospongia cf. normani JF440339
Desmoxya pelagiae KC876696
Tedania klausi DQ133900
Tedania klausi DQ133901
Tedania klausi DQ133902
Diacarnus sp. KU060581
SAMA S1961 - 28S sequence
Diacarnus spinipoculum KJ620400
64
66
100
47
70
69
81
100
100
99
100
100
91
0.01
Tedania strongylostyla KC869515
Tedania tubulifera KC869548
Forcepia sp. KC869627
Lissodendoryx colombiensis KC869647
Lissodendoryx sigmata KC869509
Mycale fibrexilis AY026376
Clathria sp. DQ299249
Clathria eccentrica KC869597
Echinoclathria dichotoma KC869501
Antho sp. KC869629
Crella incrustans KC869608
Zyzzya fuliginosa KC869478
Tsitsikamma pedunculata KC869512
Latrunculia lunaviridis KC869489
Mycale laevis KC869556
Isodictya grandis KC869522
Monanchora unguiculata KC869564
Diacarnus spinipoculum KC869447
Diacarnus spinipoculum AY561881
SAMA S1961 - COI sequence
89
88
90
98
64
38
54
91
33
42
52
52
95
88
88
88
86
99
0.005

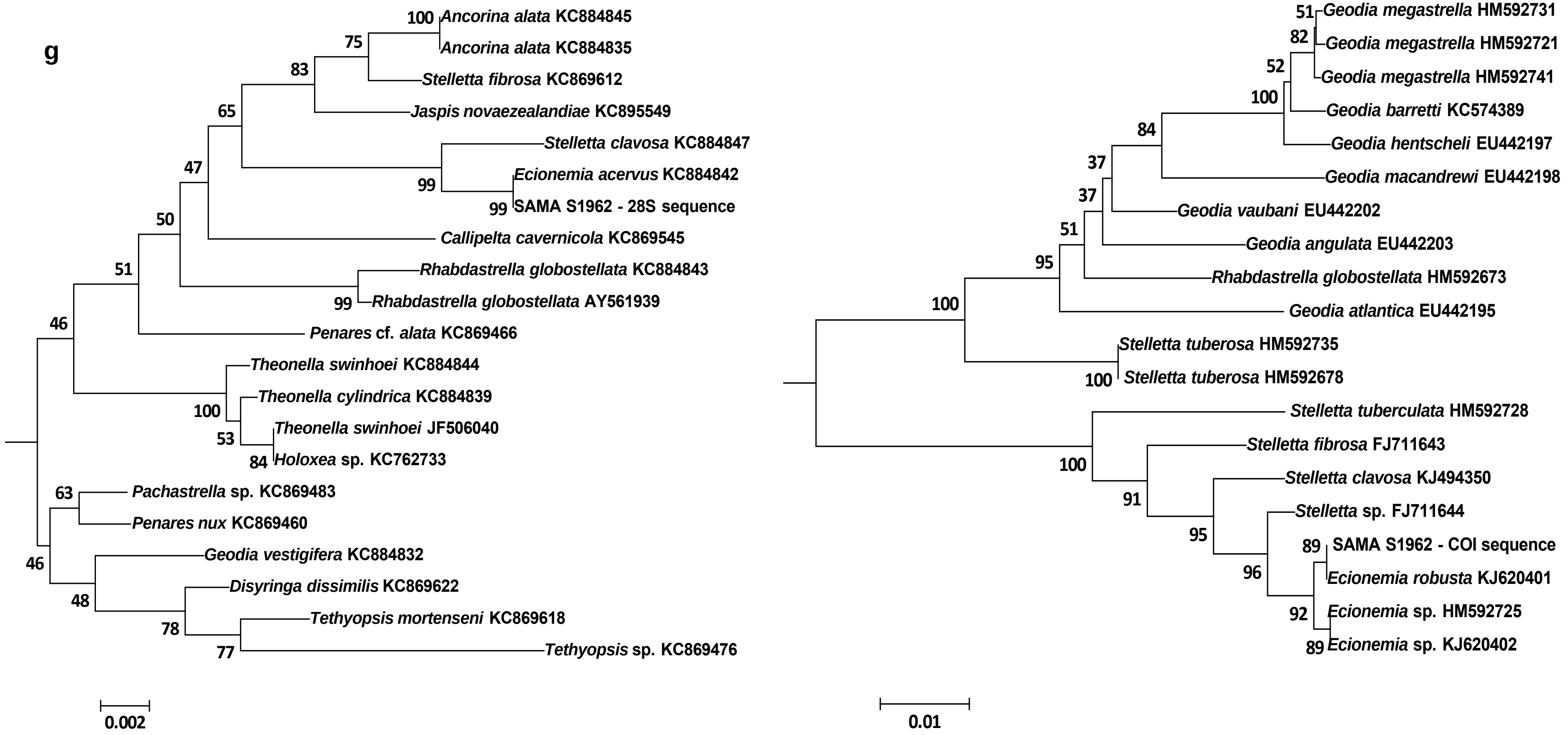
g
Ancorina alata KC884845
Ancorina alata KC884835
Stelletta fibrosa KC869612
Jaspis novaezealandiae KC895549
Stelletta clavosa KC884847
Ecionemia acervus KC884842
SAMA S1962 - 28S sequence
Callipelta cavernicola KC869545
Rhabdastrella globostellata KC884843
Rhabdastrella globostellata AY561939
Penares cf. alata KC869466
Theonella swinhoei KC884844
Theonella cylindrica KC884839
Theonella swinhoei JF506040
Holoxea sp. KC762733
Pachastrella sp. KC869483
Penares nux KC869460
Geodia vestigifera KC884832
Disyringa dissimilis KC869622
Tethyopsis mortenseni KC869618
Tethyopsis sp. KC869476
0.002
Geodia megastrella HM592731
Geodia megastrella HM592721
Geodia megastrella HM592741
Geodia barretti KC574389
Geodia hentscheli EU442197
Geodia macandrewi EU442198
Geodia vaubani EU442202
Geodia angulata EU442203
Rhabdastrella globostellata HM592673
Geodia atlantica EU442195
Stelletta tuberosa HM592735
Stelletta tuberosa HM592678
Stelletta tuberculata HM592728
Stelletta fibrosa FJ711643
Stelletta clavosa KJ494350
Stelletta sp. FJ711644
SAMA S1962 - COI sequence
Ecionemia robusta KJ620401
Ecionemia sp. HM592725
Ecionemia sp. KJ620402
0.01

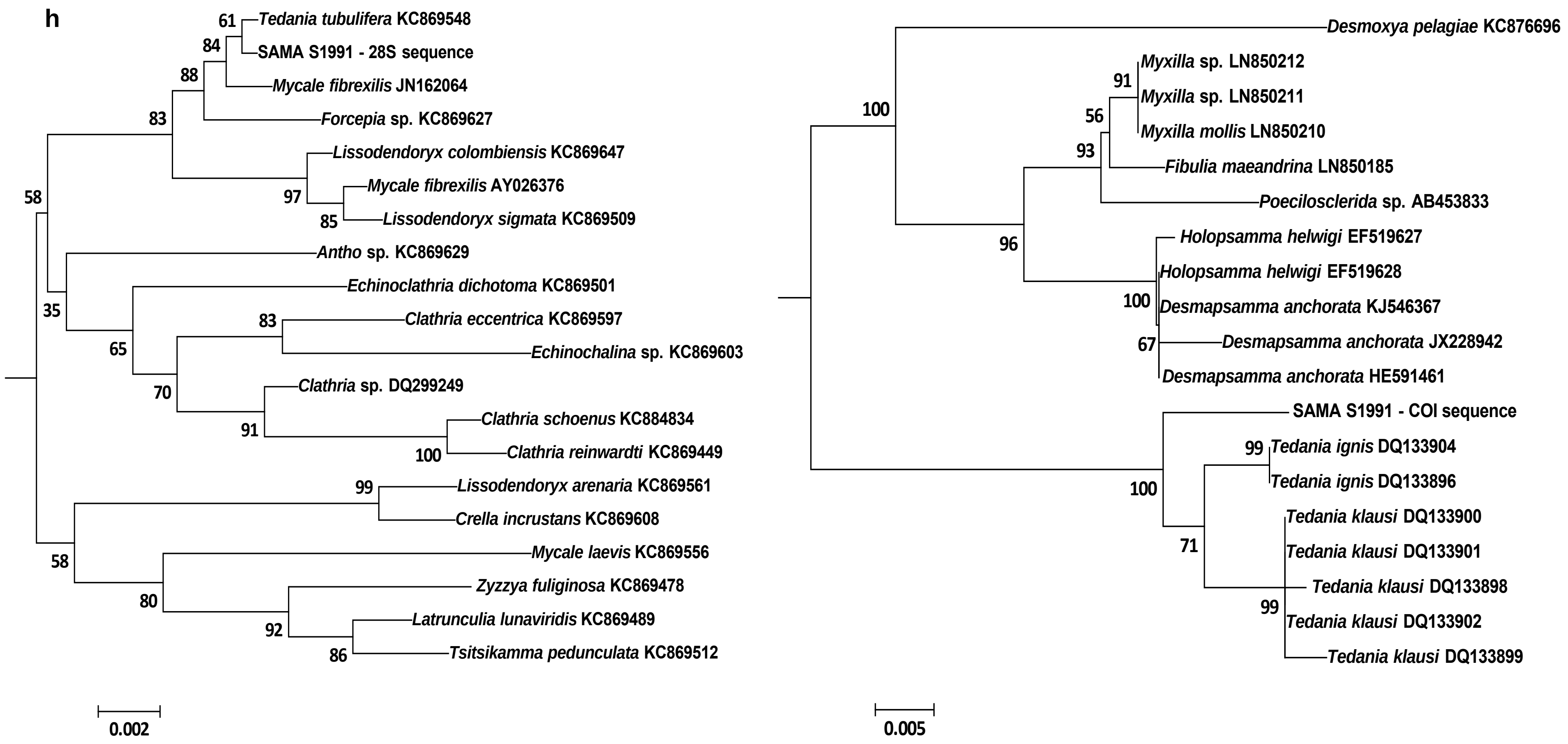

h
Tedania tubulifera KC869548
SAMA S1991 - 28S sequence
Mycale fibrexilis JN162064
Forcepia sp. KC869627
Lissodendoryx colombiensis KC869647
Mycale fibrexilis AY026376
Lissodendoryx sigmata KC869509
Antho sp. KC869629
Echinoclathria dichotoma KC869501
Clathria eccentrica KC869597
Echinochalina sp. KC869603
Clathria sp. DQ299249
Clathria schoenus KC884834
Clathria reinwardti KC869449
Lissodendoryx arenaria KC869561
Crella incrustans KC869608
Mycale laevis KC869556
Zyzzya fuliginosa KC869478
Latrunculia lunaviridis KC869489
Tsitsikamma pedunculata KC869512
0.002
Desmoxya pelagiae KC876696
Myxilla sp. LN850212
Myxilla sp. LN850211
Myxilla mollis LN850210
Fibulia maeandrina LN850185
Poecilosclerida sp. AB453833
Holopsamma helwigi EF519627
Holopsamma helwigi EF519628
Desmapsamma anchorata KJ546367
Desmapsamma anchorata JX228942
Desmapsamma anchorata HE591461
SAMA S1991 - COI sequence
Tedania ignis DQ133904
Tedania ignis DQ133896
Tedania klausi DQ133900
Tedania klausi DQ133901
Tedania klausi DQ133898
Tedania klausi DQ133902
Tedania klausi DQ133899
0.005

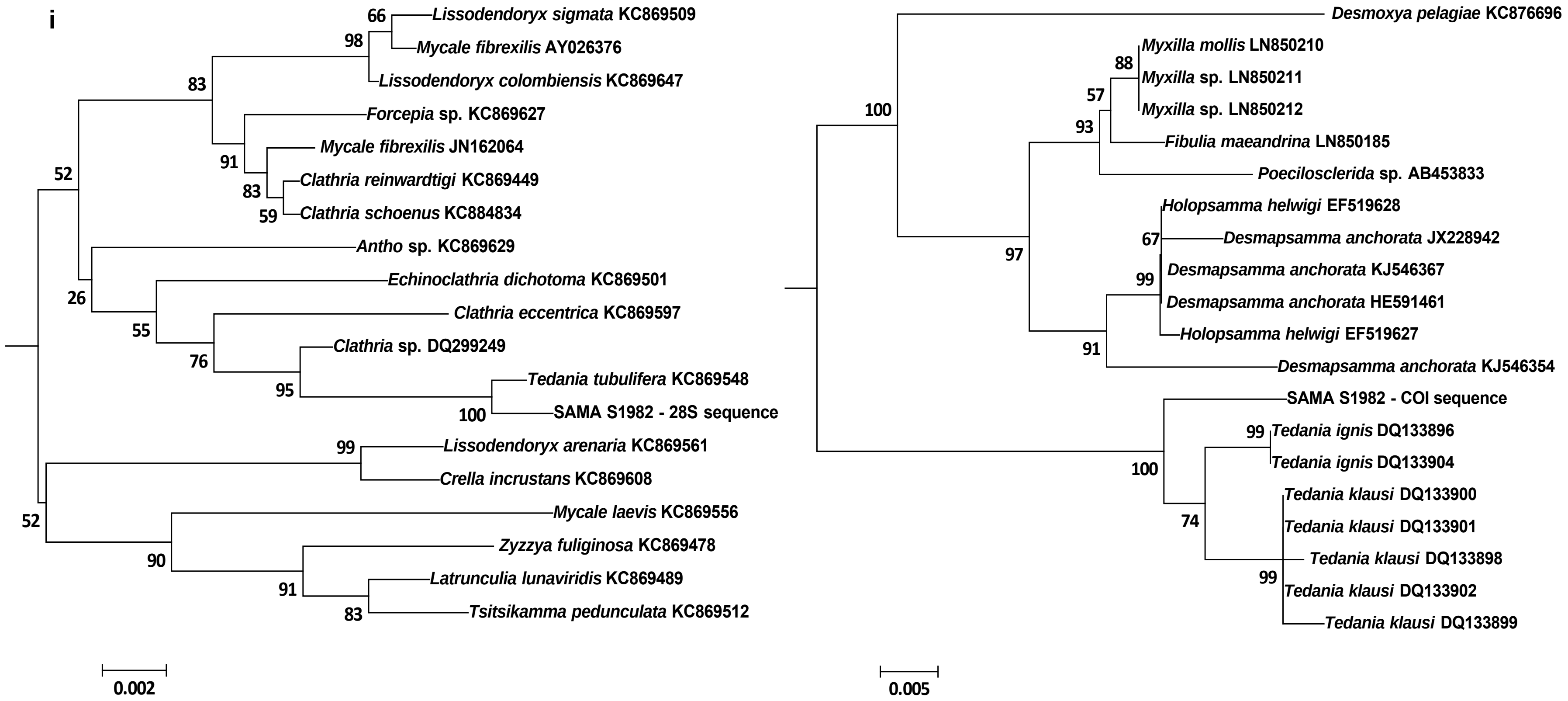
i
Lissodendoryx sigmata KC869509
Mycale fibrexilis AY026376
Lissodendoryx colombiensis KC869647
Forcepia sp. KC869627
Mycale fibrexilis JN162064
Clathria reinwardtigi KC869449
Clathria schoenus KC884834
Antho sp. KC869629
Echinoclathria dichotoma KC869501
Clathria eccentrica KC869597
Clathria sp. DQ299249
Tedania tubulifera KC869548
SAMA S1982 - 28S sequence
Lissodendoryx arenaria KC869561
Crella incrustans KC869608
Mycale laevis KC869556
Zyzzya fuliginosa KC869478
Latrunculia lunaviridis KC869489
Tsitsikamma pedunculata KC869512
0.002
Desmoxya pelagiae KC876696
Myxilla mollis LN850210
Myxilla sp. LN850211
Myxilla sp. LN850212
Fibulia maeandrina LN850185
Poecilosclerida sp. AB453833
Holopsamma helwigi EF519628
Desmapsamma anchorata JX228942
Desmapsamma anchorata KJ546367
Desmapsamma anchorata HE591461
Holopsamma helwigi EF519627
Desmapsamma anchorata KJ546354
SAMA S1982 - COI sequence
Tedania ignis DQ133896
Tedania ignis DQ133904
Tedania klausi DQ133900
Tedania klausi DQ133901
Tedania klausi DQ133898
Tedania klausi DQ133902
Tedania klausi DQ133899
0.005

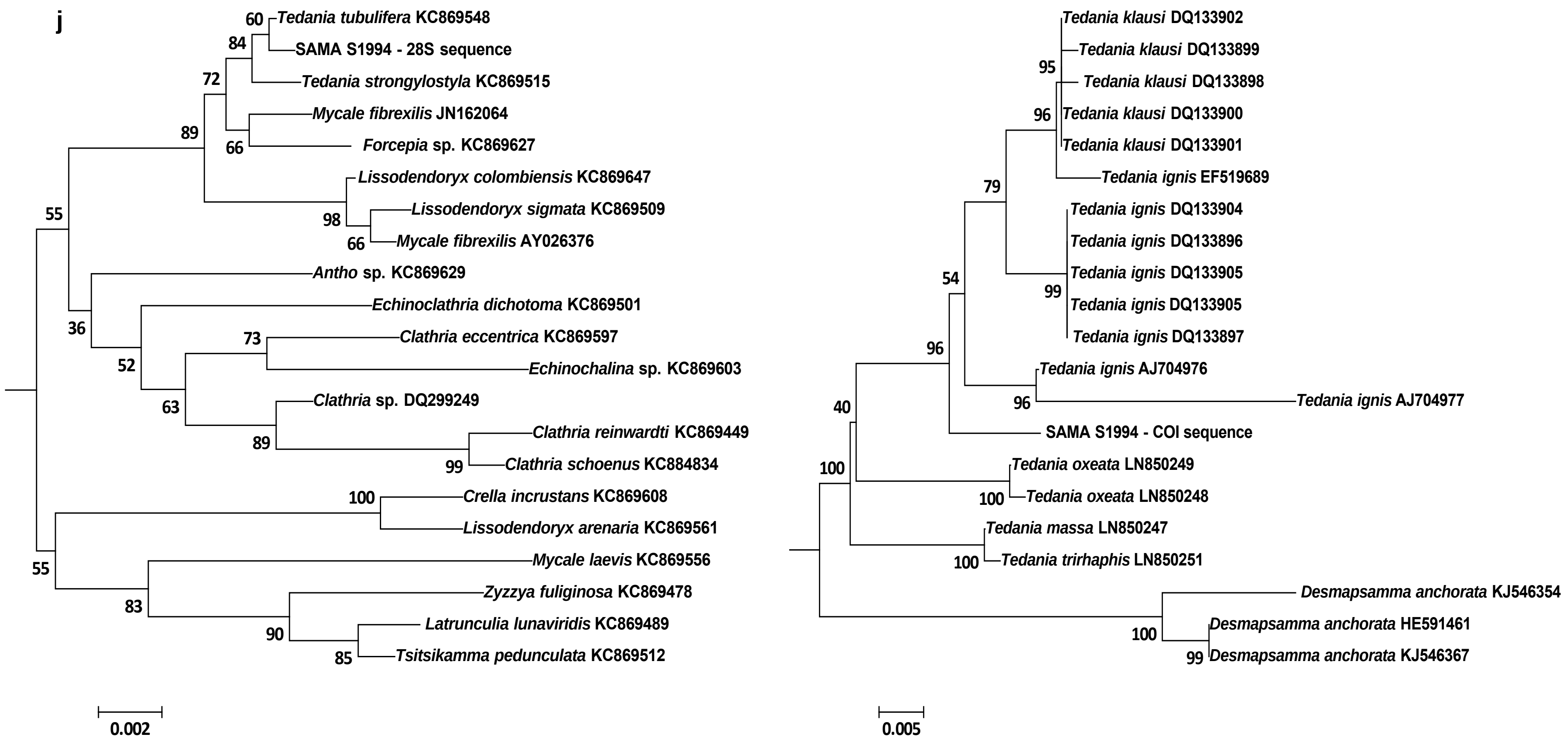

j
60
84
72
89
66
98
66
55
36
52
73
63
89
99
100
55
83
90
85
Tedania tubulifera KC869548
SAMA S1994 - 28S sequence
Tedania strongylostyla KC869515
Mycale fibrexilis JN162064
Forcepia sp. KC869627
Lissodendoryx colombiensis KC869647
Lissodendoryx sigmata KC869509
Mycale fibrexilis AY026376
Antho sp. KC869629
Echinoclathria dichotoma KC869501
Clathria eccentrica KC869597
Echinochalina sp. KC869603
Clathria sp. DQ299249
Clathria reinwardti KC869449
Clathria schoenus KC884834
Crella incrustans KC869608
Lissodendoryx arenaria KC869561
Mycale laevis KC869556
Zyzzya fuliginosa KC869478
Latrunculia lunaviridis KC869489
Tsitsikamma pedunculata KC869512
0.002
Tedania klausi DQ133902
Tedania klausi DQ133899
Tedania klausi DQ133898
Tedania klausi DQ133900
Tedania klausi DQ133901
Tedania ignis EF519689
Tedania ignis DQ133904
Tedania ignis DQ133896
Tedania ignis DQ133905
Tedania ignis DQ133905
Tedania ignis DQ133897
Tedania ignis AJ704976
Tedania ignis AJ704977
SAMA S1994 - COI sequence
Tedania oxeata LN850249
Tedania oxeata LN850248
Tedania massa LN850247
Tedania trirhaphis LN850251
Desmapsamma anchorata KJ546354
Desmapsamma anchorata HE591461
Desmapsamma anchorata KJ546367
95
96
79
99
54
96
96
40
100
100
100
100
99
0.005

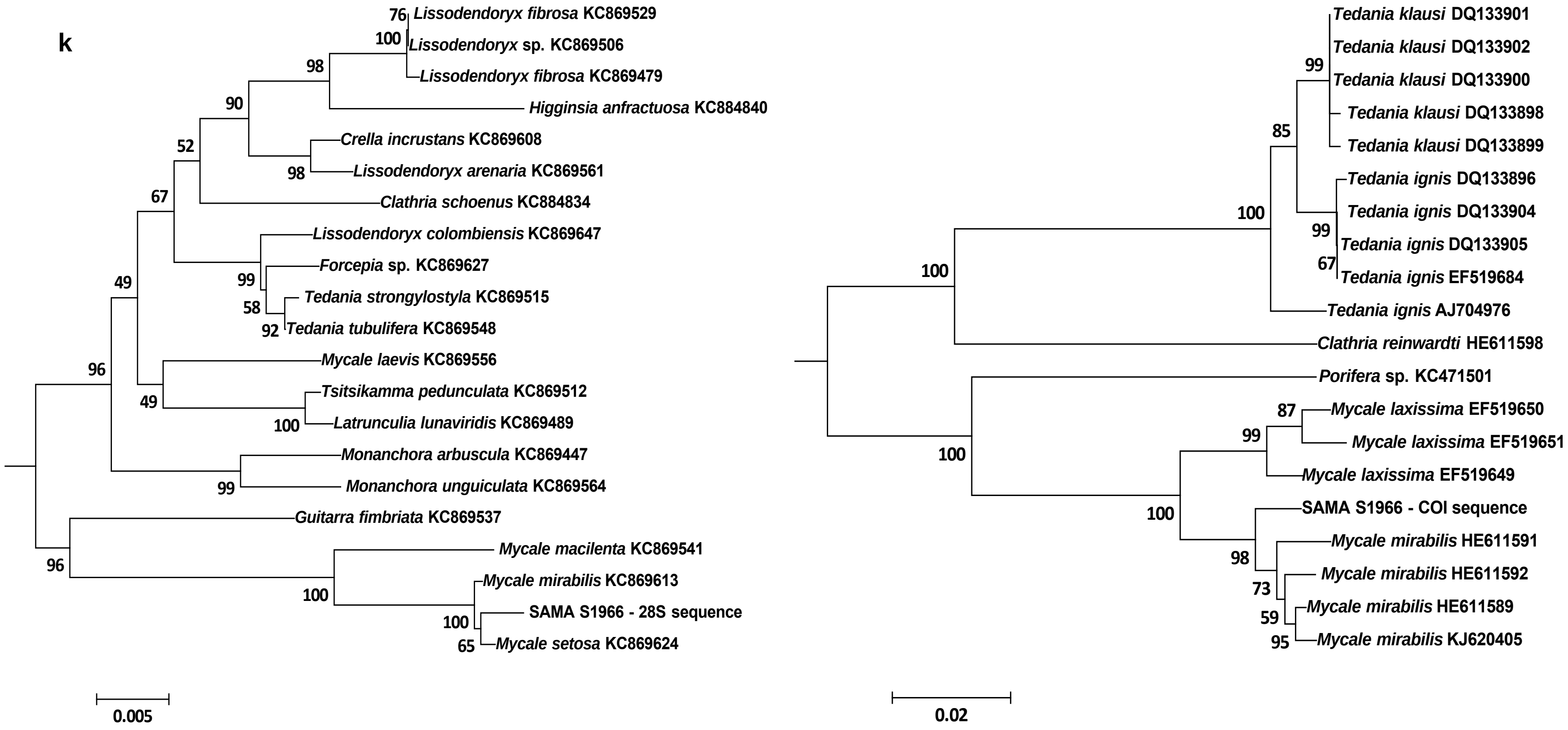
k
Lissodendoryx fibrosa KC869529
Lissodendoryx sp. KC869506
Lissodendoryx fibrosa KC869479
Higginsia anfractuosa KC884840
Crella incrustans KC869608
Lissodendoryx arenaria KC869561
Clathria schoenus KC884834
Lissodendoryx colombiensis KC869647
Forcepia sp. KC869627
Tedania strongylostyla KC869515
Tedania tubulifera KC869548
Mycale laevis KC869556
Tsitsikamma pedunculata KC869512
Latrunculia lunaviridis KC869489
Monanchora arbuscula KC869447
Monanchora unguiculata KC869564
Guitarra fimbriata KC869537
Mycale macilenta KC869541
Mycale mirabilis KC869613
SAMA S1966 - 28S sequence
Mycale setosa KC869624
0.005
Tedania klausi DQ133901
Tedania klausi DQ133902
Tedania klausi DQ133900
Tedania klausi DQ133898
Tedania klausi DQ133899
Tedania ignis DQ133896
Tedania ignis DQ133904
Tedania ignis DQ133905
Tedania ignis EF519684
Tedania ignis AJ704976
Clathria reinwardti HE611598
Porifera sp. KC471501
Mycale laxissima EF519650
Mycale laxissima EF519651
Mycale laxissima EF519649
SAMA S1966 - COI sequence
Mycale mirabilis HE611591
Mycale mirabilis HE611592
Mycale mirabilis HE611589
Mycale mirabilis KJ620405
0.02

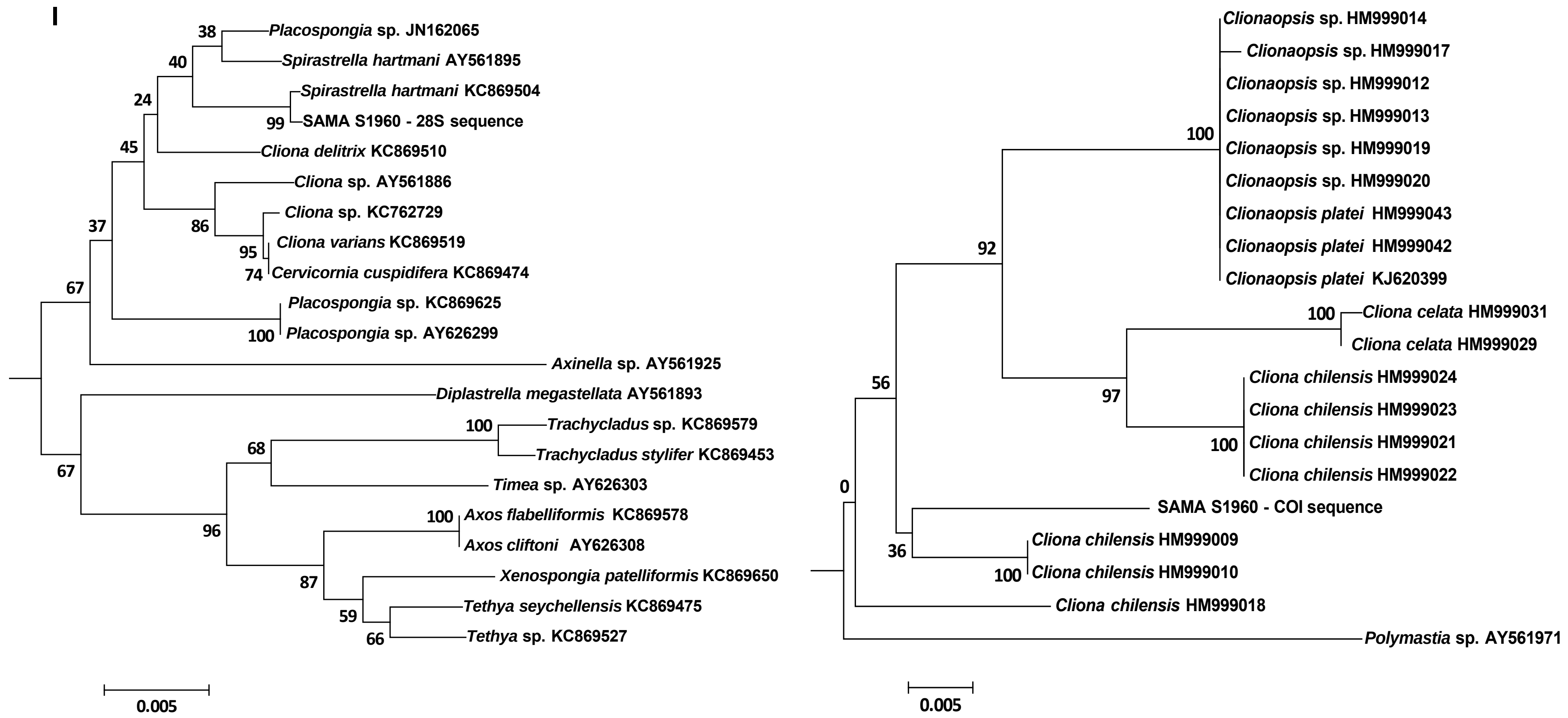
I
Placospongia sp. JN162065
Spirastrella hartmani AY561895
Spirastrella hartmani KC869504
SAMA S1960 - 28S sequence
Cliona delitrix KC869510
Cliona sp. AY561886
Cliona sp. KC762729
Cliona varians KC869519
Cervicornia cuspidifera KC869474
Placospongia sp. KC869625
Placospongia sp. AY626299
Axinella sp. AY561925
Diplastrella megastellata AY561893
Trachycladus sp. KC869579
Trachycladus stylifer KC869453
Timea sp. AY626303
Axos flabelliformis KC869578
Axos cliftoni AY626308
Xenospongia patelliformis KC869650
Tethya seychellensis KC869475
Tethya sp. KC869527
0.005
Clionaopsis sp. HM999014
Clionaopsis sp. HM999017
Clionaopsis sp. HM999012
Clionaopsis sp. HM999013
Clionaopsis sp. HM999019
Clionaopsis sp. HM999020
Clionaopsis platei HM999043
Clionaopsis platei HM999042
Clionaopsis platei KJ620399
Cliona celata HM999031
Cliona celata HM999029
Cliona chilensis HM999024
Cliona chilensis HM999023
Cliona chilensis HM999021
Cliona chilensis HM999022
SAMA S1960 - COI sequence
Cliona chilensis HM999009
Cliona chilensis HM999010
Cliona chilensis HM999018
Polymastia sp. AY561971
0.005

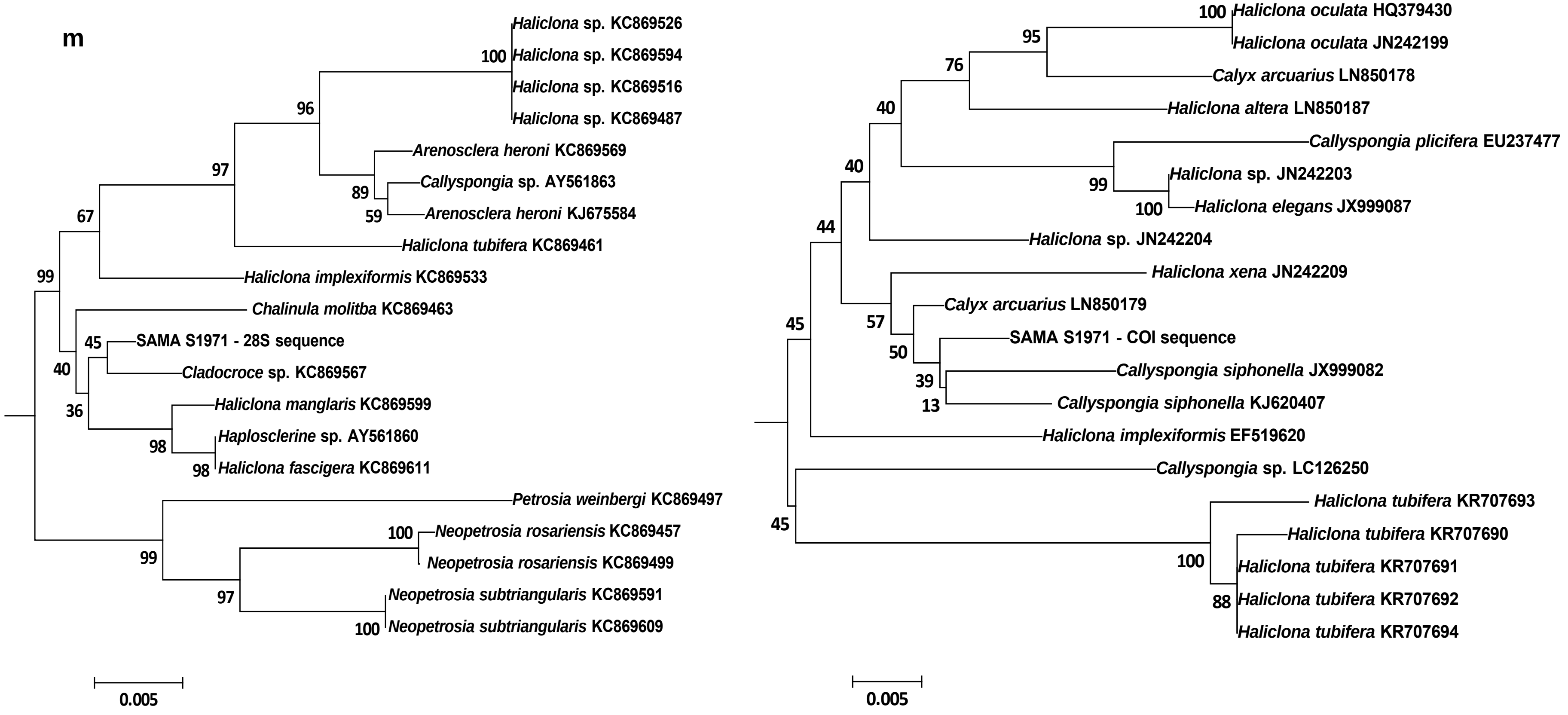
m
Haliclona sp. KC869526
Haliclona sp. KC869594
Haliclona sp. KC869516
Haliclona sp. KC869487
Arenosclera heroni KC869569
Callyspongia sp. AY561863
Arenosclera heroni KJ675584
Haliclona tubifera KC869461
Haliclona implexiformis KC869533
Chalinula molitba KC869463
SAMA S1971 - 28S sequence
Cladocroce sp. KC869567
Haliclona manglaris KC869599
Haplosclerine sp. AY561860
Haliclona fascigera KC869611
Petrosia weinbergi KC869497
Neopetrosia rosariensis KC869457
Neopetrosia rosariensis KC869499
Neopetrosia subtriangularis KC869591
Neopetrosia subtriangularis KC869609
0.005
Haliclona oculata HQ379430
Haliclona oculata JN242199
Calyx arcuarius LN850178
Haliclona altera LN850187
Callyspongia plicifera EU237477
Haliclona sp. JN242203
Haliclona elegans JX999087
Haliclona sp. JN242204
Haliclona xena JN242209
Calyx arcuarius LN850179
SAMA S1971 - COI sequence
Callyspongia siphonella JX999082
Callyspongia siphonella KJ620407
Haliclona implexiformis EF519620
Callyspongia sp. LC126250
Haliclona tubifera KR707693
Haliclona tubifera KR707690
Haliclona tubifera KR707691
Haliclona tubifera KR707692
Haliclona tubifera KR707694
0.005

n

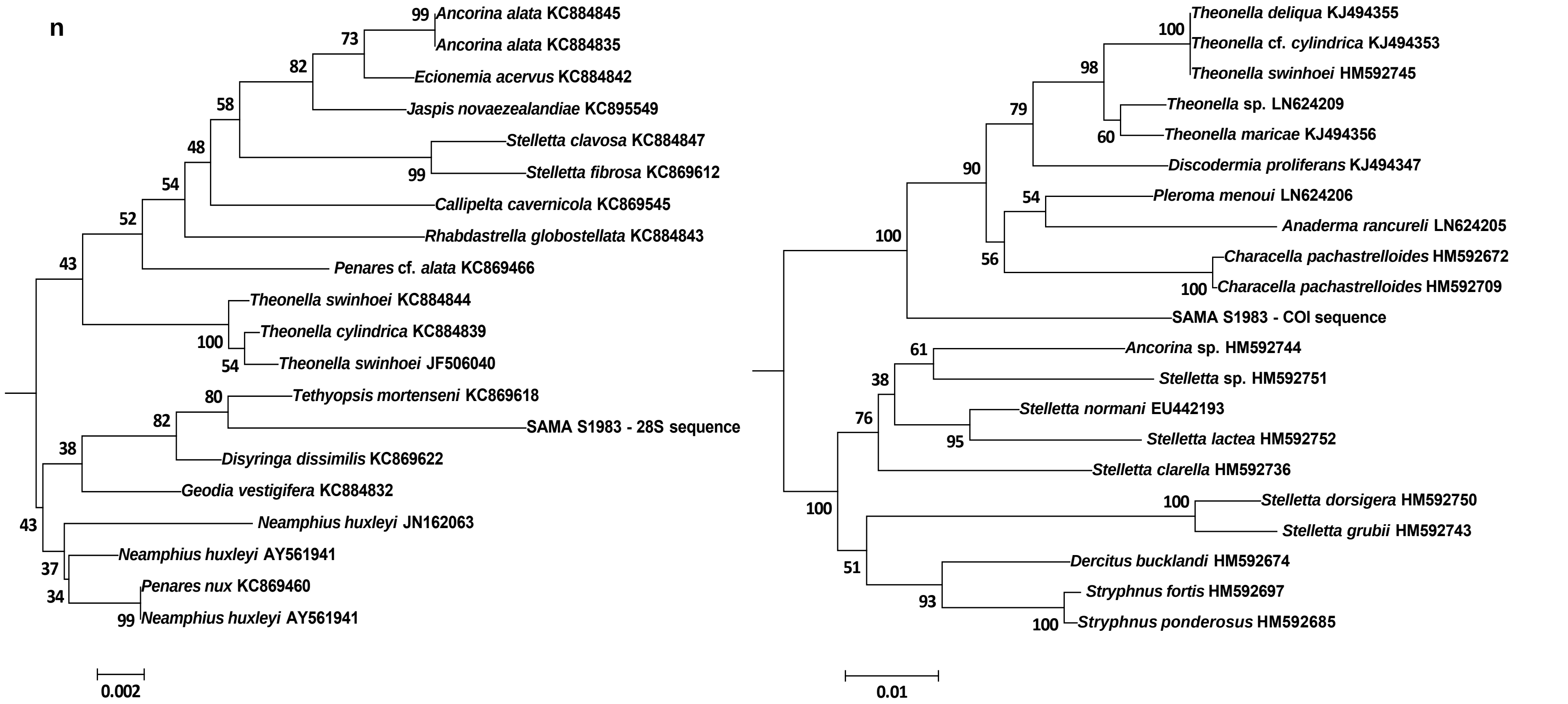

Supplementary Figure S2. Phylogenetic relationship of fourteen sponges using Neighbor-Joining method based on 28S rRNA gene and COI mtDNA. a. Phylogenetic relationship of sponge SAMA S1981. **b.** Phylogenetic relationship of sponge SAMA S1963. **c.** Phylogenetic relationship of sponge SAMA S1965. **d.** Phylogenetic relationship of sponge SAMA S1973. **e.** Phylogenetic relationship of sponge SAMA S1989. **f.** Phylogenetic relationship of sponge SAMA S1961. **g.** Phylogenetic relationship of sponge SAMA S1962. **h.** Phylogenetic relationship of sponge SAMA S1991. **i.** Phylogenetic relationship of sponge SAMA S1982. **j.** Phylogenetic relationship of sponge SAMA S1994. **k.** Phylogenetic relationship of sponge SAMA S1966. **l.** Phylogenetic relationship of sponge SAMA S1960. **m.** Phylogenetic relationship of sponge SAMA S1971. **n.** Phylogenetic relationship of sponge SAMA S1983.
